# Comparative efficacy and safety of talazoparib plus enzalutamide and other first-line treatments for metastatic castration-resistant prostate cancer

**DOI:** 10.1093/oncolo/oyae237

**Published:** 2024-10-19

**Authors:** Elena Castro, Jenna Ellis, Samantha Craigie, Anja Haltner, Jonathan Nazari, Alexander Niyazov, Imtiaz A Samjoo

**Affiliations:** Hospital Universitario 12 de Octubre, Madrid 28041, Spain; EVERSANA™, Burlington, ON, CanadaL7N 3H8; EVERSANA™, Burlington, ON, CanadaL7N 3H8; EVERSANA™, Chicago, IL 60601, United States; Pfizer, Inc., New York, NY 10001, United States; Pfizer, Inc., New York, NY 10001, United States; EVERSANA™, Burlington, ON, CanadaL7N 3H8

**Keywords:** metastatic castration-resistant prostate cancer, network meta-analysis, systematic literature review, PARP inhibitor, talazoparib

## Abstract

**Background:**

Talazoparib plus enzalutamide (TALA + ENZA) has demonstrated antitumor activity in the phase 3 clinical trial (TALAPRO-2; NCT03395197) as first-line (1L) therapy in men with asymptomatic or mildly symptomatic metastatic castration-resistant prostate cancer (mCRPC). Although many active interventions are available, randomized controlled trials (RCTs) involving talazoparib have only been conducted to assess its efficacy and safety compared to enzalutamide. To estimate comparisons between all relevant interventions, indirect comparisons are needed.

**Objective:**

To estimate the comparative efficacy and safety of TALA + ENZA in 1L patients with mCRPC by conducting a systematic literature review and network meta-analyses (NMAs).

**Methods:**

Databases were searched using Ovid, along with several gray literature sources to identify RCTs evaluating treatments in 1L mCRPC (PROSPERO registration: CRD42021283512). Feasibility assessment evaluated trial suitability for NMA inclusion and Bayesian or frequentist NMAs were conducted for evaluable efficacy and safety outcomes, respectively.

**Results:**

Thirty-three RCTs met the eligibility criteria and were feasible for NMAs. Across multiple efficacy outcomes assessed, except for overall survival (OS), TALA + ENZA was ranked the most efficacious treatment. For OS, TALA + ENZA showed the second-highest probability of being the most effective treatment; second to docetaxel 50 mg plus prednisolone 10 mg. With respect to safety outcomes, TALA + ENZA, in general, showed increased rates of hematological adverse events.

**Conclusions:**

TALA + ENZA showed favorable results across multiple efficacy endpoints, but not across hematological toxicities compared with other 1L treatments in asymptomatic or mildly symptomatic mCRPC in the all-comers patient population.

## Teaser

This network meta-analysis assesses the relative efficacy and safety of current treatment options for first-line metastatic castration-resistant prostate cancer. Results of this analysis can aid clinicians in making informed decisions between newer therapies and those already available in clinical practice.

**Implications for practice**

New treatments continue to emerge for metastatic castration-resistant prostate cancer (mCRPC). To facilitate evidence-based decision making, comparisons of all relevant therapeutic interventions are essential. Randomized controlled trials (RCTs) are the gold standard; however, it’s impractical for RCTs to compare against all the available therapies, particularly in mCRPC where the treatment landscape is populated with multiple interventions. Consequently, we conducted a network meta-analysis to comprehensively assess the relative efficacy and safety of current treatment options for first-line mCRPC. Results of this analysis can aid clinicians in making informed decisions between newer therapies and those already available in clinical practice.

## Introduction

Metastatic castration-resistant prostate cancer (mCRPC) is a form of advanced prostate cancer where the cancer no longer responds to treatments that lower testosterone levels, leading to its spread to other parts of the body, often to bone, thus contributing to poor prognosis and a poor quality of life. Despite the significant advances in the management of the disease, mCRPC remains incurable.

Several pharmacological treatments are available for patients with mCRPC in the first-line (1L) setting. Talazoparib is an orally bioavailable poly (ADP-ribose) polymerase inhibitor (PARPi) that inhibits PARP catalytic activity, resulting in DNA breaks. Additionally, talazoparib can trap the PARP enzyme on damaged DNA preventing repair, replication, and transcription, making it a potent PARP trapper.^1^ PARP1 activity has been shown to support the function of androgen receptor (AR) inhibitors such as enzalutamide, suggesting that co-blockade of PARP1 may synergize with AR-directed therapy, regardless of DNA damage response (DDR) alteration status. A combination of talazoparib with enzalutamide (TALA + ENZA) in mCRPC has the potential to improve clinical outcomes in all patients with or without DDR alterations.^2^ The efficacy and safety of TALA + ENZA compared to placebo plus enzalutamide is currently being evaluated in a randomized, double-blind, phase 3 clinical trial (TALAPRO-2; NCT03395197) for men with asymptomatic or mildly symptomatic mCRPC with no systemic treatments initiated after documentation of mCRPC.^3^ At the planned primary analysis, TALA + ENZA resulted in clinically meaningful and statistically significant improvement in radiographic progression-free survival (rPFS) compared to standard of care placebo plus enzalutamide in the unselected all-comers population, but particularly in patients with defects in the homologous recombination repair (HRR) pathway.^4^

The increasing complexity of the treatment landscape represents a challenge when recommending therapeutic options for individual patients. Network meta-analysis (NMA) allows for the simultaneous comparison of multiple treatment options, in the absence of head-to-head RCTs, by combining direct and indirect evidence with an aim of obtaining effectiveness estimates for all possible treatment comparisons within a network.^5^ Relative estimates of treatment effects are essential for both clinicians and payers, who must choose between newer therapies and those already available in clinical practice. The objective of this analysis was to estimate the comparative efficacy and safety of TALA + ENZA (all-comers) to other treatments in 1L patients with asymptomatic or mildly symptomatic mCRPC through NMAs.

## Material and methods

### Systematic literature search

Implementation and reporting of the systematic literature review (SLR) was conducted in accordance with the Preferred Reporting Items for Systematic Literature Reviews and Meta-Analyses (PRISMA) statement.^6,7^ The review protocol was developed in accordance with the PRISMA for systematic review protocols (PRISMA-P) statement^8,9^ and registered with the International Prospective Register of Systematic Reviews a priori (registration number: CRD42021283512). Additional SLR material and methods, including the search strategy (**Supplementary Table S1**), Population, Intervention, Comparator, Outcome, Study design (PICOS) criteria (**Supplementary Table S2**), the sources searched (**Supplementary Figure S1**) and a list of excluded studies (**Supplementary Table S3**) are provided in **Supplementary Appendix A**.

### Feasibility assessment

The validity of results generated by NMAs based on summary-level published data is dependent on the evidence meeting the exchangeability assumption.^10^ Under this assumption, all interventions being studied could have been included as comparators in a clinical trial. Failure to meet this assumption can result in biased estimates of comparative effect. As such, a rigorous qualitative assessment of between-trial heterogeneity was conducted on the data from all relevant studies identified from the systematic review based on published recommendations regarding the assessment of NMA feasibility.^11-13^ If studies were sufficiently similar in terms of patient populations, outcomes assessed, interventions, comparators, and whether they contributed to a connected network, we conducted NMAs where feasible. Networks were developed, considering the available data, similarity of trials and outcome measures. Networks were presented as diagrams that depict treatments as nodes and individual studies as links (**Supplementary Appendix B****—****Supplementary Table S4**; **Supplementary Appendix C****—****Supplementary Figures S2****-****16**).

### Outcomes

Outcomes assessed in this analysis included rPFS, overall survival (OS), time to prostate-specific antigen (PSA) progression, time to cytotoxic chemotherapy initiation, PSA response, and objective response rate (ORR). Based on clinical opinion, 5 adverse events (AE) of special interest (AESI) were assessed in this analysis: anemia, asthenia, fatigue, nausea, and decreased appetite. Both all grade and grade ≥3 AESIs were assessed.

### Network meta-analysis

Network meta-analyses were conducted for efficacy outcomes of interest using a Bayesian framework as described in the National Institute for Health and Care Excellence (NICE) Evidence Synthesis Decision Support Unit (DSU) Technical Support Document (TSD) series.^14^ The most recent data cutoff (DCO) for TALAPRO-2 Cohort 1 (all-comers) (DCO: 28/03/23 for OS and DCO: 16/08/22 for all other outcomes) were used for the present analysis. Analysis of comparator studies also used the most mature data identified in the searches. For the PROpel trial, this NMA used results based on DCO 12/10/22 for both investigator-assessed rPFS and OS, 30/07/21 for both time to PSA progression and PSA response, and DCO 14/03/22 for ORR. Investigator-assessed rPFS was used for PROpel since rPFS by blinded independent central review (BICR) was not available for the latest reported DCO at the time of the analysis. The same approach was applied for all included trials where the latest data were used, regardless of assessment method. When both were available, BICR-assessed data were used. To enable comparison of treatments via a connected network, several assumptions were made^15^; these assumptions were supported by clinical opinion and similar to a previously published NMA (**Supplementary Appendix D**). Both fixed-effect and random-effects models were applied to each efficacy outcome. Random-effects models were conducted for the primary analyses because it makes less stringent assumptions about the consistency of effects,^16^ and so are more appropriate in situations where there are cross-trial differences. A sensitivity analysis using a random-effects model was conducted for rPFS and/or OS whereby trial(s) were removed from their respective network(s) if there was potential for cross-trial heterogeneity identified in the feasibility assessment and based on clinical opinion, provided the remaining trials contributed to a connected network with TALA + ENZA. All analyses were conducted using R version 4.1.2, Just Another Gibbs Sampler version 4.3.0, and WinBUGS version 1.4.3, and were based on burn-in and sampling durations of 60 000 iterations each. Point estimates and 95% credible intervals (CrIs) were modeled for outcomes using Markov Chain Monte Carlo methods. We generated probability of being best (p-best) and the Surface Under the Cumulative Ranking curve (SUCRA), which are measures of effect commonly presented for Bayesian NMAs.^17^ The SUCRA, expressed as a percentage, is the relative probability of an intervention being among the best options or better than other interventions.^17^ For interpretation, both p-best and SUCRA values range between 0 and 1, and values nearer to 1 are preferred.^17^

For time-to-event outcomes (ie, rPFS, OS, time to PSA progression, time to cytotoxic chemotherapy initiation), mean hazard ratio (HR) and its 95% confidence interval (CI) was preferentially extracted for these outcomes. Log-HR and its standard error (SE) was derived for the analysis by taking the natural log (Ln) of the mean HR and dividing the width of Ln of the CI limits by 1.96 × 2. For studies that only reported a Kaplan-Meier (KM) curve, HR and SE data were generated using the algorithm by Guyot et al^18^ Treatment effects were modeled on the log hazard ratio scale with a normal likelihood and an identity link with results reported as hazard ratios. For binary response outcomes (ie, PSA response, ORR) odds ratio (OR) and its 95% CI was preferentially extracted. Studies reporting only number of responders or percentage of response had ORs calculated using contingency tables. Treatment effects were modeled with a binomial likelihood and a logit link on the log odds ratio scale with results reported as odds ratios. In both cases, vague priors for treatment effects, and an informative prior distribution for between-trial variances (pharmaceutical versus pharmaceutical interventions; cause-specific mortality/major morbidity event: *τ*2 ~ log normal [–3.95, 1.792]).^19,20^ were used.

Assessment of absolute model fit was based on the comparison of residual deviance (ResDev) to the number of unconstrained data points and assessment of relative model fit was based on the deviance information criterion (DIC). Convergence and efficiency were assessed using R-hat (a value of <1.05 was considered acceptable), bulk effect sample size (a value of>400 was considered acceptable), and tail effective sample size (a value >400 was considered acceptable).^21^

Network meta-analyses for safety outcomes were conducted in a frequentist framework using a penalized likelihood NMA (PL-NMA) as described in Evrenoglou et al^22^ A PL-NMA was used to attempt to reduce the bias of the maximum likelihood estimate (MLE) that is known to occur in the presence of rare events.^22^ For safety outcomes, event counts were preferentially extracted. Studies reporting only the percentage of patients experiencing an AE had event counts calculated. Since follow-ups across studies were not consistent and a longer follow-up was likely to result in more AESI events, a binomial model with a complementary log-link (cloglog) function was used to account for the variable treatment duration between trials. As such, treatment effects were outputted as discrete hazard ratios (HRs).

Fixed-effect models only were applied to safety outcomes given that very few studies informed each connection and that the method of instituting random effects in PL-NMA has the potential to become dominated by larger studies^23^ while also insufficiently estimating the heterogeneity.^22^

## Results

### Literature search

The searches identified 48 unique RCTs that fulfilled the eligibility criteria after full-text review and removal of duplicates. After the exclusion of studies without published data at the time of the last update, a total of 38 unique RCTs remained (**Supplementary Appendix E****—****Supplementary Figures S17****-****19**).

Data for TALA + ENZA was provided by Pfizer at the time of review given the efficacy and safety of TALA + ENZA were still being evaluated. Since the last SLR update (October 2022), results for the TALAPRO-2 trial have been published (DCO: 16/08/22).^4^ Updated OS data (DCO: 28/03/23) used in the present analysis were provided by Pfizer. Thus, a total of 39 unique RCTs were considered for NMAs and were the primary focus of this analysis.

Of the 39 included trials, 30 RCTs with available full-text publications were assessed for study quality. Several trials did not report the full details of randomization, concealment, and blinding, and so the risk was unclear. Reporting of randomization, allocation, blinding, analysis, and interpretation of results were of moderate quality (**Supplementary Appendix F****—****Supplementary Table S5**).

### Feasibility assessment

Six trials were excluded after the feasibility assessment and supported by clinical opinion. MAGNITUDE permitted patients that were not treatment naïve in the mCRPC disease stage that is, allowed patients to receive 4 months or less of abiraterone acetate therapy and reported data only for a cohort of HRR biomarker positive (BM+) patients; therefore, it was considered inappropriate to include MAGNITUDE in any outcome network because all other trials in the evidence base evaluated an all-comers population. Five additional trials were excluded from all analyses due to failure to connect to any of the main outcome networks.^24-28^ Although there were some differences in methodology and patient populations, the remaining 33 trials were deemed sufficiently similar to derive reasonable estimates of comparative efficacy and safety (**Table 1**). For additional details, see **Supplementary Appendix G****and****H**. A sensitivity analysis was conducted whereby the PROSTY trial evaluating docetaxel 50 mg/m^2^ every 2 weeks was removed from the evidence base. This dose is reserved for frail patients with comorbidities in whom larger doses of docetaxel (ie, 75 mg/m^2^) are intolerable. For additional details, see **Supplementary Appendix L**.

**Table 1.** Summary of included randomized controlled trials baseline characteristics.

Trial; NCT	Weighted average of active treatment and control arms												
	Age (median years)	Caucasian (%)	ECOG PS 0-1 (%)	Gleason score ≥ 8 (%)	Baseline PSA (median ng/mL)	Time since initial ddiagnosis (median years)	BPI-SF ≤ 3 (%)	Bone metastases (%)	Visceral metastasis				
									Total	Lung or liver	Node	Liver	Lung
TALAPRO-2; NCT03395197	71	61.9	100	70.1	17.2	2.84	99.4	Bone: 83.8	NR	15.7	39	3.5	13.2
PROpel; NCT03732820	69.5	70	100	65.7	17.36	3.05	74.6	Bone: 86.5	NR	NR	31.7^a^	4.1	10.3
BRCAAway; NCT03012321	67	90	NR	NR	14.4	NR	NR	Bone only: 50.8	NR	NR	NR	NR	NR
SPARE; NCT02077634	75	NR	99	49	25.7	NR	97.1	Bone only: 43	NR	NR	NR	NR	NR
NCT04862091	71	NR	NR	NR	NR	NR	NR	NR	NR	NR	NR	NR	NR
COU-AA-302; NCT00887198	70.5	NR	NR	52	39.97	5.33	NR	Bone only: 49.5	NR	49.5	NR	NR	NR
NCT01591122	70.3 ^b^	22	100	55	50.89	3.36	NR	Bone: 94 Bone only: 72	NR	NR	NR	NR	NR
NCT01867710	69.5	NR	99.5	57.2	48.28	6.23	NR	Bone: 81.5	NR	NR	49.9	1.2	1.9
STAAR; NCT02737332	75.1 ^b^	75.5	NR	54.7	NR	NR	NR	NR	NR	NR	NR	NR	NR
Hu et al, 2020	NR	NR	NR	NR	NR	NR	NR	NR	NR	NR	NR	NR	NR
PREVAIL; NCT01212991	71.5	77.1	100	51.5	49.26	5.31	98.4	Bone only: 39.8	11.8	NR	NR	NR	NR
NCT02294461	71 ^b^	0	100	65.7	59.29	2.54	100	Bone: 93.3	NR	20.1	50.1	NR	NR
TERRAIN; NCT01288911	71	92.5	100	56.5	21.51	3.10	NR	Bone only: 47	NR	NR	NR	NR	NR
STRIVE; NCT01664923	73	83.1	100	49.8	12.10	6.35	NR	Bone: 54.8 Bone only: 32.1	NR	0	62	3.5	8.5
Alliance A031201; NCT01949337	NR	83	100	NR	NR	NR	NR	NR	NR	NR	NR	NR	NR
NCT02125357	75.3	NR	83	NR	36	NR	NR	Bone: 82.5	NR	NR	NR	6	8.5
ACIS; NCT02257736	71	75	100	NR	31.75	4.45	NR	Bone: 85 Bone only: 42	14.5	NR	47.5	4	10.5
ODENZA; NCT03314324	72	NR	NR	NR	NR	NR	NR	NR	NR	NR	NR	NR	NR
CALGB 9182	72	90.5	NR	NR	145.43	3.35	NR	Bone: 90.5	NR	NR	19	12.6	9
Berry et al, 2002	71	89	99	NR	64.27	NR	NR	Bone: 82	NR	NR	18	1.9	4.1
TAX 327	68.3	NR	87 ^c^	30	115.02	NR	NR	Bone: 91	22.7	NR	NR	NR	NR
NCT00436839	70.8	NR	86.8	40.4	85.58	1.98	NR	Bone: 85.5	18	NR	NR	NR	NR
PROSTY; NCT00255606	68.5	NR	94	NR	112.44	NR	NR	Bone: 87.5	NR	NR	47.1	7	5.5
TIPC	71	NR	88.1	NR	145.74	4.38	NR	Bone: 48	NR	NR	NR	NR	NR
FIRSTANA; NCT01308567	68.5	93.1	95.9	NR	76.65	NR	NR	Bone: 89.5	NR	NR	53.6	9.1	13.3
NCT02218606	68	NR	100	NR	NR	NR	NR	NR	NR	NR	NR	NR	NR
NCT02254785	67.7	NR	93.6	NR	29.59	NR	NR	Bone: 84.2	NR	NR	NR	17.8	24
D9901; NCT00005947	72.3	90.5	100	40.9	46.67	NR	NR	Bone only: 37	0 ^d^	0 ^d^	0 ^d^	0 ^d^	0 ^d^
D9902A; NCT01133704	70.3	91.8	100	NR	55.47	NR	NR	Bone only: 41.8	0 ^d^	0 ^d^	0 ^d^	0 ^d^	0 ^d^
IMPACT; NCT00065442	71.3	90	100	24.6	50.20	7.10	NR	Bone only: 48.2	NR	NR	NR	NR	NR
ERA 223; NCT02043678	71	70.5	99	59.5	30.50	NR	93	Bone: 100	0 ^d^	0 ^d^	0 ^d^	0 ^d^	0 ^d^
EORTC 1333/PEACEIII; NCT02194842	NR	NR	NR	NR	NR	NR	NR	NR	NR	NR	NR	NR	NR
IPATential150; NCT03072238	69.5	69.5	100	63	25.33	2.85	NR	Bone: 84	NR	12	39.5	NR	NR

^a^PROpel reported both distant and locoregional lymph node metastases. The weighted average of active and treatment arms for distant lymph node metastases and locoregional lymph node metastases was 31.7% and 21.5%, respectively. Value for distant lymph node metastases is reported in the table.

^b^Mean value was reported.

^c^Karnofsky > 70% (equivalent to ECOG 0-1), as calculated from Karnosky ≤ 70%: 14% (mitoxantrone 12 mg/m^2^ plus prednisone 5 mg twice daily) 12 % (docetaxel 30 mg/m^2^ every week plus prednisone 5 mg twice daily), 13% (docetaxel 75 mg/m^2^ every 3 weeks plus prednisone 5 mg twice daily).

^d^The presence of visceral metastases was an exclusion criterion.

Abbreviations: BPI-SF, Brief Pain Inventory-Short Form; ECOG PS, Eastern Cooperative Oncology Group Performance Score; mg, milligram; ng/mL, nanogram/milliliter; NR, not reported; PS, performance status; PSA, prostate-specific antigen.

### Network meta-analysis

Results for NMAs using the random-effects model for efficacy outcomes, and fixed-effect model for safety outcomes relative to TALA + ENZA are presented below; all additional random-effects and fixed-effect models are provided in **Supplementary Appendix I****and****J**. No evidence of inconsistency was identified for any of the outcomes (**Supplementary Appendix K**).

#### Radiographic progression-free survival

**Figure 1.**
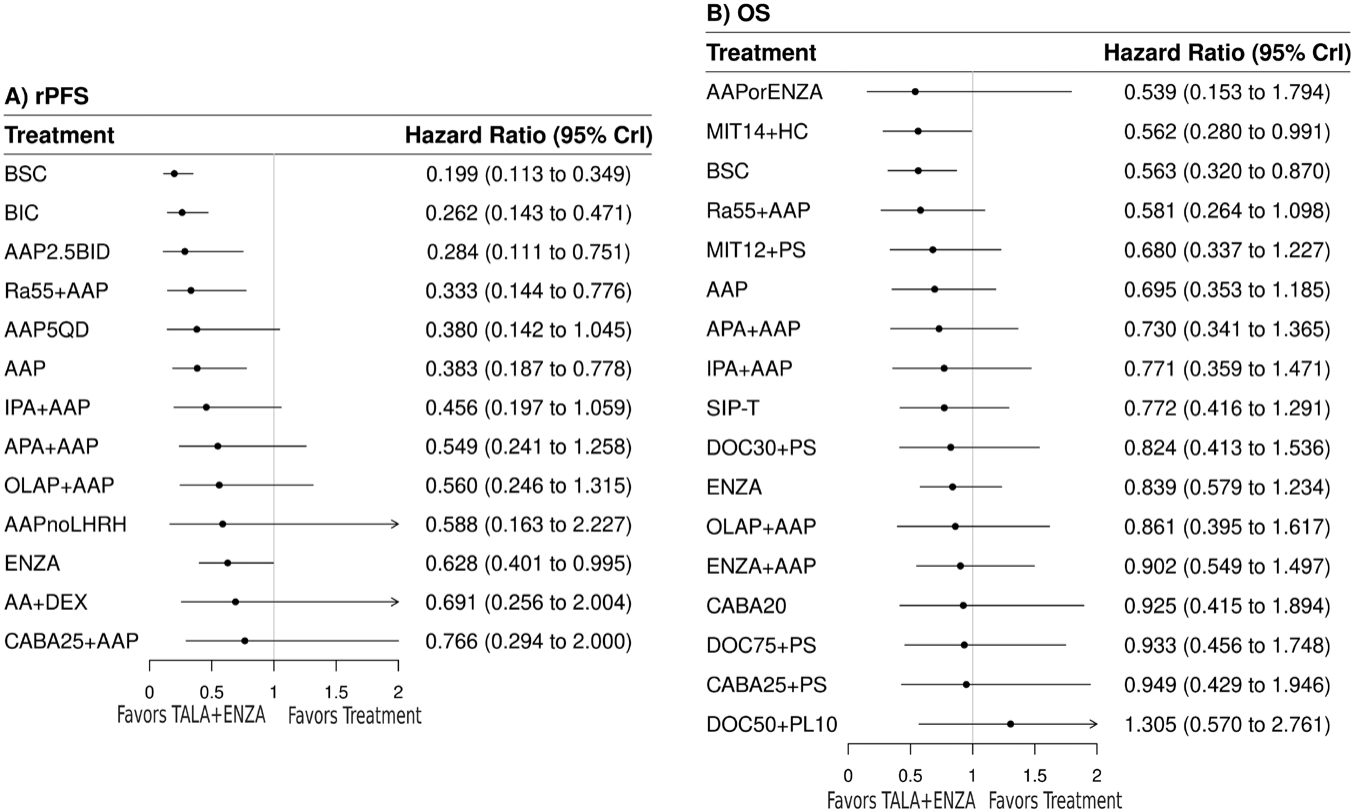
Random-effects forest plots (TALA + ENZA vs. active treatments) for rPFS and OS. Abbreviations: OS, overall survival; rPFS, radiographic progression-free survival. For full list of treatment regimens, refer to Appendix B.

**Figure 2.**
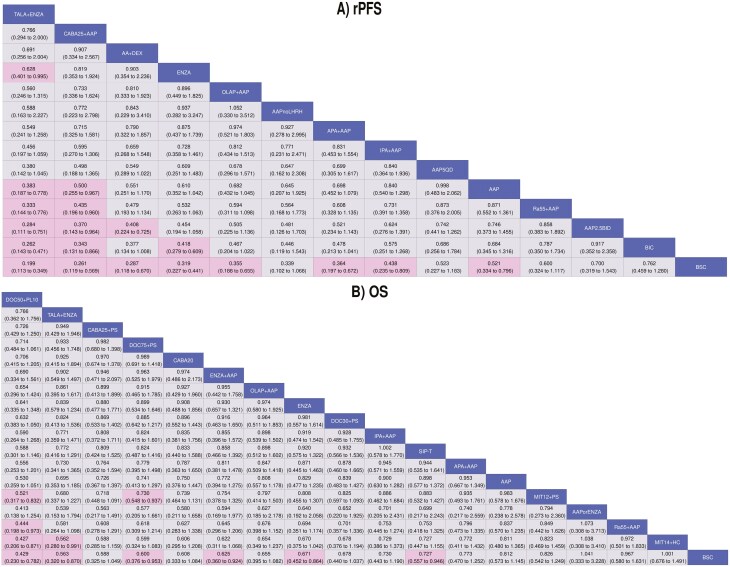
Random-effects league tables for rPFS and OS. Abbreviations: OS, overall survival; rPFS, radiographic progression-free survival. Values are HRs (95% credible interval) for relative effectiveness for all possible pairs of treatments in the network. HR < 1 implies that column is better than row. Pink squares are statistically significant. For full list of treatment regimens, refer to Appendix B.

Thirteen trials encompassing 14 treatments informed the rPFS network. The random-effects model suggested that TALA + ENZA was numerically favored over all treatments, and statistically superior to six treatments (**Figure 1A**). A league table summarizing all pairwise comparisons between treatments is presented in **Figure 2A**. TALA + ENZA exhibited the highest probability (p-best: 52%) of being the most effective treatment, and the highest likelihood of being the top-ranked therapy (SUCRA: 93%) among those compared (**Supplementary Table S9**).

#### Overall survival

Twenty trials encompassing 18 treatments informed the OS network. The random-effects model suggested that TALA + ENZA was numerically favored over all but one treatment (docetaxel 50 mg/m^2^ every 2 weeks plus prednisolone 10 mg once daily), and statistically superior to 2 treatments (**Figures 1B and 2B**). TALA + ENZA exhibited a p-best of 15% and SUCRA of 77% among those compared (**Supplementary Table S10**). The sensitivity analysis removing docetaxel 50 mg/m^2^ (ie, PROSTY trial) suggested that TALA + ENZA was numerically superior to all treatments and represented the top-ranked therapy (**Supplementary Appendix L**).

#### Time to prostate-specific antigen progression

**Figure 3.**
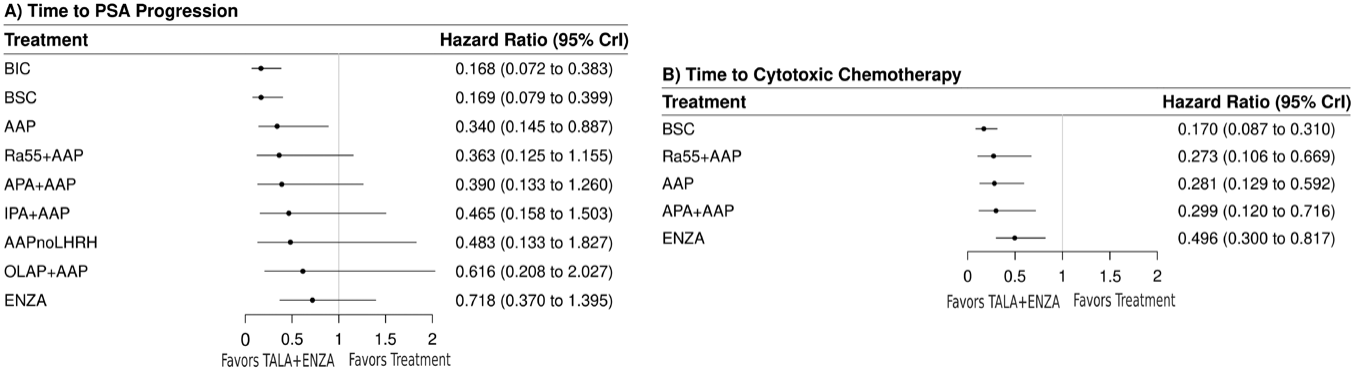
Random-effects forest plots (TALA + ENZA vs. active treatments) for time to PSA progression and time to cytotoxic chemotherapy initiation. Abbreviations: CrI, credible interval; PSA, prostate-specific antigen. For full list of treatment regimens, refer to Appendix B.

Thirteen trials connecting 10 treatments informed the time to PSA progression network. The random-effects model suggested that TALA + ENZA was numerically favored over all treatments and statistically superior to 3 treatments (**Figure 3A**, **Supplementary Figure S20**). TALA + ENZA exhibited the highest probability (p-best: 70%) of being the most effective treatment, and the highest likelihood of being the top ranked therapy (SUCRA: 93%) among those compared (**Supplementary Table S11**).

#### Time to cytotoxic chemotherapy initiation

Seven trials connecting 6 treatments informed the time to cytotoxic chemotherapy initiation network. The random-effects model suggested that TALA + ENZA was statistically superior to all treatments (**Figure 3B**, **Supplementary Figure S21**). TALA + ENZA exhibited the highest probability (p-best: 98%) of being the most effective treatment, and the highest likelihood of being the top-ranked therapy (SUCRA: 99%) (**Supplementary Table S12**).

#### Prostate-specific antigen response

**Figure 4.**
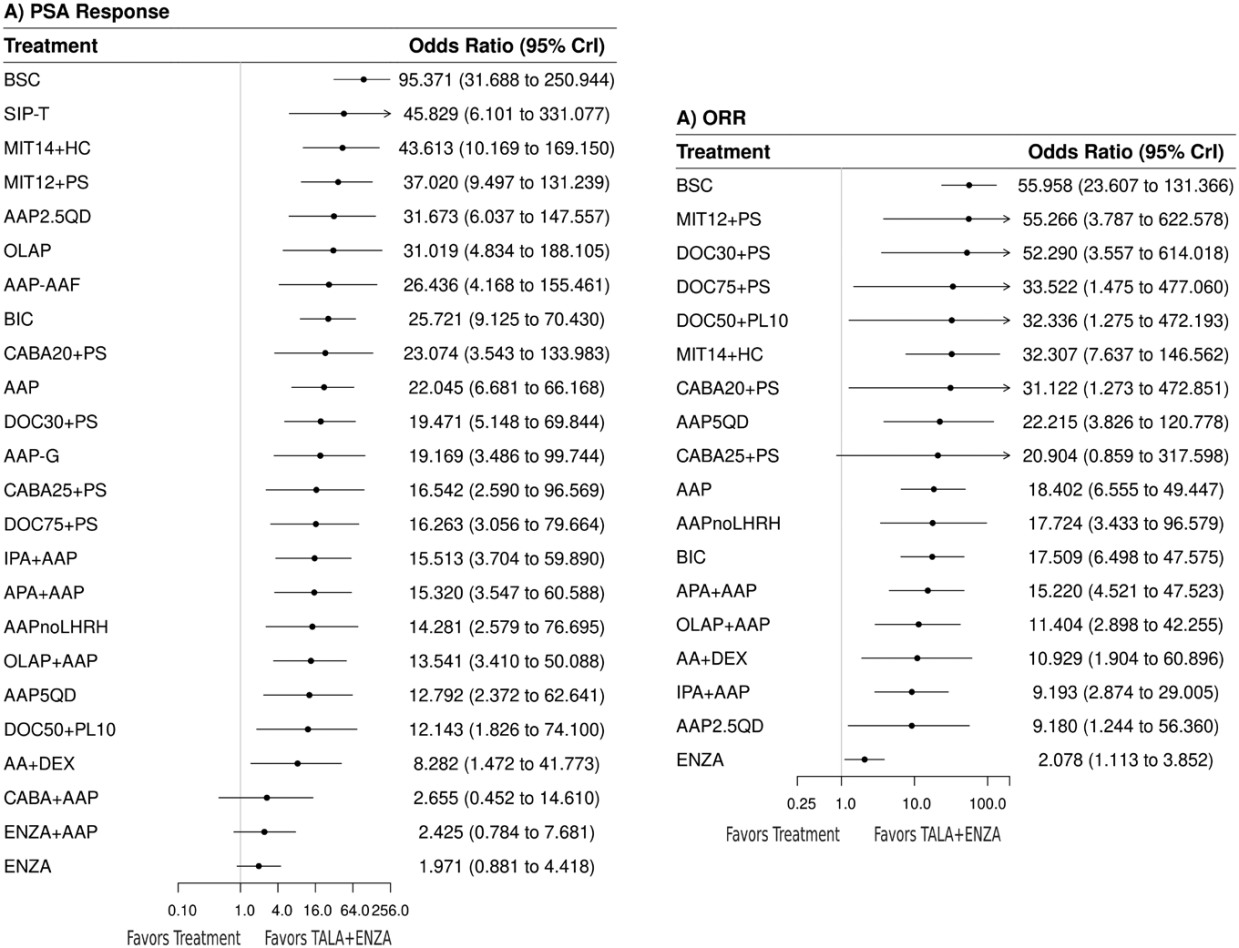
Random-effects forest plot (TALA + ENZA vs. active treatments) for PSA response and ORR. Abbreviations: CrI, credible interval; ORR, objective response rate; PSA, prostate-specific antigen. For full list of treatment regimens, refer to Appendix B.

Twenty-six trials encompassing 25 treatments informed the PSA response network. The random-effects model suggested that TALA + ENZA was numerically favored over all treatments (**Figure 4A**). TALA + ENZA was statistically superior to 21 of the 24 comparators assessed (**Figure 4A**, **Supplementary Figure S22**). TALA + ENZA exhibited the highest probability (p-best: 83%) of being the most effective treatment, and the highest likelihood of being the top ranked therapy (SUCRA: 99%) (**Supplementary Table S13**).

#### Objective response rate

Eighteen trials encompassing 19 treatments informed the ORR network. The random-effects model suggested that TALA + ENZA was statistically superior to all but one comparator, cabazitaxel 25 mg/m^2^ every 3 weeks plus prednisone 5 mg twice daily/10 mg once daily (**Figure 4B**, **Supplementary Figure S23**). TALA + ENZA exhibited the highest probability (p-best: 94%) of being the most effective treatment, and the highest likelihood of being the top-ranked therapy (SUCRA: 99%) (**Supplementary Table S14**).

#### Adverse events of special interest

##### Anemia

**Figure 5.**
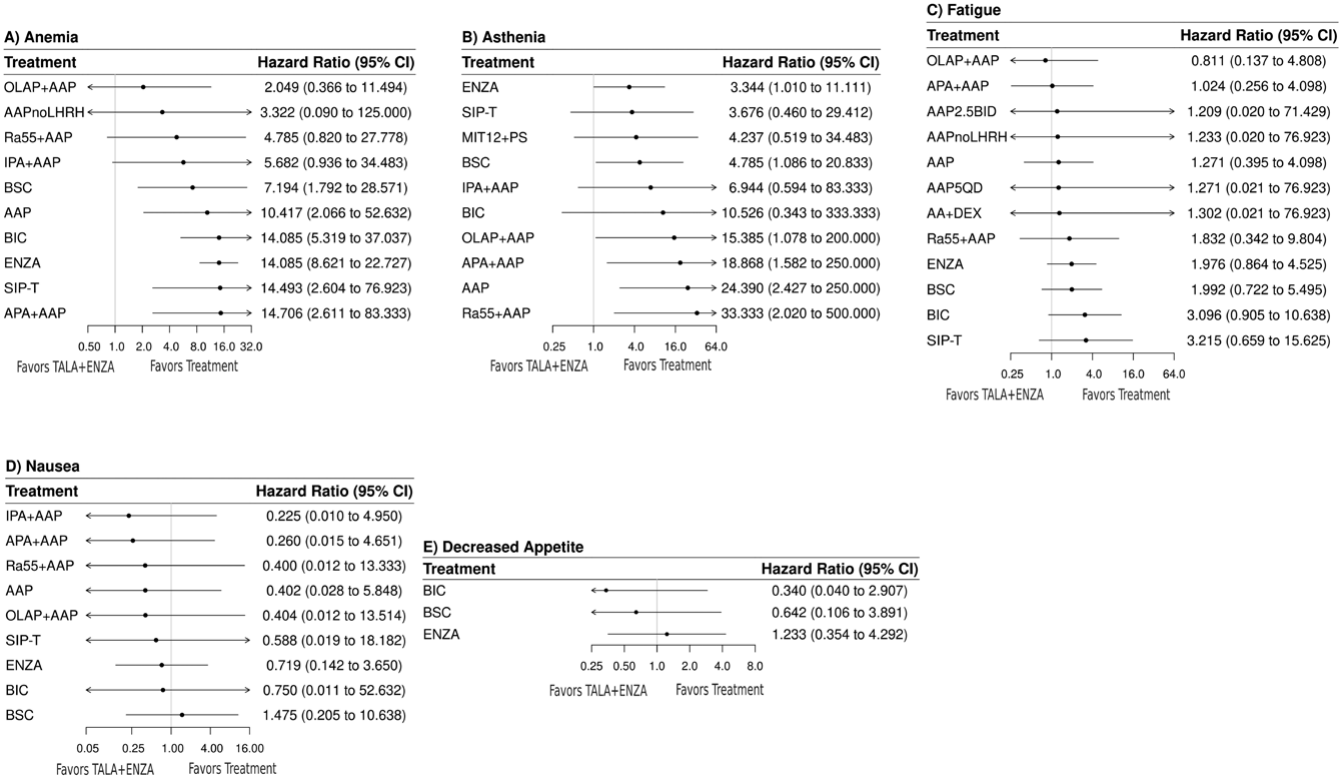
Fixed-effect forest plot (TALA + ENZA vs. active treatments) for grade ≥ 3 AESI. Abbreviations: AESI, adverse events of special interest; CI, confidence interval. For full list of treatment regimens, refer to Appendix B.

Twelve trials encompassing 11 treatments informed the grade ≥3 anemia network. TALA + ENZA showed increased rates of anemia compared to all comparators (**Figure 5A**). A league table summarizing all pairwise comparisons for grade ≥3 anemia between treatments is presented in **Supplementary Figure S40**.

##### Asthenia

Ten trials encompassing 11 treatments informed the grade ≥3 asthenia network. TALA + ENZA showed increased rates of asthenia compared to all comparators (**Figure 5B**). Similar results were observed for all grade asthenia (**Supplementary Figure S30**). A league table summarizing all pairwise comparisons for all grade and grade ≥3 between treatments is presented in **Supplementary Figures S41****and****S42**.

##### Fatigue

Fourteen trials encompassing 13 treatments informed the grade ≥3 fatigue network. TALA + ENZA showed increased rates of fatigue compared to all comparators, except one (olaparib 300 mg once daily plus abiraterone acetate 1000 mg once daily plus prednisone/prednisolone 5 mg twice a day/10 mg once daily), but none of the comparisons were statistically significant (**Figure 5C**). Results for all grade fatigue are presented in **Supplementary Figure S31**. A league table summarizing all pairwise comparisons for all grade and grade ≥ 3 between treatments is presented in **Supplementary Figures S43****and****S44**.

##### Nausea

Ten trials encompassing 10 treatments informed the grade ≥3 nausea network. TALA + ENZA showed decreased rates of nausea compared to all comparators assessed except best supportive care; none of the comparisons were statistically significant (**Figure 5D**). Results for all grade nausea are presented in **Supplementary Figure S32**. A league table summarizing all pairwise comparisons for all grade and grade ≥ 3 between treatments is presented in **Supplementary Figures S45****and****S46**.

##### Decreased appetite

Four trials encompassing 4 treatments informed the grade ≥3 decreased appetite network. TALA + ENZA showed decreased rates of decreased appetite compared to all comparators except enzalutamide; none of the results were statistically significant (**Figure 5E**). Results for all grade decreased appetite are presented in **Supplementary Figure S33**. A league table summarizing all pairwise comparisons for all grade and grade ≥3 between treatments is presented in **Supplementary Figures S47****and****S48**.

## Discussion

Several novel agents have been developed for mCRPC, demonstrating efficacy in 1L asymptomatic or mildly symptomatic patients, including TALA + ENZA which has shown a survival benefit versus enzalutamide in the TALAPRO-2 study^4^ but has not been compared directly to other treatments in clinical studies. To facilitate evidence-based healthcare decision making, comparisons of all relevant therapeutic interventions are required. Nevertheless, in the absence of RCTs directly comparing treatments, an alternative approach is necessary to evaluate and compare the available clinical data. NMAs provide useful evidence to help select the most appropriate therapy option(s); this was the approach selected in our analysis to estimate the relative efficacy and safety of TALA + ENZA. At the time of this analysis, this was the first NMA of RCTs comparing different treatments across contemporary and historical trials for first-line asymptomatic or mildly symptomatic mCRPC.

Data from 33 RCTs were included in these NMAs. The results of these analyses demonstrated that TALA + ENZA had superior efficacy compared to several alternative treatment options across several efficacy outcomes assessed. In all instances, TALA + ENZA was at least numerically favorable over competing drugs and exhibited the highest probability of being the most effective treatment and the highest likelihood of being the top-ranked therapy apart from docetaxel 50 mg/m^2^ every 2 weeks plus prednisolone 10 mg once daily (DOC50 + PL10) with regards to OS. The OS results are somewhat unexpected given the median OS for TALA + ENZA from TALAPRO-2 was not estimable versus 19.5 months for DOC50 + PL10 from PROSTY despite TALAPRO-2 having a longer median follow-up time (35.8 vs 18 months, respectively).^29^ The reasons for this are unclear but may be due to the inherent use of relative HRs as inputs for these analyses.

Importantly, this dose of docetaxel was evaluated in one trial (PROSTY^29^) which demonstrated statistically superior OS (HR: 1.4; 95% CI, 1.1-1.8; *P* = .021) and better tolerability compared to the standard dose of docetaxel (75 mg/m^2^ every 3 weeks). The low dose is frequently reserved for old and frail patients with comorbidities who are unlikely to tolerate large single doses of docetaxel^29,30^ which is in alignment with prostate cancer guidelines.^31^ Importantly, this trial was powered for the endpoint time to treatment failure (TTTF) and was analyzed in the per protocol population; thus, OS results should be interpreted with caution. Results of the sensitivity analysis removing the PROSTY trial suggested that when DOC50 + PL10 was removed from the OS network, TALA + ENZA represented the top-ranked therapy and was numerically superior to all other 1L treatments. Nonetheless, available OS data in the TALAPRO-2 study is not yet mature and as survival follow-up is continuing, a future re-analysis of this NMA should be conducted.

Abiraterone, enzalutamide, docetaxel, sipuleucel-T, and radium-223 are regimens that are recommended in several mCRPC guidelines. Relative to these treatment regimens for 1L mCRPC, the novel PARPi, talazoparib plus enzalutamide (in all-comers) were numerically superior and represented the top-ranked therapy for OS (when PROSTY was excluded: Supplementary Appendix L); these results were not statistically significant. Of note, the second-most probable treatment regimen was another PARPi, olaparib plus abiraterone acetate, followed by docetaxel, enzalutamide, sipuleucel-T, abiraterone, and radium-223.

Safety analyses demonstrated inferior results for TALA + ENZA for most of the adverse events assessed. A statistically significant increased rate of grade ≥3 anemia and asthenia were noted with TALA + ENZA compared to other treatment options. Hematological toxicity is a class effect of PARPi that is related to PARP trapping. No significant differences in grade ≥3 gastrointestinal toxicities were observed between TALA + ENZA and other treatment options.

Previous NMAs have also compared the efficacy of treatments for chemotherapy-naïve patients with asymptomatic or mildly symptomatic mCPRC^15,32^; however, they were conducted at a time when trials evaluating novel PARP inhibitor therapies for mCRPC were in development and with a limited or narrower review; therefore, direct comparisons with our analysis are challenging. Given the recent advances in the treatment of 1L mCRPC, our NMA specifically provides data on the comparative efficacy and safety of newer therapies for key clinical endpoints commonly assessed in prostate cancer clinical trials.

A notable strength of this analysis was the rigorous qualitative assessment of cross-trial heterogeneity conducted a priori and supported by clinical opinion. To ensure the underlying assumptions of the analysis were systematically explored and the risks and benefits of indirectly comparing treatment effects are clear and transparent, the similarity of included studies identified by the SLR was assessed. In the absence of international guidelines on how to assess similarity in this context, guidance on best practice, for the conduct of indirect treatment comparisons was leveraged.^11-13^ Our strict adherence to the population of first-line asymptomatic or mildly symptomatic ensured greater certainty in the treatment effect estimates—other NMAs incorporated trials with mixed populations including patients who were not truly treatment naïve for mCRPC, or they were more symptomatic in terms of pain.^15^ Although adherence to the strict eligibility criteria is certainly a strength of the current analysis in efforts to create a more homogenous population, we acknowledge that it may not reflect real-world heterogeneity in clinical practice. All relevant comparators for TALA + ENZA were included in the analysis, and this reflects approved and expected therapies for the treatment of first-line mCRPC up to now. Furthermore, with the inclusion of the PARPi—talazoparib and olaparib, this analysis reflects the most contemporary treatment landscape. We acknowledge that that at the time of writing the more likely “effective” regimens (as determined by our analysis) may not be readily available in certain jurisdictions and this must be considered when interpreting these results; however, this manuscript may serve as the impetus for reimbursement/policy change in those markets with the understanding that market authorization/approval and reimbursement is multifactorial. Despite the approval of TALA + ENZA as an initial treatment for patients with mCRPC with DNA repair gene alterations by the United States Food and Drug Administration, we elected to perform the NMAs with patients included in Cohort 1 of TALAPRO-2 (ie, all-comers), irrespective of HRR status, because the proportion of patients with HRR alterations was unknown in all the other included trials. Finally, each NMA was conducted in accordance with the methodology recommended by NICE to ensure transparency and reproducibility.^14^

This study is not without limitations. Indirect treatment comparisons such as NMAs rely on the assumption that trials are sufficiently similar such that the effect estimate will not be biased by underlying differences in patient populations. In this analysis, trial design and patient eligibility criteria were relatively similar, but between-trial heterogeneity was observed in some baseline patient characteristics. Despite efforts to minimize bias and heterogeneity, such as the feasibility assessment and clinical expert consultation, there are unobserved heterogeneity or covariates that cannot be adjusted or controlled for, such as differences in study populations. We have considered these between-trial differences acceptable based on clinical input, but it is important to note that the impact of the heterogeneous characteristics has not been explored in this study, which represents an important area of further research. The use of individual patient data to conduct adjusted analyses such as matching-adjusted indirect comparison (MAIC) may be instrumental in closing this knowledge gap, considering the sparsely connected network (ie, treatment comparisons being informed by only one trial) precluding meta-regression. Additionally, the evidence networks in the present analysis are sparse, that is, each treatment comparison, except for a few, is informed by a single RCT. Furthermore, because of the therapeutic advances in mCRPC and change in treatment practices over time, the evidence networks are not centered around a single common comparator, resulting in some treatments being connected through multiple nodes. Residual heterogeneity, multinode connectivity, and network sparsity all contribute to large variability and wide confidence intervals in the treatment effect estimates, contributing to low power to declare statistical significance. Several assumptions were necessary to facilitate network connection, for example, the assumption that all trial placebo/corticosteroid arms were considered in the same network node regardless of the route of administration. As previously mentioned, the present NMA included a comprehensive list of first-line treatments for patients with mCRPC. While this is considered a strength in our study, some non-standard treatment doses were included in the networks which may not be clinically relevant for clinicians and other stakeholders. Although best practices were employed to conduct these NMAs based on industry standards, the results must be considered with caution due to the unobserved variability. Any clinical decision making is based on multiple sources of evidence, and the present analyses provide one source in the current landscape due to the limited breadth of data. The Prostate Cancer Clinical Trials Working Group 3 (PCWG3) provides CRPC recommendations for standardizing the conduct of clinical trials.^33^ The outcomes chosen in this NMA were decided following a feasibility assessment, in which some efficacy, safety, and patient-reported outcomes were deemed infeasible to estimate due to a dearth of data or lack of consistency in reporting/definition across all included trials. However, several endpoints recommended by the PCWG3 were feasible and included in the present analysis, including rPFS, OS, time to PSA progression, time to cytotoxic chemotherapy initiation, PSA response, ORR, and AEs. Notably, PROs, which the PCWG3 recognizes the importance of, and other potential novel endpoints were not addressed in the present study. Clinical decision making involves a holistic view of clinical efficacy, risk, and patient preferences and values. The present effect estimates generated by NMAs are not meant to substitute RCT data but in the absence of head-to-head trial data they provide an additional source of evidence to help aid clinical decision making. Finally, we used random-effects model for our main efficacy analysis, consequently producing estimates with relatively wide credible intervals compared with those generated using a fixed-effect model due to the less stringent assumptions (ie, is more conservative) about the consistency of effects. However, the random-effects model is the most suitable to account for the residual cross-trial imbalances present in our broad evidence base, in accordance with NICE guidance.^14,34,35^ Nevertheless, a holistic approach was adopted, including all relevant trials, because implementation of strict criteria could have excluded the majority from the network.

Research in metastatic prostate cancer is abundant and is advancing the field forward with radiopharmaceuticals such as lutetium-177 (^177^Lu)-prostate-specific membrane antigen (PSMA)-617 which have the potential to increase treatment options for patients. As radiopharmaceuticals, immunotherapies, new treatments and innovative combinations of these treatments emerge, the importance of direct head-to-head RCTs cannot be underscored, but in such absence, NMAs will continually need updating with the advent of these technologies and will remain as one of a multitude of sources of evidence to guide clinical decisions.

## Conclusion

New treatment options continue to emerge for patients with mCRPC; hence, there is a need to disseminate clinical efficacy and safety knowledge constantly and rapidly to help individuals make up-to-date and well-informed healthcare decisions. Here, we used NMA to synthesize the current treatment landscape for 1L patients with asymptomatic or mildly symptomatic mCRPC and incorporated recently introduced treatments to gain a comprehensive and contemporary view of the comparative efficacy and safety of these drugs. Our results demonstrated that TALA + ENZA offers a therapeutic benefit over other 1L treatments approved or expected to be approved across multiple clinical endpoints, indicating its therapeutic potential in the all-comers patient population.

## Supplementary material

Supplementary material is available at *The Oncologist* online.

oyae237_suppl_Supplementary_Tables_1-14_Figures_1-48

## Data Availability

No new data were generated or analyzed in support of this research.
