# Supplementary material for: Comparative efficacy and safety of talazoparib plus enzalutamide and other first-line treatments for metastatic castration-resistant prostate cancer
Source: Oncologist. 2024 Oct 19;30(3):oyae237. doi: 10.1093/oncolo/oyae237 (PMC11954501; doi:10.1093/oncolo/oyae237)
Supplement: oyae237_suppl_Supplementary_Tables_1-14_Figures_1-48 [file oyae237_suppl_supplementary_tables_1-14_figures_1-48.docx]

# Supplementary Materials

1. Material and Methods

**Systematic Literature Search**

The search was conducted on 2021 September 9 and updated on 2022 February 17 and 2022 October 3. Using the Ovid® search interface, the following electronic databases were searched: Embase, Ovid MEDLINE® (including Epub Ahead of Print and In-Process & Other Non-Indexed Citations), Ovid MEDLINE® Daily, Cochrane Central Register of Controlled Trials, and the Cochrane Database of Systematic Reviews. Search strategies utilized a combination of controlled vocabulary (e.g., “prostatic neoplasms”) and keywords (e.g., "metastatic castration-resistant prostate cancer”). Vocabulary and syntax were adjusted across databases. Modified versions of the Cochrane Highly Sensitive Search Strategy filter for identifying RCTs in MEDLINE® and Embase[^1^](#_ENREF_1) were applied, in addition to filters for SLRs.[^2^](#_ENREF_2)^,^[^3^](#_ENREF_3) Language was not restricted. When possible, animal-only and opinion pieces were removed. Abstracts from conference proceedings identified from the systematic literature search were also included. The MEDLINE® search strategy was peer-reviewed independently by a senior medical information specialist before execution using the Peer Review of Electronic Search Strategies (PRESS) checklist[^4^](#_ENREF_4) (**Supplementary Table 1**). All search strategies were generated utilizing the Population, Intervention, Comparator, and Study Design (PICOS) elements **(Supplementary Table 2)**.

To supplement the database searches, manual check of the reference list of included reviews germane to the scope of this project was conducted. We also supplemented our review of published studies with data from conference proceedings, health technology assessment and regulatory agency documents, and information provided by manufacturers (i.e., Pfizer). For the full list of databases and supplemental sources searched see **Supplementary Figure 1**.

After the latest SLR update, any new published data for relevant comparator trials were captured to ensure the analyses herein incorporated the most recent comparator data. As a result, any relevant updated data presented at the American Society of Clinical Oncology – Genitourinary Cancers Symposium (ASCO-GU) 2023 conference from PROpel[^5^](#_ENREF_5) and MAGNITUDE[^6^](#_ENREF_6), additional information from full-text publications for MAGNITUDE[^7^](#_ENREF_7)^,^[^8^](#_ENREF_8), as well as updated data from TALAPRO-2 provided by Pfizer and subsequent publications from the trial[^9^](#_ENREF_9) were incorporated into the analyses.

**Study Selection and Data Extraction**

Subsequent to the literature search and removal of duplicate citations using local software tools (EndNote Version 20.1 and 20.4; Clarivate, EndNote, Chandler, AZ, USA), study selection was accomplished through two levels of screening, at the abstract and full-text level. Two reviewers independently screened the titles and abstracts of all publications identified using DistillerSR Version 2.35 and 2.41 (DistillerSR Inc. 2021, Ottawa, Canada); a third reviewer worked with the initial two reviewers to resolve any issues of disagreement through consensus. No study was excluded at abstract level screening due to insufficient information.

Citations accepted during abstract-level screening were retrieved in full text for review. Reasons for exclusion were categorized according to the PICOS elements during full-text review (**Supplementary Table 3**). Disagreements were resolved by a third independent reviewer.

For studies meeting the eligibility criteria, data relating to trial design and methodology, details of interventions, patient eligibility criteria, reported baseline characteristics, outcome assessments, and results were extracted. Data extraction was performed by two independent reviewers using a standardized data extraction form designed in Microsoft Excel® (Microsoft Corporation, WA, USA). Discrepancies in collected data were resolved by consensus or a third independent reviewer. Multiple publications identified for the same trial, patient population, and setting that reported data for the same intervention were linked and extracted as a single publication.

**Quality Assessment**

Quality of each RCT was assessed using the NICE Single Technology Appraisal Evidence Submission Checklist for assessment of risk of bias in RCTs.[^10^](#_ENREF_10) Quality assessments were only completed for full-text publications, not conference abstracts, as they did not have sufficient methodological data to assess the study. Study quality assessment was conducted by a single reviewer and a second reviewer validated consistency and integrity of the assessments. Any disagreements were resolved through discussion or by consulting a third reviewer.

Search Strategy

**Date of the search:** 09 Sept 2021

**Database searched:**

- Ovid MEDLINE(R)
- Ovid MEDLINE Epub Ahead of Print, In-Process, In-Data-Review & Other Non-Indexed Citations and Daily
- Ovid Embase
- Ovid EBM Reviews - Cochrane Central Register of Controlled Trials
- Ovid EBM Reviews - Cochrane Database of Systematic Reviews

**Limits:**

- No date and language limit
- Adults only
- Conference abstracts: 2019 - Current

**Filters:**

Randomized Studies (both MEDLINE and Embase filters have Phase 2-3 - additional terms to supplement RCTs filter) from:

Higgins JPT, Thomas J, Chandler J, Cumpston M, Li T, Page MJ, Welch VA (editors). *Cochrane Handbook for Systematic Reviews of Interventions* version 6.2 (updated February 2021). Cochrane, 2021. Available from www.training.cochrane.org/handbook.

**MEDLINE, sensitivity- and precision-maximizing version – Cochrane Handbook, 2019**

**Box 3.d, e** Cochrane Highly Sensitive Search Strategy for identifying randomized trials in MEDLINE and Embase: sensitivity- and precision-maximizing version (2008 revision); Ovid format https://training.cochrane.org/handbook/current/chapter-04-technical-supplement-searching-and-selecting-studies#section-3-6-1

**Embase sensitive Filter – Cochrane Handbook, 2019**

**Box 3.e** Cochrane Highly Sensitive Search Strategy for identifying controlled trials in Embase: (2018 revision) https://training.cochrane.org/handbook/current/chapter-04-technical-supplement-searching-and-selecting-studies#section-3-6-2

**SLR filters**

1. Ovid Expert Searches: translated from PubMed and added to Ovid MEDLINE as a subset limit in 2019
2. modified Montori VM, et al. (Hedges Team). BMJ. 2005 Jan 8;330(7482):68. PMCID: PMC543864.
3. Additional terms for MA, NMA, ITC

**MULTIFILE SEARCH**

Database(s): **EBM Reviews - Cochrane Central Register of Controlled Trials**August 2021**, EBM Reviews - Cochrane Database of Systematic Reviews**2005 to September 9, 2021**, Embase**1974 to 2021 September 08**, Ovid MEDLINE(R) and Epub Ahead of Print, In-Process, In-Data-Review & Other Non-Indexed Citations and Daily**1946 to September 08, 2021

Table 1: Search Strategy

| **#** | **Searches** | **Results** |
| --- | --- | --- |
| 1 | Prostatic Neoplasms/ | 147205 |
| 2 | Prostatic Neoplasms/ or (((prostate or prostatic) adj3 (adenocarcinoma$ or adeno-carcinoma$ or cancer$ or carcinoma$ or malignan$ or neoplas$ or tumor? or tumour?)) or PC or PCa).ti,ab,kf. | 662314 |
| 3 | Orchiectomy/ and (insensitiv$ or refractor$ or resistan$).ti,ab,kf. | 2335 |
| 4 | (((androgen? or castrat$ or hormon$) adj2 (independen$ or insensitiv$ or refractor$ or resistan$)) or ((orchectom$ or orcheotom$ or orchidectom$ or orchiectom$ or testectom$ or (removal adj3 (testicle? or test#s))) and (insensitiv$ or refractor$ or resistan$))).ti,ab,kf. | 64313 |
| 5 | 2 and (3 or 4) | 47982 |
| 6 | Prostatic Neoplasms, Castration-Resistant/ or (MCRPC or CRPC or MCRPCa or CRPCa or (((prostate or prostatic) adj3 (adenocarcinoma$ or adeno-carcinoma$ or cancer$ or carcinoma$ or malignan$ or neoplas$ or tumor? or tumour?)) and ((androgen? or castrat$ or hormon$) adj2 (independen$ or insensitiv$ or refractor$ or resistan$)))).ti,ab,kf. | 50907 |
| 7 | 5 or 6 | 51186 |
| 8 | exp Neoplasm Metastasis/ or (metasta$ or ((disseminat$ or spread$ or secondary or migrat$ or seeding) adj3 (neoplas$ or cancer$ or carcinoma$ or adenocarcinoma$ or adeno-carcinoma$ or tumo?r? or sarcoma)) or micrometasta$ or advanced or incurable or late stage? or lethal$ or noncurable or non-curable or progressive or stage IV or terminal or uncurable).ti,ab,kf. | 4683323 |
| 9 | 7 and 8 [MCRPC] | 34884 |
| 10 | (talazoparib$2 or Bmn-673 or Bmn-673ts or bmn673 or bmn673ts or lt-006673 or lt-673 or lt006673 or lt673 or mdv-3800 or mdv3800 or talzenna$2 or 1207456-01-6 or 1373431-65-2 or 9QHX048FRV).ti,ab,kw,kf,rn. [TALAZOPARIB TERMS] | 1676 |
| 11 | (rucaparib$ or ag-014699 or ag-14447 or ag-14699 or ag014699 or ag14447 or ag14699 or co-338 or co338 or pf-01367338 or pf-1367338 or pf-1367338-bw or pf01367338 or pf1367338 or pf1367338bw or rubraca$2 or 1859053-21-6 or 283173-50-2 or 459868-92-9 or 8237f3u7eh).ti,ab,kw,kf,rn. [RUCAPARIB TERMS] | 2193 |
| 12 | (olaparib$2 or azd-2281 or azd2281 or azd221 or ku-0059436 or ku-59436 or ku0059436 or ku59436 or nsc-747856 or lynparza or 763113-22-0 or WOH1JD9AR8).ti,ab,kw,kf,rn. [OLAPARIB TERMS] | 9318 |
| 13 | (niraparib$ or gsk-3985771 or gsk3985771 or jnj- 64091742 or jnj64091742 or mk-4827 or mk4827 or zejula or zl-2306 or zl2306 or 1038915-60-4 or 1038915-73-9 or 1613220-15-7 or HMC2H89N35).ti,ab,kw,kf,rn. [NIRAPARIB TERMS] | 2069 |
| 14 | Docetaxel/ or (docetaxel$ or daxotel$2 or dexotel$2 or docetaxol$2 or docefrez$2 or lit-976 or lit976 or nsc-628503 or nsc628503 or oncodocel$2 or rp-56976 or rp56976 or taxespira$2 or taxoter$2 or taxotere$2 or taxoltere$2 or texot$2 or taxoel$2 or xrp6976 or hsdb-6965 or sid-530 or 114977-28-5 or 699121PHCA).ti,ab,kw,kf,rn. [DOCETAXEL TERMS] | 91529 |
| 15 | (cabazitaxel$2 or kabazitaxel$2 or jevtana$2 or rpr-116258-a or rpr-116258a or rpr116258a or txd-258 or txd258 or xrp-6258 or xrp6258 or nsc-761432 or 51F690397J or 183133-96-2).ti,ab,kw,kf,rn. [CABAZITAXEL TERMS] | 4823 |
| 16 | Mitoxantrone/ or (mitoxantron$ or dhad or dhaq or domitrone$2 or elsep$2 or formyxan$2 or genefadrone$2 or misostol$2 or mitoxanthrone$2 or mitoxgen$2 or mitozantrone$2 or mitroxantrone$2 or mitroxone$2 or neotalem$2 or norexan$2 or novanthron$2 or novantron$2 or novantrone$2 or now-85-34 or now-8534 or now8534 or nsc-287836 or nsc-279836 or nsc-301739 or nsc-301739d or nsc287836 or nsc279836 or nsc301739 or nsc301739d or oncotron$2 or onkotrone$2 or quinizarin$2 or ralenova$2 or pralifan$2 or nsc 279836 or BZ114NVM5P or 65271-80-9 or 70476-82-3 or 137635-96-2 or 70945-62-9 or 70711-41-0).ti,ab,kw,kf,rn. [MITOXANTRONE TERMS] | 33266 |
| 17 | (pembrolizumab$2 or keytruda$2 or lambrolizumab$2 or mk-3475 or mk3475 or sch-900475 or sch900475 or Merck-3475 or DPT0O3T46P or 1374853-91-4).ti,ab,kw,kf,rn. [PEMBROLIZUMAB TERMS] | 31440 |
| 18 | (sipuleucelT$2 or sipuleucel-T or apc-8015 or apc8015 or provenge$2 or 8Q622VDR18 or 917381-47-6).ti,ab,kw,kf,rn. [SIPULEUCEL-T TERMS] | 3239 |
| 19 | (enzalutamide$2 or xtandi$2 or mdv-3100 or mdv3100 or hc-1119 or hc1119 or 915087-33-1 or 93T0T9GKNU).ti,ab,kw,kf,rn. [ENZALUTAMIDE TERMS] | 11154 |
| 20 | (apalutamide$2 or arn-509 or arn509 or erleada or 956104-40-8 or 4T36H88UA7).ti,ab,kw,kf,rn. [APALUTAMIDE TERMS] | 1468 |
| 21 | (darolutamide$2 or bay-1841788 or bay1841788 or nubeqa or odm201 or orm-16497 or odm-201 or orm-16555 or 1297538-32-9 or X05U0N2RCO).ti,ab,kw,kf,rn. [DAROLUTAMIDE TERMS] | 688 |
| 22 | Abiraterone Acetate/ or (abiraterone$ or abretone$2 or cb-7630 or cb7630 or yonsa$2 or zytiga$2 or nsc-749227 or nsc-748121 or 154229-18-2 or EM5OCB9YJ6).ti,ab,kw,kf,rn. [ABIRATERONE ACETATE TERMS] | 11808 |
| 23 | (cb-07598 or cb7598 or nsc-741232 or 154229-19-3 or G819A456D0).ti,ab,kw,kf,rn. [ABIRATERONE TERMS] | 5163 |
| 24 | (alpharadin$2 or bay-88-8223 or bay888223 or bay88-8223 or radium223 or radium-223 or radium-ra-223 or radium-ra223 or radiumra-223 or xofigo$2 or ((Ra-223 or 223Ra) adj radioisotope$) or 8BR2SOL3L1 or 15623-45-7).ti,ab,kw,kf,rn. [RADIUM-223 TERMS] | 2900 |
| 25 | (ipatasertib$2 or gdc-0068 or gdc0068 or rg-7440 or rg7440 or 524Y3IB4HQ or 1001264-89-6).ti,ab,kw,kf,rn. [IPATASERTIB TERMS] | 595 |
| 26 | or/10-25 [ALL DRUGS] | 174928 |
| 27 | 9 and 26 | 16887 |
| 28 | exp Animals/ not Humans/ | 17509914 |
| 29 | 27 not 28 [ANIMAL-ONLY REMOVED] | 11764 |
| 30 | (comment or editorial or news or newspaper article).pt. | 2237720 |
| 31 | (letter not (letter and randomized controlled trial)).pt. | 2336459 |
| 32 | 29 not (30 or 31) [OPINION PIECES REMOVED] | 11421 |
| 33 | exp Child/ not (exp Adult/ and exp Child/) | 3340023 |
| 34 | exp Infant/ not (exp Adult/ and exp Infant/) | 1703513 |
| 35 | 32 not (33 or 34) [CHILD AND INFANT - ONLY REMOVED] | 11414 |
| 36 | (clinical trials as topic.sh. or (randomized controlled trial or controlled clinical trial).pt. or (randomized or placebo or randomly).ab. or trial.ti.) not (exp animals/ not humans.sh.) [RANDOMIZED STUDIES – MEDLINE, sensitivity- and precision-maximizing version – Cochrane Handbook, 2019] | 2668233 |
| 37 | exp Randomized Controlled Trials as Topic/ | 370041 |
| 38 | (randomised or randomi#ation? or RCT or placebo*).tw,kf. | 1423197 |
| 39 | ((singl* or doubl* or trebl* or tripl*) adj (mask* or blind* or dumm*)).tw,kf. | 739101 |
| 40 | (equivalence trial or pragmatic clinical trial).pt. | 5350 |
| 41 | Clinical Trial, Phase II/ or Clinical Trial, Phase III/ | 53360 |
| 42 | ((study or trial or CT) adj3 (phase 2 or phase 2a or phase 2b or phase 2c or phase II or phase IIa or phase IIb or phase IIc or phase 3 or phase 3a or phase 3b or phase 3c or phase III or phase IIIa or phase IIIb or phase IIIc or "phase? 2/3" or "phase? II/III")).tw,kf. | 261860 |
| 43 | or/36-42 [RCTs] | 3763845 |
| 44 | 35 and 43 [(m)CRPC -ALL DRUGS- RCTs] | 4476 |
| 45 | 44 use ppez [MEDLINE records] | 1405 |
| 46 | exp prostate cancer/ or (((prostate or prostatic) adj3 (adenocarcinoma$ or adeno-carcinoma$ or cancer$ or carcinoma$ or malignan$ or neoplas$ or tumor? or tumour?)) or PC or PCa).ti,ab,kw. | 718119 |
| 47 | orchiectomy/ and (insensitiv$ or refractor$ or resistan$).ti,ab,kw. | 2347 |
| 48 | (((androgen? or castrat$ or hormon$) adj2 (independen$ or insensitiv$ or refractor$ or resistan$)) or ((orchectom$ or orcheotom$ or orchidectom$ or orchiectom$ or testectom$ or (removal adj3 (testicle? or test#s))) and (insensitiv$ or refractor$ or resistan$))).ti,ab,kw. | 64696 |
| 49 | 46 and (47 or 48) | 48224 |
| 50 | castration resistant prostate cancer/ or (MCRPC or CRPC or MCRPCa or CRPCa or (((prostate or prostatic) adj3 (adenocarcinoma$ or adeno-carcinoma$ or cancer$ or carcinoma$ or malignan$ or neoplas$ or tumor? or tumour?)) and ((androgen? or castrat$ or hormon$) adj2 (independen$ or insensitiv$ or refractor$ or resistan$)))).ti,ab,kw. | 51702 |
| 51 | 49 or 50 | 52045 |
| 52 | exp metastasis/ or (metasta$ or ((disseminat$ or spread$ or secondary or migrat$ or seeding) adj3 (neoplas$ or cancer$ or carcinoma$ or adenocarcinoma$ or adeno-carcinoma$ or tumo?r? or sarcoma)) or micrometasta$ or advanced or incurable or late stage? or lethal$ or noncurable or non-curable or progressive or stage IV or terminal or uncurable).ti,ab,kw. | 4693684 |
| 53 | 51 and 52 [MCRPC] | 35425 |
| 54 | Talazoparib/ or (talazoparib$2 or Bmn-673 or Bmn-673ts or bmn673 or bmn673ts or lt-006673 or lt-673 or lt006673 or lt673 or mdv-3800 or mdv3800 or talzenna$2 or 1207456-01-6 or 1373431-65-2 or 9QHX048FRV).ti,ab,kw,du,dy,tn,rn. [TALAZOPARIB TERMS] | 1719 |
| 55 | Rucaparib/ or (rucaparib$ or ag-014699 or ag-14447 or ag-14699 or ag014699 or ag14447 or ag14699 or co-338 or co338 or pf-01367338 or pf-1367338 or pf-1367338-bw or pf01367338 or pf1367338 or pf1367338bw or rubraca$2 or 1859053-21-6 or 283173-50-2 or 459868-92-9 or 8237f3u7eh).ti,ab,kw,du,dy,tn,rn. [RUCAPARIB TERMS] | 2327 |
| 56 | Olaparib/ or (olaparib$2 or azd-2281 or azd2281 or azd221 or ku-0059436 or ku-59436 or ku0059436 or ku59436 or nsc-747856 or lynparza or 763113-22-0 or WOH1JD9AR8).ti,ab,kw,du,dy,tn,rn. [OLAPARIB TERMS] | 9388 |
| 57 | Niraparib/ or (niraparib$ or gsk-3985771 or gsk3985771 or jnj- 64091742 or jnj64091742 or mk-4827 or mk4827 or zejula or zl-2306 or zl2306 or 1038915-60-4 or 1038915-73-9 or 1613220-15-7 or HMC2H89N35).ti,ab,kw,du,dy,tn,rn. [NIRAPARIB TERMS] | 2134 |
| 58 | Docetaxel/ or (docetaxel$ or daxotel$2 or dexotel$2 or docetaxol$2 or docefrez$2 or lit-976 or lit976 or nsc-628503 or nsc628503 or oncodocel$2 or rp-56976 or rp56976 or taxespira$2 or taxoter$2 or taxotere$2 or taxoltere$2 or texot$2 or taxoel$2 or xrp6976 or hsdb-6965 or sid-530 or 114977-28-5 or 699121PHCA).ti,ab,kw,du,dy,tn,rn. [DOCETAXEL TERMS] | 91529 |
| 59 | Cabazitaxel/ or (cabazitaxel$2 or kabazitaxel$2 or jevtana$2 or rpr-116258-a or rpr-116258a or rpr116258a or txd-258 or txd258 or xrp-6258 or xrp6258 or nsc-761432 or 51F690397J or 183133-96-2).ti,ab,kw,du,dy,tn,rn. [CABAZITAXEL TERMS] | 4858 |
| 60 | mitoxantrone/ or (mitoxantron$ or dhad or dhaq or domitrone$2 or elsep$2 or formyxan$2 or genefadrone$2 or misostol$2 or mitoxanthrone$2 or mitoxgen$2 or mitozantrone$2 or mitroxantrone$2 or mitroxone$2 or neotalem$2 or norexan$2 or novanthron$2 or novantron$2 or novantrone$2 or now-85-34 or now-8534 or now8534 or nsc-287836 or nsc-279836 or nsc-301739 or nsc-301739d or nsc287836 or nsc279836 or nsc301739 or nsc301739d or oncotron$2 or onkotrone$2 or quinizarin$2 or ralenova$2 or pralifan$2 or nsc 279836 or BZ114NVM5P or 65271-80-9 or 70476-82-3 or 137635-96-2 or 70945-62-9 or 70711-41-0).ti,ab,kw,du,dy,tn,rn. [MITOXANTRONE TERMS] | 33269 |
| 61 | Pembrolizumab/ or (pembrolizumab$2 or keytruda$2 or lambrolizumab$2 or mk-3475 or mk3475 or sch-900475 or sch900475 or Merck-3475 or DPT0O3T46P or 1374853-91-4).ti,ab,kw,du,dy,tn,rn. [PEMBROLIZUMAB TERMS] | 31504 |
| 62 | Sipuleucel T/ or (sipuleucelT$2 or sipuleucel-T or apc-8015 or apc8015 or provenge$2 or 8Q622VDR18 or 917381-47-6).ti,ab,kw,du,dy,tn,rn. [SIPULEUCEL-T TERMS] | 3692 |
| 63 | Enzalutamide/ or (enzalutamide$2 or xtandi$2 or mdv-3100 or mdv3100 or hc-1119 or hc1119 or 915087-33-1 or 93T0T9GKNU).ti,ab,kw,du,dy,tn,rn. [ENZALUTAMIDE TERMS] | 11278 |
| 64 | Apalutamide/ or (apalutamide$2 or arn-509 or arn509 or erleada or 956104-40-8 or 4T36H88UA7).ti,ab,kw,du,dy,tn,rn. [APALUTAMIDE TERMS] | 1535 |
| 65 | Darolutamide/ or (darolutamide$2 or bay-1841788 or bay1841788 or nubeqa or odm201 or orm-16497 or odm-201 or orm-16555 or 1297538-32-9 or X05U0N2RCO).ti,ab,kw,du,dy,tn,rn. [DAROLUTAMIDE TERMS] | 722 |
| 66 | Abiraterone Acetate/ or (abiraterone$ or abretone$2 or cb-7630 or cb7630 or yonsa$2 or zytiga$2 or nsc-749227 or nsc-748121 or 154229-18-2 or EM5OCB9YJ6).ti,ab,kw,du,dy,tn,rn. [ABIRATERONE ACETATE TERMS] | 11817 |
| 67 | Abiraterone/ or (abiraterone$2 or cb-07598 or cb7598 or nsc-741232 or 154229-19-3 or G819A456D0).ti,ab,kw,du,dy,tn,rn. [ABIRATERONE TERMS] | 11786 |
| 68 | "radium chloride ra 223"/ or (alpharadin$2 or bay-88-8223 or bay888223 or bay88-8223 or radium223 or radium-223 or radium-ra-223 or radium-ra223 or radiumra-223 or xofigo$2 or ((Ra-223 or 223Ra) adj radioisotope$) or 8BR2SOL3L1 or 15623-45-7).ti,ab,kw,du,dy,tn,rn. [RADIUM-223 TERMS] | 3503 |
| 69 | Ipatasertib/ or (ipatasertib$2 or gdc-0068 or gdc0068 or rg-7440 or rg7440 or 524Y3IB4HQ or 1001264-89-6).ti,ab,kw,du,dy,tn,rn. [IPATASERTIB TERMS] | 624 |
| 70 | or/54-69 [ALL DRUGS] | 175610 |
| 71 | 53 and 70 | 17327 |
| 72 | exp animal/ or exp animal experimentation/ or exp animal model/ or exp animal experiment/ or nonhuman/ or exp vertebrate/ | 54609070 |
| 73 | exp human/ or exp human experimentation/ or exp human experiment/ | 42976108 |
| 74 | 72 not 73 | 11634698 |
| 75 | 71 not 74 [ANIMAL-ONLY REMOVED] | 17008 |
| 76 | editorial.pt. | 1281683 |
| 77 | letter.pt. not (letter.pt. and randomized controlled trial/) | 2336385 |
| 78 | 75 not (76 or 77) [OPINION PIECES REMOVED] | 16621 |
| 79 | exp adolescent/ not (exp adult/ and exp adolescent/) | 1263029 |
| 80 | exp child/ not (exp adult/ and exp child/) | 3340023 |
| 81 | fetus/ not (fetus/ and exp adult/) | 231241 |
| 82 | 78 not (79 or 80 or 81) [UNDER 18 REMOVED] | 16612 |
| 83 | Randomized controlled trial/ or Controlled clinical study/ or randomization/ or intermethod comparison/ or double blind procedure/ or human experiment/ or (compare or compared or comparison or trial).ti. or ((evaluated or evaluate or evaluating or assessed or assess) and (compare or compared or comparing or comparison)).ab. or (random$ or placebo or (open adj label) or ((double or single or doubly or singly) adj (blind or blinded or blindly)) or parallel group$1 or (crossover or cross over) or ((assign$ or match or matched or allocation) adj5 (alternate or group$1 or intervention$1 or patient$1 or subject$1 or participant$1)) or (assigned or allocated) or (controlled adj7 (study or design or trial)) or (volunteer or volunteers)).ti,ab. | 10399744 |
| 84 | (Cross-sectional study/ not (randomized controlled trial/ or controlled clinical study/ or controlled study/ or randomi?ed controlled.ti,ab. or control group$1.ti,ab.)) or ((((case adj control$) and random$) not randomi?ed controlled) or (nonrandom$ not random$) or "Random field$" or (random cluster adj3 sampl$)).ti,ab. or (Systematic review not (trial or study)).ti. or ((review.ab. and review.pt.) not trial.ti.) or ("we searched".ab. and (review.ti. or review.pt.)) or ("update review" or (databases adj4 searched)).ab. or ((rat or rats or mouse or mice or swine or porcine or murine or sheep or lambs or pigs or piglets or rabbit or rabbits or cat or cats or dog or dogs or cattle or bovine or monkey or monkeys or trout or marmoset$1).ti. and animal experiment/) or (Animal experiment/ not (human experiment/ or human/)) | 5270149 |
| 85 | 83 not 84 [RANDOMIZED STUDIES – Embase Filter – Cochrane Handbook, 2019] | 9514189 |
| 86 | "clinical trial (topic)"/ | 114019 |
| 87 | "randomized controlled trial (topic)"/ | 210273 |
| 88 | (RCT or placebo*).ti,ab,kw. | 981147 |
| 89 | ((singl* or doubl* or trebl* or tripl*) adj (mask* or blind* or dumm*)).ti,ab,kw. | 763695 |
| 90 | phase 2 clinical trial/ | 91071 |
| 91 | phase 3 clinical trial/ | 55829 |
| 92 | ((study or trial or CT) adj3 (phase 2 or phase 2a or phase 2b or phase 2c or phase II or phase IIa or phase IIb or phase IIc or phase 3 or phase 3a or phase 3b or phase 3c or phase III or phase IIIa or phase IIIb or phase IIIc or "phase? 2/3" or "phase? II/III")).ti,ab,kw. | 278019 |
| 93 | or/85-91 [RCTs] | 9853893 |
| 94 | 82 and 93 [(m)CRPC - ALL DRUGS - RCTs] | 7880 |
| 95 | conference abstract.pt. | 4191531 |
| 96 | 94 not 95 [CONFERENCE ABSTRACTS REMOVED] | 5187 |
| 97 | 94 and 95 | 2693 |
| 98 | limit 97 to yr="2019-current" | 732 |
| 99 | 96 or 98 [MOST RECENT 2 YEARS CONF ABSTRACTS RETAINED] | 5919 |
| 100 | 99 use oemezd [EMBASE RECORDS] | 3007 |
| 101 | Prostatic Neoplasms/ or (((prostate or prostatic) adj3 (adenocarcinoma$ or adeno-carcinoma$ or cancer$ or carcinoma$ or malignan$ or neoplas$ or tumor? or tumour?)) or PC or PCa).ti,ab,kw. | 672092 |
| 102 | Orchiectomy/ and (insensitiv$ or refractor$ or resistan$).ti,ab,kw. | 2347 |
| 103 | (((androgen? or castrat$ or hormon$) adj2 (independen$ or insensitiv$ or refractor$ or resistan$)) or ((orchectom$ or orcheotom$ or orchidectom$ or orchiectom$ or testectom$ or (removal adj3 (testicle? or test#s))) and (insensitiv$ or refractor$ or resistan$))).ti,ab,kw. | 64696 |
| 104 | 101 and (102 or 103) | 48153 |
| 105 | Prostatic Neoplasms, Castration-Resistant/ or (MCRPC or CRPC or MCRPCa or CRPCa or (((prostate or prostatic) adj3 (adenocarcinoma$ or adeno-carcinoma$ or cancer$ or carcinoma$ or malignan$ or neoplas$ or tumor? or tumour?)) and ((androgen? or castrat$ or hormon$) adj2 (independen$ or insensitiv$ or refractor$ or resistan$)))).ti,ab,kw. | 51065 |
| 106 | 104 or 105 | 51350 |
| 107 | exp Neoplasm Metastasis/ or (metasta$ or ((disseminat$ or spread$ or secondary or migrat$ or seeding) adj3 (neoplas$ or cancer$ or carcinoma$ or adenocarcinoma$ or adeno-carcinoma$ or tumo?r? or sarcoma)) or micrometasta$ or advanced or incurable or late stage? or lethal$ or noncurable or non-curable or progressive or stage IV or terminal or uncurable).ti,ab,kw. | 4693684 |
| 108 | 106 and 107 [MCRPC] | 35005 |
| 109 | (talazoparib$2 or Bmn-673 or Bmn-673ts or bmn673 or bmn673ts or lt-006673 or lt-673 or lt006673 or lt673 or mdv-3800 or mdv3800 or talzenna$2 or 1207456-01-6 or 1373431-65-2 or 9QHX048FRV).ti,ab,kw. [TALAZOPARIB TERMS] | 1041 |
| 110 | (rucaparib$ or ag-014699 or ag-14447 or ag-14699 or ag014699 or ag14447 or ag14699 or co-338 or co338 or pf-01367338 or pf-1367338 or pf-1367338-bw or pf01367338 or pf1367338 or pf1367338bw or rubraca$2 or 1859053-21-6 or 283173-50-2 or 459868-92-9 or 8237f3u7eh).ti,ab,kw. [RUCAPARIB TERMS] | 1276 |
| 111 | (olaparib$2 or azd-2281 or azd2281 or azd221 or ku-0059436 or ku-59436 or ku0059436 or ku59436 or nsc-747856 or lynparza or 763113-22-0 or WOH1JD9AR8).ti,ab,kw. [OLAPARIB TERMS] | 6195 |
| 112 | (niraparib$ or gsk-3985771 or gsk3985771 or jnj- 64091742 or jnj64091742 or mk-4827 or mk4827 or zejula or zl-2306 or zl2306 or 1038915-60-4 or 1038915-73-9 or 1613220-15-7 or HMC2H89N35).ti,ab,kw. [NIRAPARIB TERMS] | 1235 |
| 113 | Docetaxel/ or (docetaxel$ or daxotel$2 or dexotel$2 or docetaxol$2 or docefrez$2 or lit-976 or lit976 or nsc-628503 or nsc628503 or oncodocel$2 or rp-56976 or rp56976 or taxespira$2 or taxoter$2 or taxotere$2 or taxoltere$2 or texot$2 or taxoel$2 or xrp6976 or hsdb-6965 or sid-530 or 114977-28-5 or 699121PHCA).ti,ab,kw. [DOCETAXEL TERMS] | 91523 |
| 114 | (cabazitaxel$2 or kabazitaxel$2 or jevtana$2 or rpr-116258-a or rpr-116258a or rpr116258a or txd-258 or txd258 or xrp-6258 or xrp6258 or nsc-761432 or 51F690397J or 183133-96-2).ti,ab,kw. [CABAZITAXEL TERMS] | 3406 |
| 115 | Mitoxantrone/ or (mitoxantron$ or dhad or dhaq or domitrone$2 or elsep$2 or formyxan$2 or genefadrone$2 or misostol$2 or mitoxanthrone$2 or mitoxgen$2 or mitozantrone$2 or mitroxantrone$2 or mitroxone$2 or neotalem$2 or norexan$2 or novanthron$2 or novantron$2 or novantrone$2 or now-85-34 or now-8534 or now8534 or nsc-287836 or nsc-279836 or nsc-301739 or nsc-301739d or nsc287836 or nsc279836 or nsc301739 or nsc301739d or oncotron$2 or onkotrone$2 or quinizarin$2 or ralenova$2 or pralifan$2 or nsc 279836 or BZ114NVM5P or 65271-80-9 or 70476-82-3 or 137635-96-2 or 70945-62-9 or 70711-41-0).ti,ab,kw. [MITOXANTRONE TERMS] | 33163 |
| 116 | (pembrolizumab$2 or keytruda$2 or lambrolizumab$2 or mk-3475 or mk3475 or sch-900475 or sch900475 or Merck-3475 or DPT0O3T46P or 1374853-91-4).ti,ab,kw. [PEMBROLIZUMAB TERMS] | 20243 |
| 117 | (sipuleucelT$2 or sipuleucel-T or apc-8015 or apc8015 or provenge$2 or 8Q622VDR18 or 917381-47-6).ti,ab,kw. [SIPULEUCEL-T TERMS] | 1999 |
| 118 | (enzalutamide$2 or xtandi$2 or mdv-3100 or mdv3100 or hc-1119 or hc1119 or 915087-33-1 or 93T0T9GKNU).ti,ab,kw. [ENZALUTAMIDE TERMS] | 8467 |
| 119 | (apalutamide$2 or arn-509 or arn509 or erleada or 956104-40-8 or 4T36H88UA7).ti,ab,kw. [APALUTAMIDE TERMS] | 1084 |
| 120 | (darolutamide$2 or bay-1841788 or bay1841788 or nubeqa or odm201 or orm-16497 or odm-201 or orm-16555 or 1297538-32-9 or X05U0N2RCO).ti,ab,kw. [DAROLUTAMIDE TERMS] | 535 |
| 121 | Abiraterone Acetate/ or (abiraterone$ or abretone$2 or cb-7630 or cb7630 or yonsa$2 or zytiga$2 or nsc-749227 or nsc-748121 or 154229-18-2 or EM5OCB9YJ6).ti,ab,kw. [ABIRATERONE ACETATE TERMS] | 9919 |
| 122 | (cb-07598 or cb7598 or nsc-741232 or 154229-19-3 or G819A456D0).ti,ab,kw. [ABIRATERONE TERMS] | 6 |
| 123 | (alpharadin$2 or bay-88-8223 or bay888223 or bay88-8223 or radium223 or radium-223 or radium-ra-223 or radium-ra223 or radiumra-223 or xofigo$2 or ((Ra-223 or 223Ra) adj radioisotope$) or 8BR2SOL3L1 or 15623-45-7).ti,ab,kw. [RADIUM-223 TERMS] | 2713 |
| 124 | (ipatasertib$2 or gdc-0068 or gdc0068 or rg-7440 or rg7440 or 524Y3IB4HQ or 1001264-89-6).ti,ab,kw. [IPATASERTIB TERMS] | 377 |
| 125 | or/109-124 [ALL DRUGS] | 160385 |
| 126 | 108 and 125 [(m)CRPC - ALL DRUGS] | 16519 |
| 127 | exp Child/ not (exp Adult/ and exp Child/) | 3340023 |
| 128 | exp Infant/ not (exp Adult/ and exp Infant/) | 1703513 |
| 129 | 126 not (127 or 128) [CHILD AND INFANT - ONLY REMOVED] | 16511 |
| 130 | 129 use cctr [CENTRAL records] | 1934 |
| 131 | 45 or 100 or 130 [All Databases] | 6346 |
| 132 | limit 131 to yr="2015 -Current" | 4152 |
| 133 | limit 131 to yr="1860 -2014" | 2192 |
| 134 | remove duplicates from 132 | 3080 |
| 135 | remove duplicates from 133 | 1518 |
| 136 | 134 or 135 | 4598 |
| **137** | **remove duplicates from 136 [All RCTs results - deduplicated]** | **4591** |
| 138 | Prostatic Neoplasms/ or (((prostate or prostatic) adj3 (adenocarcinoma$ or adeno-carcinoma$ or cancer$ or carcinoma$ or malignan$ or neoplas$ or tumor? or tumour?)) or PC or PCa).ti,ab,kf. | 662314 |
| 139 | Orchiectomy/ and (insensitiv$ or refractor$ or resistan$).ti,ab,kf. | 2335 |
| 140 | (((androgen? or castrat$ or hormon$) adj2 (independen$ or insensitiv$ or refractor$ or resistan$)) or ((orchectom$ or orcheotom$ or orchidectom$ or orchiectom$ or testectom$ or (removal adj3 (testicle? or test#s))) and (insensitiv$ or refractor$ or resistan$))).ti,ab,kf. | 64313 |
| 141 | 138 and (139 or 140) | 47982 |
| 142 | Prostatic Neoplasms, Castration-Resistant/ or (MCRPC or CRPC or MCRPCa or CRPCa or (((prostate or prostatic) adj3 (adenocarcinoma$ or adeno-carcinoma$ or cancer$ or carcinoma$ or malignan$ or neoplas$ or tumor? or tumour?)) and ((androgen? or castrat$ or hormon$) adj2 (independen$ or insensitiv$ or refractor$ or resistan$)))).ti,ab,kf. | 50907 |
| 143 | 141 or 142 | 51186 |
| 144 | exp Neoplasm Metastasis/ or (metasta$ or ((disseminat$ or spread$ or secondary or migrat$ or seeding) adj3 (neoplas$ or cancer$ or carcinoma$ or adenocarcinoma$ or adeno-carcinoma$ or tumo?r? or sarcoma)) or micrometasta$ or advanced or incurable or late stage? or lethal$ or noncurable or non-curable or progressive or stage IV or terminal or uncurable).ti,ab,kf. | 4683323 |
| 145 | 143 and 144 [MCRPC] | 34884 |
| 146 | (talazoparib$2 or Bmn-673 or Bmn-673ts or bmn673 or bmn673ts or lt-006673 or lt-673 or lt006673 or lt673 or mdv-3800 or mdv3800 or talzenna$2 or 1207456-01-6 or 1373431-65-2 or 9QHX048FRV).ti,ab,kw,kf,rn. [TALAZOPARIB TERMS] | 1676 |
| 147 | (rucaparib$ or ag-014699 or ag-14447 or ag-14699 or ag014699 or ag14447 or ag14699 or co-338 or co338 or pf-01367338 or pf-1367338 or pf-1367338-bw or pf01367338 or pf1367338 or pf1367338bw or rubraca$2 or 1859053-21-6 or 283173-50-2 or 459868-92-9 or 8237f3u7eh).ti,ab,kw,kf,rn. [RUCAPARIB TERMS] | 2193 |
| 148 | (olaparib$2 or azd-2281 or azd2281 or azd221 or ku-0059436 or ku-59436 or ku0059436 or ku59436 or nsc-747856 or lynparza or 763113-22-0 or WOH1JD9AR8).ti,ab,kw,kf,rn. [OLAPARIB TERMS] | 9318 |
| 149 | (niraparib$ or gsk-3985771 or gsk3985771 or jnj- 64091742 or jnj64091742 or mk-4827 or mk4827 or zejula or zl-2306 or zl2306 or 1038915-60-4 or 1038915-73-9 or 1613220-15-7 or HMC2H89N35).ti,ab,kw,kf,rn. [NIRAPARIB TERMS] | 2069 |
| 150 | Docetaxel/ or (docetaxel$ or daxotel$2 or dexotel$2 or docetaxol$2 or docefrez$2 or lit-976 or lit976 or nsc-628503 or nsc628503 or oncodocel$2 or rp-56976 or rp56976 or taxespira$2 or taxoter$2 or taxotere$2 or taxoltere$2 or texot$2 or taxoel$2 or xrp6976 or hsdb-6965 or sid-530 or 114977-28-5 or 699121PHCA).ti,ab,kw,kf,rn. [DOCETAXEL TERMS] | 91529 |
| 151 | (cabazitaxel$2 or kabazitaxel$2 or jevtana$2 or rpr-116258-a or rpr-116258a or rpr116258a or txd-258 or txd258 or xrp-6258 or xrp6258 or nsc-761432 or 51F690397J or 183133-96-2).ti,ab,kw,kf,rn. [CABAZITAXEL TERMS] | 4823 |
| 152 | Mitoxantrone/ or (mitoxantron$ or dhad or dhaq or domitrone$2 or elsep$2 or formyxan$2 or genefadrone$2 or misostol$2 or mitoxanthrone$2 or mitoxgen$2 or mitozantrone$2 or mitroxantrone$2 or mitroxone$2 or neotalem$2 or norexan$2 or novanthron$2 or novantron$2 or novantrone$2 or now-85-34 or now-8534 or now8534 or nsc-287836 or nsc-279836 or nsc-301739 or nsc-301739d or nsc287836 or nsc279836 or nsc301739 or nsc301739d or oncotron$2 or onkotrone$2 or quinizarin$2 or ralenova$2 or pralifan$2 or nsc 279836 or BZ114NVM5P or 65271-80-9 or 70476-82-3 or 137635-96-2 or 70945-62-9 or 70711-41-0).ti,ab,kw,kf,rn. [MITOXANTRONE TERMS] | 33266 |
| 153 | (pembrolizumab$2 or keytruda$2 or lambrolizumab$2 or mk-3475 or mk3475 or sch-900475 or sch900475 or Merck-3475 or DPT0O3T46P or 1374853-91-4).ti,ab,kw,kf,rn. [PEMBROLIZUMAB TERMS] | 31440 |
| 154 | (sipuleucelT$2 or sipuleucel-T or apc-8015 or apc8015 or provenge$2 or 8Q622VDR18 or 917381-47-6).ti,ab,kw,kf,rn. [SIPULEUCEL-T TERMS] | 3239 |
| 155 | (enzalutamide$2 or xtandi$2 or mdv-3100 or mdv3100 or hc-1119 or hc1119 or 915087-33-1 or 93T0T9GKNU).ti,ab,kw,kf,rn. [ENZALUTAMIDE TERMS] | 11154 |
| 156 | (apalutamide$2 or arn-509 or arn509 or erleada or 956104-40-8 or 4T36H88UA7).ti,ab,kw,kf,rn. [APALUTAMIDE TERMS] | 1468 |
| 157 | (darolutamide$2 or bay-1841788 or bay1841788 or nubeqa or odm201 or orm-16497 or odm-201 or orm-16555 or 1297538-32-9 or X05U0N2RCO).ti,ab,kw,kf,rn. [DAROLUTAMIDE TERMS] | 688 |
| 158 | Abiraterone Acetate/ or (abiraterone$ or abretone$2 or cb-7630 or cb7630 or yonsa$2 or zytiga$2 or nsc-749227 or nsc-748121 or 154229-18-2 or EM5OCB9YJ6).ti,ab,kw,kf,rn. [ABIRATERONE ACETATE TERMS] | 11808 |
| 159 | (cb-07598 or cb7598 or nsc-741232 or 154229-19-3 or G819A456D0).ti,ab,kw,kf,rn. [ABIRATERONE TERMS] | 5163 |
| 160 | (alpharadin$2 or bay-88-8223 or bay888223 or bay88-8223 or radium223 or radium-223 or radium-ra-223 or radium-ra223 or radiumra-223 or xofigo$2 or ((Ra-223 or 223Ra) adj radioisotope$) or 8BR2SOL3L1 or 15623-45-7).ti,ab,kw,kf,rn. [RADIUM-223 TERMS] | 2900 |
| 161 | (ipatasertib$2 or gdc-0068 or gdc0068 or rg-7440 or rg7440 or 524Y3IB4HQ or 1001264-89-6).ti,ab,kw,kf,rn. [IPATASERTIB TERMS] | 595 |
| 162 | or/146-161 [ALL DRUGS] | 174928 |
| 163 | 145 and 162 | 16887 |
| 164 | exp Animals/ not Humans/ | 17509914 |
| 165 | 163 not 164 [ANIMAL-ONLY REMOVED] | 11764 |
| 166 | (comment or editorial or news or newspaper article).pt. | 2237720 |
| 167 | (letter not (letter and randomized controlled trial)).pt. | 2336459 |
| 168 | 165 not (166 or 167) [OPINION PIECES REMOVED] | 11421 |
| 169 | exp Child/ not (exp Adult/ and exp Child/) | 3340023 |
| 170 | exp Infant/ not (exp Adult/ and exp Infant/) | 1703513 |
| 171 | 168 not (169 or 170) [CHILD AND INFANT - ONLY REMOVED] | 11414 |
| 172 | (systematic review or systematic literature review or systematic scoping review or systematic narrative review or systematic qualitative review or systematic evidence review or systematic quantitative review or "systematic meta-review" or systematic critical review or systematic mixed studies review or systematic mapping review or systematic cochrane review or "systematic search and review" or systematic integrative review).ti. not comment.pt. not (protocol or protocols).ti. not MEDLINE.st. | 229565 |
| 173 | (1469-493X or 1361-6137).is. and review.pt. | 31281 |
| 174 | systematic review.pt. | 176236 |
| 175 | 172 or 173 or 174 [Ovid Expert Searches: SLR filter 2019] | 421705 |
| 176 | (meta-analy$ or metanaly$ or metaanaly$ or met-analy$).mp,pt. or review.pt. [SLR & MA - modified; Montori, 2004 - Balanced query, sn>sp Filter ] | 6066260 |
| 177 | Network Meta-Analysis/ or ((network adj (MA or MAs)) or (NMA or NMAs or MTC or MTCs or MAIC or MAICs) or indirect$ compar$ or (indirect treatment$ adj1 compar$) or (mixed treatment$ adj1 compar$) or (multiple treatment$ adj1 compar$) or (multi-treatment$ adj1 compar$) or simultaneous$ compar$ or mixed comparison?).tw,kf. [Additional terms for MA, NMA, ITC] | 35827 |
| 178 | (cochrane or health technology assessment or evidence report or systematic reviews).jw. | 65113 |
| 179 | (systematic overview$ or evidence-based review$ or evidence-based overview$ or (evidence adj3 (review$ or overview$)) or meta-review$ or meta-overview$ or meta-synthes$ or rapid review$ or "review of reviews" or umbrella review? or technology assessment$ or HTA or HTAs).tw,kf. [Additional terms for synonyms for systematic reviews and HTAs based on SLRs] | 168105 |
| 180 | or/172-179 [SLR & MA FILTERS - Combined] | 6232955 |
| 181 | 171 and 180 [(m)CRPC & ALL DRUGS & SLRs] | 2198 |
| 182 | 181 use ppez [MEDLINE RECORDS] | 1197 |
| 183 | exp prostate cancer/ or (((prostate or prostatic) adj3 (adenocarcinoma$ or adeno-carcinoma$ or cancer$ or carcinoma$ or malignan$ or neoplas$ or tumor? or tumour?)) or PC or PCa).ti,ab,kw. | 718119 |
| 184 | orchiectomy/ and (insensitiv$ or refractor$ or resistan$).ti,ab,kw. | 2347 |
| 185 | (((androgen? or castrat$ or hormon$) adj2 (independen$ or insensitiv$ or refractor$ or resistan$)) or ((orchectom$ or orcheotom$ or orchidectom$ or orchiectom$ or testectom$ or (removal adj3 (testicle? or test#s))) and (insensitiv$ or refractor$ or resistan$))).ti,ab,kw. | 64696 |
| 186 | 183 and (184 or 185) | 48224 |
| 187 | castration resistant prostate cancer/ or (MCRPC or CRPC or MCRPCa or CRPCa or (((prostate or prostatic) adj3 (adenocarcinoma$ or adeno-carcinoma$ or cancer$ or carcinoma$ or malignan$ or neoplas$ or tumor? or tumour?)) and ((androgen? or castrat$ or hormon$) adj2 (independen$ or insensitiv$ or refractor$ or resistan$)))).ti,ab,kw. | 51702 |
| 188 | 186 or 187 | 52045 |
| 189 | exp metastasis/ or (metasta$ or ((disseminat$ or spread$ or secondary or migrat$ or seeding) adj3 (neoplas$ or cancer$ or carcinoma$ or adenocarcinoma$ or adeno-carcinoma$ or tumo?r? or sarcoma)) or micrometasta$ or advanced or incurable or late stage? or lethal$ or noncurable or non-curable or progressive or stage IV or terminal or uncurable).ti,ab,kw. | 4693684 |
| 190 | 188 and 189 [MCRPC] | 35425 |
| 191 | talazoparib/ or (talazoparib$2 or Bmn-673 or Bmn-673ts or bmn673 or bmn673ts or lt-006673 or lt-673 or lt006673 or lt673 or mdv-3800 or mdv3800 or talzenna$2 or 1207456-01-6 or 1373431-65-2 or 9QHX048FRV).ti,ab,kw,du,dy,tn,rn. [TALAZOPARIB TERMS] | 1719 |
| 192 | rucaparib/ or (rucaparib$ or ag-014699 or ag-14447 or ag-14699 or ag014699 or ag14447 or ag14699 or co-338 or co338 or pf-01367338 or pf-1367338 or pf-1367338-bw or pf01367338 or pf1367338 or pf1367338bw or rubraca$2 or 1859053-21-6 or 283173-50-2 or 459868-92-9 or 8237f3u7eh).ti,ab,kw,du,dy,tn,rn. [RUCAPARIB TERMS] | 2327 |
| 193 | olaparib/ or (olaparib$2 or azd-2281 or azd2281 or azd221 or ku-0059436 or ku-59436 or ku0059436 or ku59436 or nsc-747856 or lynparza or 763113-22-0 or WOH1JD9AR8).ti,ab,kw,du,dy,tn,rn. [OLAPARIB TERMS] | 9388 |
| 194 | niraparib/ or (niraparib$ or gsk-3985771 or gsk3985771 or jnj- 64091742 or jnj64091742 or mk-4827 or mk4827 or zejula or zl-2306 or zl2306 or 1038915-60-4 or 1038915-73-9 or 1613220-15-7 or HMC2H89N35).ti,ab,kw,du,dy,tn,rn. [NIRAPARIB TERMS] | 2134 |
| 195 | docetaxel/ or (docetaxel$ or daxotel$2 or dexotel$2 or docetaxol$2 or docefrez$2 or lit-976 or lit976 or nsc-628503 or nsc628503 or oncodocel$2 or rp-56976 or rp56976 or taxespira$2 or taxoter$2 or taxotere$2 or taxoltere$2 or texot$2 or taxoel$2 or xrp6976 or hsdb-6965 or sid-530 or 114977-28-5 or 699121PHCA).ti,ab,kw,du,dy,tn,rn. [DOCETAXEL TERMS] | 91529 |
| 196 | cabazitaxel/ or (cabazitaxel$2 or kabazitaxel$2 or jevtana$2 or rpr-116258-a or rpr-116258a or rpr116258a or txd-258 or txd258 or xrp-6258 or xrp6258 or nsc-761432 or 51F690397J or 183133-96-2).ti,ab,kw,du,dy,tn,rn. [CABAZITAXEL TERMS] | 4858 |
| 197 | mitoxantrone/ or (mitoxantron$ or dhad or dhaq or domitrone$2 or elsep$2 or formyxan$2 or genefadrone$2 or misostol$2 or mitoxanthrone$2 or mitoxgen$2 or mitozantrone$2 or mitroxantrone$2 or mitroxone$2 or neotalem$2 or norexan$2 or novanthron$2 or novantron$2 or novantrone$2 or now-85-34 or now-8534 or now8534 or nsc-287836 or nsc-279836 or nsc-301739 or nsc-301739d or nsc287836 or nsc279836 or nsc301739 or nsc301739d or oncotron$2 or onkotrone$2 or quinizarin$2 or ralenova$2 or pralifan$2 or nsc 279836 or BZ114NVM5P or 65271-80-9 or 70476-82-3 or 137635-96-2 or 70945-62-9 or 70711-41-0).ti,ab,kw,du,dy,tn,rn. [MITOXANTRONE TERMS] | 33269 |
| 198 | pembrolizumab/ or (pembrolizumab$2 or keytruda$2 or lambrolizumab$2 or mk-3475 or mk3475 or sch-900475 or sch900475 or Merck-3475 or DPT0O3T46P or 1374853-91-4).ti,ab,kw,du,dy,tn,rn. [PEMBROLIZUMAB TERMS] | 31504 |
| 199 | sipuleucel t/ or (sipuleucelT$2 or sipuleucel-T or apc-8015 or apc8015 or provenge$2 or 8Q622VDR18 or 917381-47-6).ti,ab,kw,du,dy,tn,rn. [SIPULEUCEL-T TERMS] | 3692 |
| 200 | enzalutamide/ or (enzalutamide$2 or xtandi$2 or mdv-3100 or mdv3100 or hc-1119 or hc1119 or 915087-33-1 or 93T0T9GKNU).ti,ab,kw,du,dy,tn,rn. [ENZALUTAMIDE TERMS] | 11278 |
| 201 | apalutamide/ or (apalutamide$2 or arn-509 or arn509 or erleada or 956104-40-8 or 4T36H88UA7).ti,ab,kw,du,dy,tn,rn. [APALUTAMIDE TERMS] | 1535 |
| 202 | darolutamide/ or (darolutamide$2 or bay-1841788 or bay1841788 or nubeqa or odm201 or orm-16497 or odm-201 or orm-16555 or 1297538-32-9 or X05U0N2RCO).ti,ab,kw,du,dy,tn,rn. [DAROLUTAMIDE TERMS] | 722 |
| 203 | abiraterone acetate/ or (abiraterone$ or abretone$2 or cb-7630 or cb7630 or yonsa$2 or zytiga$2 or nsc-749227 or nsc-748121 or 154229-18-2 or EM5OCB9YJ6).ti,ab,kw,du,dy,tn,rn. [ABIRATERONE ACETATE TERMS] | 11817 |
| 204 | abiraterone/ or (abiraterone$2 or cb-07598 or cb7598 or nsc-741232 or 154229-19-3 or G819A456D0).ti,ab,kw,du,dy,tn,rn. [ABIRATERONE TERMS] | 11786 |
| 205 | "radium chloride ra 223"/ or (alpharadin$2 or bay-88-8223 or bay888223 or bay88-8223 or radium223 or radium-223 or radium-ra-223 or radium-ra223 or radiumra-223 or xofigo$2 or ((Ra-223 or 223Ra) adj radioisotope$) or 8BR2SOL3L1 or 15623-45-7).ti,ab,kw,du,dy,tn,rn. [RADIUM-223 TERMS] | 3503 |
| 206 | ipatasertib/ or (ipatasertib$2 or gdc-0068 or gdc0068 or rg-7440 or rg7440 or 524Y3IB4HQ or 1001264-89-6).ti,ab,kw,du,dy,tn,rn. [IPATASERTIB TERMS] | 624 |
| 207 | or/191-206 [ALL DRUGS] | 175610 |
| 208 | 190 and 207 | 17327 |
| 209 | exp animal/ or exp animal experimentation/ or exp animal model/ or exp animal experiment/ or nonhuman/ or exp vertebrate/ | 54609070 |
| 210 | exp human/ or exp human experimentation/ or exp human experiment/ | 42976108 |
| 211 | 209 not 210 | 11634698 |
| 212 | 208 not 211 [ANIMAL-ONLY REMOVED] | 17008 |
| 213 | editorial.pt. | 1281683 |
| 214 | letter.pt. not (letter.pt. and randomized controlled trial/) | 2336385 |
| 215 | 212 not (213 or 214) [OPINION PIECES REMOVED] | 16621 |
| 216 | exp adolescent/ not (exp adult/ and exp adolescent/) | 1263029 |
| 217 | exp child/ not (exp adult/ and exp child/) | 3340023 |
| 218 | fetus/ not (fetus/ and exp adult/) | 231241 |
| 219 | 215 not (216 or 217 or 218) [UNDER 18 REMOVED] | 16612 |
| 220 | exp Meta Analysis/ or ((meta adj analy$) or metaanalys$).mp. or (systematic adj (review$1 or overview$1)).tw. or (cancerlit or cochrane or embase or psychlit or psyclit or psychinfo or psycinfo or cinahl or cinhal or science citation index or bids or reference lists or bibliograph$ or hand-search$ or manual search$ or relevant journals).ab. | 1018989 |
| 221 | (data extraction or selection criteria).ab. and review.pt. | 62930 |
| 222 | 220 or 221 [SLR & MA FILTER - Ovid Expert Searches: SLR filter 2019] | 1029334 |
| 223 | (meta-analy$ or metanaly$ or metaanaly$ or met-analy$).mp. or review.pt. [SLR & MA FILTER - modified and translated; Montori, 2004 - Balanced query, sn>sp Filter ] | 6066055 |
| 224 | Network Meta-Analysis/ or ((network adj (MA or MAs)) or (NMA or NMAs or MTC or MTCs or MAIC or MAICs) or indirect$ compar$ or (indirect treatment$ adj1 compar$) or (mixed treatment$ adj1 compar$) or (multiple treatment$ adj1 compar$) or (multi-treatment$ adj1 compar$) or simultaneous$ compar$ or mixed comparison?).tw,kw. [Additional terms for MA, NMA, ITC] | 35927 |
| 225 | (cochrane or health technology assessment or evidence report or systematic reviews).jw. | 65113 |
| 226 | (systematic overview$ or evidence-based review$ or evidence-based overview$ or (evidence adj3 (review$ or overview$)) or meta-review$ or meta-overview$ or meta-synthes$ or rapid review$ or "review of reviews" or umbrella review? or technology assessment$ or HTA or HTAs).tw,kw. [Additional terms for synonyms for systematic reviews and HTAs based on SLRs] | 169232 |
| 227 | or/220-226 [SLR & MA FILTERS - Combined] | 6347932 |
| 228 | 219 and 227 [(m)CRPC & ALL DRUGS & SLRs] | 3338 |
| 229 | conference abstract.pt. | 4191531 |
| 230 | 228 not 229 [CONFERENCE ABSTRACTS REMOVED] | 3210 |
| 231 | 228 and 229 | 128 |
| 232 | limit 231 to yr="2019-current" | 45 |
| 233 | 230 or 232 [MOST RECENT 2 YEARS CONF ABSTRACTS RETAINED] | 3255 |
| 234 | 233 use oemezd [EMBASE RECORDS] | 2047 |
| 235 | (((prostate or prostatic) adj3 (adenocarcinoma$ or adeno-carcinoma$ or cancer$ or carcinoma$ or malignan$ or neoplas$ or tumor? or tumour?)) or PC or PCa).ti,ab,kw. | 646283 |
| 236 | (((androgen? or castrat$ or hormon$) adj2 (independen$ or insensitiv$ or refractor$ or resistan$)) or ((orchectom$ or orcheotom$ or orchidectom$ or orchiectom$ or testectom$ or (removal adj3 (testicle? or test#s))) and (insensitiv$ or refractor$ or resistan$))).ti,ab,kw. | 64696 |
| 237 | 235 and 236 | 48025 |
| 238 | (MCRPC or CRPC or MCRPCa or CRPCa or (((prostate or prostatic) adj3 (adenocarcinoma$ or adeno-carcinoma$ or cancer$ or carcinoma$ or malignan$ or neoplas$ or tumor? or tumour?)) and ((androgen? or castrat$ or hormon$) adj2 (independen$ or insensitiv$ or refractor$ or resistan$)))).ti,ab,kw. | 49195 |
| 239 | 237 or 238 | 49349 |
| 240 | (metasta$ or ((disseminat$ or spread$ or secondary or migrat$ or seeding) adj3 (neoplas$ or cancer$ or carcinoma$ or adenocarcinoma$ or adeno-carcinoma$ or tumo?r? or sarcoma)) or micrometasta$ or advanced or incurable or late stage? or lethal$ or noncurable or non-curable or progressive or stage IV or terminal or uncurable).ti,ab,kw. | 4495102 |
| 241 | 239 and 240 [MCRPC] | 33495 |
| 242 | (talazoparib$2 or Bmn-673 or Bmn-673ts or bmn673 or bmn673ts or lt-006673 or lt-673 or lt006673 or lt673 or mdv-3800 or mdv3800 or talzenna$2 or 1207456-01-6 or 1373431-65-2 or 9QHX048FRV).ti,ab,kw. [TALAZOPARIB TERMS] | 1041 |
| 243 | (rucaparib$ or ag-014699 or ag-14447 or ag-14699 or ag014699 or ag14447 or ag14699 or co-338 or co338 or pf-01367338 or pf-1367338 or pf-1367338-bw or pf01367338 or pf1367338 or pf1367338bw or rubraca$2 or 1859053-21-6 or 283173-50-2 or 459868-92-9 or 8237f3u7eh).ti,ab,kw. [RUCAPARIB TERMS] | 1276 |
| 244 | (olaparib$2 or azd-2281 or azd2281 or azd221 or ku-0059436 or ku-59436 or ku0059436 or ku59436 or nsc-747856 or lynparza or 763113-22-0 or WOH1JD9AR8).ti,ab,kw. [OLAPARIB TERMS] | 6195 |
| 245 | (niraparib$ or gsk-3985771 or gsk3985771 or jnj- 64091742 or jnj64091742 or mk-4827 or mk4827 or zejula or zl-2306 or zl2306 or 1038915-60-4 or 1038915-73-9 or 1613220-15-7 or HMC2H89N35).ti,ab,kw. [NIRAPARIB TERMS] | 1235 |
| 246 | (docetaxel$ or daxotel$2 or dexotel$2 or docetaxol$2 or docefrez$2 or lit-976 or lit976 or nsc-628503 or nsc628503 or oncodocel$2 or rp-56976 or rp56976 or taxespira$2 or taxoter$2 or taxotere$2 or taxoltere$2 or texot$2 or taxoel$2 or xrp6976 or hsdb-6965 or sid-530 or 114977-28-5 or 699121PHCA).ti,ab,kw. [DOCETAXEL TERMS] | 54496 |
| 247 | (cabazitaxel$2 or kabazitaxel$2 or jevtana$2 or rpr-116258-a or rpr-116258a or rpr116258a or txd-258 or txd258 or xrp-6258 or xrp6258 or nsc-761432 or 51F690397J or 183133-96-2).ti,ab,kw. [CABAZITAXEL TERMS] | 3406 |
| 248 | (mitoxantron$ or dhad or dhaq or domitrone$2 or elsep$2 or formyxan$2 or genefadrone$2 or misostol$2 or mitoxanthrone$2 or mitoxgen$2 or mitozantrone$2 or mitroxantrone$2 or mitroxone$2 or neotalem$2 or norexan$2 or novanthron$2 or novantron$2 or novantrone$2 or now-85-34 or now-8534 or now8534 or nsc-287836 or nsc-279836 or nsc-301739 or nsc-301739d or nsc287836 or nsc279836 or nsc301739 or nsc301739d or oncotron$2 or onkotrone$2 or quinizarin$2 or ralenova$2 or pralifan$2 or nsc 279836 or BZ114NVM5P or 65271-80-9 or 70476-82-3 or 137635-96-2 or 70945-62-9 or 70711-41-0).ti,ab,kw. [MITOXANTRONE TERMS] | 15250 |
| 249 | (pembrolizumab$2 or keytruda$2 or lambrolizumab$2 or mk-3475 or mk3475 or sch-900475 or sch900475 or Merck-3475 or DPT0O3T46P or 1374853-91-4).ti,ab,kw. [PEMBROLIZUMAB TERMS] | 20243 |
| 250 | (sipuleucelT$2 or sipuleucel-T or apc-8015 or apc8015 or provenge$2 or 8Q622VDR18 or 917381-47-6).ti,ab,kw. [SIPULEUCEL-T TERMS] | 1999 |
| 251 | (enzalutamide$2 or xtandi$2 or mdv-3100 or mdv3100 or hc-1119 or hc1119 or 915087-33-1 or 93T0T9GKNU).ti,ab,kw. [ENZALUTAMIDE TERMS] | 8467 |
| 252 | (abiraterone$ or abretone$2 or cb-7630 or cb7630 or yonsa$2 or zytiga$2 or nsc-749227 or nsc-748121 or 154229-18-2 or EM5OCB9YJ6).ti,ab,kw. [ABIRATERONE ACETATE TERMS] | 8856 |
| 253 | (enzalutamide$2 or xtandi$2 or mdv-3100 or mdv3100 or hc-1119 or hc1119 or 915087-33-1 or 93T0T9GKNU).ti,ab,kw. [ENZALUTAMIDE TERMS] | 8467 |
| 254 | (apalutamide$2 or arn-509 or arn509 or erleada or 956104-40-8 or 4T36H88UA7).ti,ab,kw. [APALUTAMIDE TERMS] | 1084 |
| 255 | (darolutamide$2 or bay-1841788 or bay1841788 or nubeqa or odm201 or orm-16497 or odm-201 or orm-16555 or 1297538-32-9 or X05U0N2RCO).ti,ab,kw. [DAROLUTAMIDE TERMS] | 535 |
| 256 | (abiraterone$ or abretone$2 or cb-7630 or cb7630 or yonsa$2 or zytiga$2 or nsc-749227 or nsc-748121 or 154229-18-2 or EM5OCB9YJ6).ti,ab,kw. [ABIRATERONE ACETATE TERMS] | 8856 |
| 257 | (cb-07598 or cb7598 or nsc-741232 or 154229-19-3 or G819A456D0).ti,ab,kw. [ABIRATERONE TERMS] | 6 |
| 258 | (alpharadin$2 or bay-88-8223 or bay888223 or bay88-8223 or radium223 or radium-223 or radium-ra-223 or radium-ra223 or radiumra-223 or xofigo$2 or ((Ra-223 or 223Ra) adj radioisotope$) or 8BR2SOL3L1 or 15623-45-7).ti,ab,kw. [RADIUM-223 TERMS] | 2713 |
| 259 | (ipatasertib$2 or gdc-0068 or gdc0068 or rg-7440 or rg7440 or 524Y3IB4HQ or 1001264-89-6).ti,ab,kw. [IPATASERTIB TERMS] | 377 |
| 260 | or/242-259 [ALL DRUGS] | 108606 |
| 261 | 241 and 260 [(m)CRPC - ALL DRUGS] | 14942 |
| 262 | 261 use coch [CDSR RECORDS] | 5 |
| 263 | 182 or 234 or 262 | 3249 |
| **264** | **remove duplicates from 263 [All SLRs and NMAs results - deduplicated]** | **2368** |
| 265 | 137 or 264 [All results – RCTs, SLRs and NMAs] | 6174 |
| 266 | limit 265 to yr="2015 -Current" | 3871 |
| 267 | limit 265 to yr="1860 -2014" | 2302 |
| 268 | remove duplicates from 266 | 3783 |
| 269 | remove duplicates from 267 | 2151 |
| 270 | 268 or 269 | 5934 |
| **271** | **remove duplicates from 270 [All results – RCTs, SLRs and NMAs; deduplicated]** | **5933** |

PICOS

Table 2: PICOS Criteria

|  | **Inclusion Criteria** | **Exclusion Criteria** |
| --- | --- | --- |
| ***Population*** | Study populations or subgroups of patients (humans only; men) with:   - Age ≥18 years - Histologically or cytologically confirmed adenocarcinoma of the prostate - Undergone surgical or medical castration - Metastatic disease - Castration-resistant/Hormone-resistant/Hormone-refractory/Androgen-independent prostate cancer - Asymptomatic or mildly symptomatic^a^ - Treatment naïve in the mCRPC setting | Study populations or subgroups:   - Non-human - Age <18 years - No surgical castration or medical castration - Non-metastatic disease - Non-CRPC - Hormone-sensitive disease - Any previous systemic cancer treatment for mCRPC disease state^b^ |
| ***Interventions*** | - Treatments under investigation for mCRPC provided as a single-agent or a combination treatment - PARP inhibitors (talazoparib, rucaparib, olaparib, niraparib) - Chemotherapy agents   - Taxanes (docetaxel, cabazitaxel)   - Mitoxantrone - Immunotherapies (pembrolizumab, sipuleucel-T) - Novel hormone therapies   - - Direct androgen receptor inhibitors (enzalutamide, apalutamide, darolutamide)     - Abiraterone acetate - Radiotherapy (radium-223) - AKT inhibitor (ipatasertib) | Those not listed in *Inclusion Criteria* |
| ***Comparators*** | - Treatments under investigation for mCRPC provided as a single-agent or combination treatment - PARP inhibitors (talazoparib, rucaparib, olaparib, niraparib) - Chemotherapy agents   - Taxanes (docetaxel, cabazitaxel)   - Mitoxantrone - Immunotherapies (pembrolizumab, sipuleucel-T) - NHTs   - - Direct AR inhibitors (enzalutamide, apalutamide, darolutamide)     - Abiraterone acetate - Radiotherapy (radium-223) - AKT inhibitor (ipatasertib) - BSC or placebo or watchful waiting | Those not listed in *Inclusion Criteria* |
| ***Outcomes*** | - Survival endpoints:   - OS   - PFS   - rPFS   - PFS2 - Response endpoints:   - ORR   - DoR   - PSA response   - Proportion of patients with PSA response ≥50% - Other endpoints:   - Time to PSA progression   - Time to initiation of cytotoxic chemotherapy   - Time to initiation of antineoplastic therapy   - Time to first symptomatic skeletal event   - Opioid use for cancer pain - Safety endpoints:   - Incidence of AEs   - Serious AEs   - AEs leading to discontinuation - Patient-reported outcomes:   - HRQoL (eg, EQ-5D, FACT-P, SF-36, EORTC QLQ-C30, EORTC QLQ-PR25, BPI-SF, PGI-S, etc.) | Those not listed in *Inclusion Criteria* |
| ***Study Design*** | - RCTs irrespective of blinding status^c^   - Phase II, II/III, III - Conference abstracts of RCTs | - Non-RCTs (ie, single-arm clinical trials and observational studies) - Phase I, I/II, IV trials - Open-label extension studies - Pre-clinical studies - Pharmacokinetic studies - Retrospective studies - Case study, series, or reports - Expert opinion articles - Letters - Editorials - Narrative (non-systematic) reviews - Pilot studies - Protocols - SLRs/MAs/NMAs of RCTs^d^ - Non-clinical studies   - Utilities outcomes only   - Economic outcomes only |
| ***Language^e^*** | - Articles in English | All non-English articles |
| ***Dates*** | Databases: inception-present  Conference abstracts: 2019-present | Databases: none  Conference abstracts: prior to 2019 |

^a^ The definition of symptomatic disease will not be restricted, and definitions based on pain and/or other symptoms will be included. Differing definitions will be evaluated at the feasibility assessment stage.

^b^ Androgen deprivation therapy is not exclusionary.

^c^ Crossover trials will only be included if the randomization portion of the trial has relevant data reported.

^d^ Relevant SLRs/MAs/NMAs will not be included in the final included studies list; however, their bibliographies will be reviewed for any additional relevant studies.

^e^ Search strategy will not be limited by language; however, non-English articles will be excluded during the screening phase.

Abbreviations: AEs = adverse events; AKT = protein kinase B; AR = androgen receptor; BPI-SF = Brief Pain Inventory (Short Form); BSC = best supportive care; CRPC = castration-resistant prostate cancer; DoR = duration of response; EORTC QLQ-C30 = European Organisation for Research and Treatment of Cancer Quality of Life of Cancer Patients Questionnaire; EORTC QLQ-PR25 = European Organisation for Research and Treatment of Cancer Quality of Life Questionnaire - Prostate Cancer Module; EQ-5D = EuroQuol 5-Dimension; FACT-P = Functional Assessment of Cancer Therapy-Prostate; HRQoL = health-related quality of life; MA = meta-analysis; mCRPC = metastatic castration-resistant prostate cancer; NMA = network meta-analysis; ORR = objective response rate; OS = overall survival; PARP = poly (ADP-ribose) polymerase; PFS = progression-free survival; PFS2 = progression free survival on next line of therapy; PGI-S = Patient Global Impression of Severity; PSA = prostate-specific antigen; RCT = randomized controlled trial; rPFS = radiographic progression-free survival; SF-36 = 36-Item Short-Form Survey; SLR = systematic literature review.

Sources Searched

Figure 1: Overview of Sources Searched for the SLR


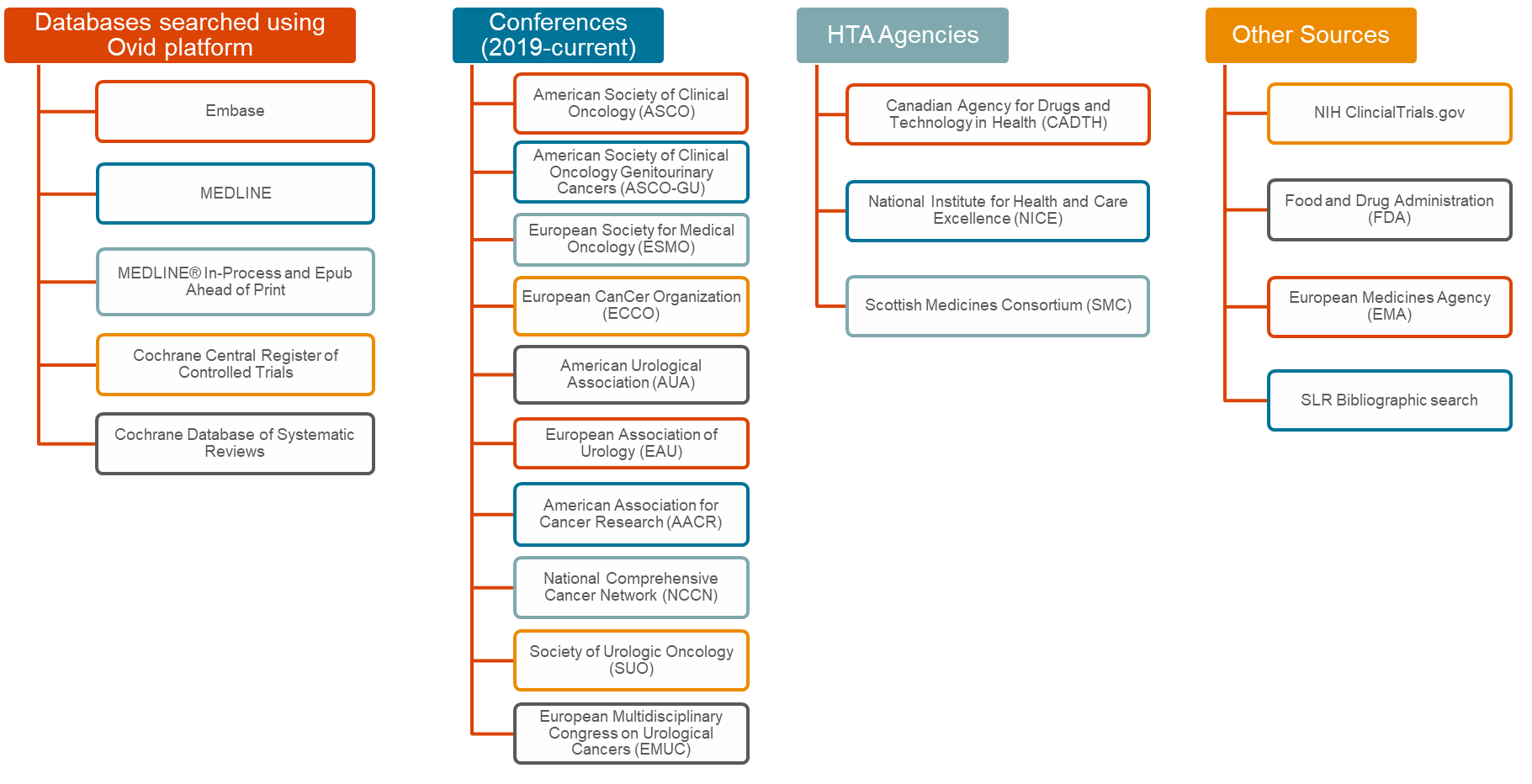


Abbreviations: AACR = American Association for Cancer Research; ASCO = American Society of Clinical Oncology; ASCO-GU = American Society of Clinical Oncology Genitourinary Cancers; AUA = American Urological Association; CADTH = Canadian Agency for Drugs and Technology in Health; EAU = European Association of Urology; EMA = European Medicines Agency; EMUC = European Multidisciplinary Congress on Urologic Cancers; ESMO = European Society for Medical Oncology; FDA = Food and Drug Administration; HTA = health technology assessment; NCCN = National Comprehensive Cancer Network; NICE = National Institute for Health and Care Excellence; NIH = National Institutes of Health; SMC = Scottish Medicines Consortium; SLR = systematic literature review; SUO = Society of Urologic Oncology.

Excluded Studies

Table 3: List of Excluded Studies with Reasons for Exclusion

| **Bibliography** | **What is the reason for exclusion?** |
| --- | --- |
| Wang et al. Comparing the clinical efficacy and safety of abiraterone and enzalutamide in metastatic castration-resistant prostate cancer: A systematic review and meta-analysis. Journal of Oncology Pharmacy Practice. 2021 | On topic SLR/MA/NMA |
| Van Der Zande et al. First results from a randomized phase II study of cabazitaxel (CBZ) versus an androgen receptor targeted agent (ARTA) in patients with poor-prognosis castrationresistant prostate cancer (mCRPC). Journal of Clinical Oncology. 2021 | Population |
| Ternov et al. Quality of life in men with metastatic castration-resistant prostate cancer treated with enzalutamide or abiraterone: a systematic review and meta-analysis. Prostate Cancer and Prostatic Diseases. 2021 | On topic SLR/MA/NMA |
| Solipuram et al. Effect of immunotherapy on survival outcomes in prostate cancer: Systematic review and meta-analysis. Journal of Clinical Oncology. 2021 | On topic SLR/MA/NMA |
| Sepe et al. A phase II study evaluating the efficacy of enzalutamide and the role of ARv7 in metastatic castration-resistant prostate cancer (mCRPC) patients (pts) with visceral disease. Journal of Clinical Oncology. 2021 | Study design (non-RCT, opinion, commentary, single-arm, etc.) |
| Rathkopf et al. Final results from ACIS, a randomized, placebo (PBO)- controlled double-blind phase 3 study of apalutamide (APA) and abiraterone acetate plus prednisone (AAP) versus AAP in patients (pts) with chemo-naive metastatic castration-resistant prostate cancer (mCRPC). Journal of clinical oncology. 2021 | Duplicate |
| Maughan et al. Radium-223 plus Enzalutamide versus Enzalutamide in Metastatic Castration-Refractory Prostate Cancer: Final Safety and Efficacy Results. The oncologist. 2021 | Population |
| Marshall et al. Randomized phase II trial of sipuleucel-T with or without radium-223 in men with bone-metastatic castration-resistant prostate cancer. Clinical Cancer Research. 2021 | Mixed population |
| Lee et al. Abiraterone and enzalutamide had different adverse effects on the cardiovascular system: a systematic review with pairwise and network meta-analyses. Prostate Cancer and Prostatic Diseases. 2021 | On topic SLR/MA/NMA |
| Choudhury et al. Randomized phase II study evaluating the addition of pembrolizumab to radium-223 in metastatic castration-resistant prostate cancer. Journal of Clinical Oncology. 2021 | Incomplete/Insufficient/Partial data |
| Cassinello et al. Optimal treatment sequencing of abiraterone acetate plus prednisone and enzalutamide in patients with castration-resistant metastatic prostate cancer: A systematic review and meta-analysis. Cancer Treatment Reviews. 2021 | Study design (non-RCT, opinion, commentary, single-arm, etc.) |
| Caffo et al. Docetaxel and prednisone with or without enzalutamide as first-line treatment in patients with metastatic castration-resistant prostate cancer: CHEIRON, a randomised phase II trial. European Journal of Cancer. 2021 | Mixed population |
| Batra et al. Cognition and depression effects of androgen receptor axis-targeted drugs in men with prostate cancer: A systematic review. Journal of Geriatric Oncology. 2021 | On topic SLR/MA/NMA |
| Bastos et al. Abiraterone acetate in patients with metastatic castrationresistant prostate cancer, chemonaive, who received a prior diethylstilbestrol therapy. Journal of Clinical Oncology. 2021 | Study design (non-RCT, opinion, commentary, single-arm, etc.) |
| Wang et al. Comparison of effectiveness and safety outcomes of abiraterone versus enzalutamide in patients with metastatic castration-resistant prostate cancer: a systematic review and meta-analysis. Journal of pharmacy & pharmaceutical sciences : a publication of the Canadian Society for Pharmaceutical Sciences, Societe canadienne des sciences pharmaceutiques. 2020 | On topic SLR/MA/NMA |
| Vignani et al. Evaluation of cognitive function (Cogf) in randomized trials testing new-generation hormonal treatments (Nght) in patients with prostate cancer (Pc): A systematic review. Tumori. 2020 | On topic SLR/MA/NMA |
| Ternov et al. Short term health-related quality-of-life in men with metastatic castration-resistant prostate cancer treated with first-line enzalutamide or abiraterone plus prednisone: A systematic review and meta-analysis. European Urology Open Science. 2020 | On topic SLR/MA/NMA |
| Tan et al. The efficacy and safety of abiraterone acetate in patients with high-risk prostate cancer: A meta-analysis based on six randomized control trials. Translational Andrology and Urology. 2020 | On topic SLR/MA/NMA |
| Sternberg et al. A randomised phase II trial of three dosing regimens of radium-223 in patients with bone metastatic castration-resistant prostate cancer. Annals of Oncology. 2020 | Population |
| Slovin et al. Abiraterone acetate (AA) with or without cabazitaxel (CBZ) in treatment of chemotherapy naive metastatic castration-resistant prostate cancer (mCRPC). Journal of clinical oncology. 2020 | Duplicate |
| Sidaway. Sequence of AR inhibitors affects outcome. Nature Reviews Clinical Oncology. 2020 | Study design (non-RCT, opinion, commentary, single-arm, etc.) |
| Ratta et al. PARP inhibitors as a new therapeutic option in metastatic prostate cancer: a systematic review. Prostate Cancer and Prostatic Diseases. 2020 | On topic SLR/MA/NMA |
| Pereira-Salgado et al. Systematic Review of Efficacy and Health Economic Implications of Real-world Treatment Sequencing in Prostate Cancer: Where Do the Newer Agents Enzalutamide and Abiraterone Fit in?. European Urology Focus. 2020 | On topic SLR/MA/NMA |
| Paredero Perez et al. Quality of life and survival in elderly metastatic castration-resistant prostate cancer (mCRPC) patients (Pts) treated with docetaxel. Annals of Oncology. 2020 | Study design (non-RCT, opinion, commentary, single-arm, etc.) |
| Morris et al. A phase III trial of docetaxel versus docetaxel and radium-223 (Ra-223) in patients with metastatic castration-resistant prostate cancer (mCRPC): DORA. Journal of clinical oncology. 2020 | Study design (non-RCT, opinion, commentary, single-arm, etc.) |
| Miller. Sequential therapy for asymptomatic or mildly symptomatic metastatic castration-resistant prostate cancer. Sequenztherapie beim nicht oder mild symptomatischen metastasierten kastrationsresistenten Prostatakarzinom. 2020 | Non-English |
| McCready et al. Radium-223 dichloride in prostate cancer: proof of principle for the use of targeted alpha treatment in clinical practice. European Journal of Nuclear Medicine and Molecular Imaging. 2020 | On topic SLR/MA/NMA |
| Maughan et al. Randomized phase II trial of radium-223 (RA) plus enzalutamide (EZ) versus EZ alone in metastatic castration-refractory prostate cancer (mCRPC): long-term follow up of secondary endpoints. Journal of clinical oncology. 2020 | Duplicate |
| Maughan et al. Randomized phase II trial of radium-223 (RA) plus enzalutamide (EZ) versus EZ alone in metastatic castration-refractory prostate cancer (mCRPC): Long-term follow up of secondary endpoints. Journal of Clinical Oncology. 2020 | Population |
| Matsubara et al. A randomized, double-blind, comparison of radium-223 and placebo, in combination with abiraterone acetate and prednisolone, in castration-resistant metastatic prostate cancer: subgroup analysis of Japanese patients in the ERA 223 study. International Journal of Clinical Oncology. 2020 | Mixed population |
| Marshall et al. Results of the randomized phase II study of sipuleucel-T (Sip-T) +/-Radium-223 (Ra-223) in men with bone-metastatic castration resistant prostate cancer. Journal of clinical oncology. 2020 | Mixed population |
| Marshall et al. Randomized phase II study of sipuleucel-T (SipT) with or without radium-223 (Ra223) in men with asymptomatic bone-metastatic castrate-resistant prostate cancer (mCRPC). Journal of Clinical Oncology. 2020 | Mixed population |
| Marandino et al. Evaluation of cognitive function (CogF) in trials testing new-generation hormonal treatments (NGHT) in patients with prostate cancer (PC): A systematic review. Annals of Oncology. 2020 | On topic SLR/MA/NMA |
| Liao et al. CYP17 inhibitors improve the prognosis of metastatic castration-resistant prostate cancer patients: A meta-analysis of published trials. Journal of Cancer Research and Therapeutics. 2020 | On topic SLR/MA/NMA |
| Kretschmer et al. Health-related Quality of Life in Patients with Advanced Prostate Cancer: A Systematic Review. European Urology Focus. 2020 | On topic SLR/MA/NMA |
| Hijab et al. Fracture risk in men with metastatic castration-resistant prostate cancer (mCRPC) treated with radium-223 (Ra 223). Annals of Oncology. 2020 | Mixed population |
| Chen et al. Longitudinal model-based meta-analysis for survival probabilities in patients with castration-resistant prostate cancer. European Journal of Clinical Pharmacology. 2020 | On topic SLR/MA/NMA |
| Balakrishnar et al. Systematic review and meta-analysis of treatment related toxicities from second-generation androgen receptor inhibitors in advanced prostate cancer. Asia-Pacific Journal of Clinical Oncology. 2020 | On topic SLR/MA/NMA |
| Armstrong et al. Five-year Survival Prediction and Safety Outcomes with Enzalutamide in Men with Chemotherapy-naive Metastatic Castration-resistant Prostate Cancer from the PREVAIL Trial. European Urology. 2020 | Study design (non-RCT, opinion, commentary, single-arm, etc.) |
| Antonarakis et al. When and How to Use PARP Inhibitors in Prostate Cancer: A Systematic Review of the Literature with an Update on On-Going Trials. European Urology Oncology. 2020 | On topic SLR/MA/NMA |
| Uemura et al. Three-year follow-up of a phase II study of radium-223 dichloride in Japanese patients with symptomatic castration-resistant prostate cancer and bone metastases. International Journal of Clinical Oncology. 2019 | Population |
| Teply et al. Risk of development of visceral metastases subsequent to abiraterone vs placebo: An analysis of mode of radiographic progression in COU-AA-302. Prostate. 2019 | Outcome |
| Petrylak et al. A phase IIa study of radium-223 dichloride (Ra-223) alone or in combination with abiraterone acetate or enzalutamide in metastatic castration-resistant prostate cancer (mCRPC). Annals of Oncology. 2019 | Mixed population |
| Morris et al. Alliance A031201: a phase III trial of enzalutamide (ENZ) versus enzalutamide, abiraterone, and prednisone (ENZ/AAP) for metastatic castration resistant prostate cancer (mCRPC). Journal of clinical oncology. 2019 | Duplicate |
| Morris et al. A phase III trial of docetaxel versus docetaxel and radium-223 (Ra-223) in patients with metastatic castration-resistant prostate cancer (mCRPC): DORA. Journal of clinical oncology. 2019 | Study design (non-RCT, opinion, commentary, single-arm, etc.) |
| Kyriakopoulos et al. Cabazitaxel with abiraterone versus abiraterone alone randomized trial for extensive disease following docetaxel: the CHAARTED 2 Trial: a trial of the ECOG-ACRIN Cancer Research Group (EA8153). Journal of clinical oncology. 2019 | Duplicate |
| Kyriakopoulos et al. Cabazitaxel with abiraterone versus abiraterone alone randomized trial for extensive disease following docetaxel: the CHAARTED 2 Trial: A trial of the ECOG-ACRIN Cancer Research Group (EA8153). Journal of Clinical Oncology. 2019 | Study design (non-RCT, opinion, commentary, single-arm, etc.) |
| Dellis et al. Management of advanced prostate cancer: A systematic review of existing guidelines and recommendations. Cancer Treatment Reviews. 2019 | On topic SLR/MA/NMA |
| De Nunzio et al. Overall adverse events in patients treated with abiraterone and enzalutamide for metastatic castration resistant prostate cancer: Meta-analysis of randomized clinical trials and real-world reporting patterns from eudra vigilance. Journal of Urology. 2019 | On topic SLR/MA/NMA |
| De Nunzio et al. Overall adverse events in patients treated with abiraterone and enzalutamide for metastatic castration resistant prostate cancer: Meta-analysis of randomized clinical trials and real world reporting patterns from Eudra Vigilance. European Urology, Supplements. 2019 | Study design (non-RCT, opinion, commentary, single-arm, etc.) |
| Coutinho Mariano et al. Risk of falls and fractures in patients with castration resistant prostate cancer (CRPC) treated with new hormonal agents: A meta-analysis of randomized controlled trials. Annals of Oncology. 2019 | On topic SLR/MA/NMA |
| Chi et al. Updated results from a randomized phase II study of cabazitaxel (CAB) versus abiraterone (ABI) or enzalutamide (ENZ) in poor prognosis metastatic CRPC. Journal of clinical oncology. 2019 | Duplicate |
| Chi et al. Updated results from a randomized phase II study of cabazitaxel (CAB) versus abiraterone (ABI) or enzalutamide (ENZ) in poor prognosis metastatic CRPC. Journal of Clinical Oncology. 2019 | Mixed population |
| Caffo et al. A multicentric phase II randomized trial of docetaxel (D) plus enzalutamide (E) versus docetaxel (D) as first-line chemotherapy for patients (pts) with metastatic castration-resistant prostate cancer (mCRPC): CHEIRON study. Journal of clinical oncology. 2019 | Mixed population |
| Caffo et al. Updated survival analyses of a multicentric phase II randomized trial of docetaxel (D) plus enzalutamide (E) versus docetaxel (D) as first-line chemotherapy for patients (pts) with metastatic castration-resistant prostate cancer (mCRPC) (CHEIRON study). Annals of Oncology. 2019 | Mixed population |
| Caffo et al. A multicentric phase II randomized trial of docetaxel (D) plus enzalutamide (E) versus docetaxel (D) as first-line chemotherapy for patients (pts) with metastatic castration-resistant prostate cancer (mCRPC): CHEIRON study. Journal of Clinical Oncology. 2019 | Duplicate |
| Baciarello et al. Final results from the randomized CABADOC trial: patient preference between cabazitaxel and docetaxel for first-line chemotherapy in metastatic castrate-resistant prostate cancer (mCRPC). Journal of clinical oncology. 2019 | Duplicate |
| Baciarello et al. Final results from the randomized CABADOC trial: Patient preference between cabazitaxel and docetaxel for first-line chemotherapy in metastatic castrate-resistant prostate cancer (mCRPC). Journal of Clinical Oncology. 2019 | Study design (non-RCT, opinion, commentary, single-arm, etc.) |
| Armstrong et al. Enzalutamide in men with chemotherapy-naive metastatic castration-resistant prostate cancer (mCRPC): Long-term overall survival and safety analyses of the phase 3 PREVAIL study. European Urology, Supplements. 2019 | Population |
| Alonzi et al. Fracture risk after radium-223 (Ra-223) in metastatic castration resistant prostate cancer (mCRPC). Journal of clinical oncology. 2019 | Duplicate |
| Alonzi et al. Fracture risk after radium-223 (Ra-223) in metastatic castration resistant prostate cancer (mCRPC). Journal of Clinical Oncology. 2019 | Mixed population |
| Yachnin et al. Weekly versus 3-weekly cabazitaxel for the treatment of castration-resistant prostate cancer: A randomised phase II trial (ConCab). European Journal of Cancer. 2018 | Population |
| Wang et al. Effectiveness and tolerability of targeted drugs for the treatment of metastatic castration-resistant prostate cancer: a network meta-analysis of randomized controlled trials. Journal of Cancer Research and Clinical Oncology. 2018 | On topic SLR/MA/NMA |
| Szmulewitz et al. Prospective International Randomized Phase II Study of Low-Dose Abiraterone With Food Versus Standard Dose Abiraterone In Castration-Resistant Prostate Cancer. Journal of Clinical Oncology. 2018 | Incomplete/Insufficient/Partial data |
| Sternberg et al. A randomized phase 2 study investigating 3 dosing regimens of radium-223 dichloride (Ra-223) in bone metastatic castrationresistant prostate cancer (mCRPC). Journal of clinical oncology. 2018 | Old conference abstracts (before 2019) |
| Smith et al. ERA 223: a phase III trial of radium-223 (Ra-223) in combination with abiraterone acetate and prednisone/prednisolone for the treatment of asymptomatic or mildly symptomatic chemotherapy-naA[spacing macron]ve patients (PTS) with bone-predominant metastatic castration-resistant prostate cancer (mCRPC). Annals of oncology. 2018 | Old conference abstracts (before 2019) |
| Siemens et al. Efficacy and Safety of Enzalutamide vs Bicalutamide in Younger and Older Patients with Metastatic Castration Resistant Prostate Cancer in the TERRAIN Trial. Journal of urology. 2018 | Duplicate |
| Rathkopf et al. Radiographic progression-free survival as a clinically meaningful end point in metastatic castration-resistant prostate cancer: The PREVAIL randomized clinical trial. JAMA Oncology. 2018 | Study design (non-RCT, opinion, commentary, single-arm, etc.) |
| Petrylak et al. Overall survival and immune responses with sipuleucel-T and enzalutamide: STRIDE study. Journal of clinical oncology. 2018 | Old conference abstracts (before 2019) |
| Parker et al. Three-year Safety of Radium-223 Dichloride in Patients with Castration-resistant Prostate Cancer and Symptomatic Bone Metastases from Phase 3 Randomized Alpharadin in Symptomatic Prostate Cancer Trial. European urology. 2018 | Duplicate |
| Parker et al. Radium-223: disease response and fracture assessment by whole body diffusionweighted MRI (WB-DWMRI) in metastatic castration resistant prostate cancer (mCRPC). Journal of clinical oncology. 2018 | Old conference abstracts (before 2019) |
| Parker et al. Three-year Safety of Radium-223 Dichloride in Patients with Castration-resistant Prostate Cancer and Symptomatic Bone Metastases from Phase 3 Randomized Alpharadin in Symptomatic Prostate Cancer Trial. European Urology. 2018 | Mixed population |
| Ohlmann et al. Abiraterone acetate plus prednisone and LHRH therapy versus abiraterone acetate plus prednisone while sparing LHRH therapy in patients with progressive, metastatic and chemotherapy-naA[spacing macron]ve, castration-resistant prostate cancer: results from the SPARE-trial (NCT02077634). Annals of oncology. 2018 | Old conference abstracts (before 2019) |
| McCool et al. Systematic Review and Network Meta-Analysis of Treatments for Chemotherapy-Naive Patients with Asymptomatic/Mildly Symptomatic Metastatic Castration-Resistant Prostate Cancer. Value in Health. 2018 | On topic SLR/MA/NMA |
| Maughan et al. Safety data from a phase II randomized trial of radium-223 dichloride (Ra-223) plus enzalutamide (Enza) vs. Enza alone in men with metastatic castration refractory prostate cancer (mCRPC). Journal of clinical oncology. 2018 | Old conference abstracts (before 2019) |
| Maughan et al. Randomized phase II trial of radium-223 (RA) plus enzalutamide (EZ) vs. EZ alone in metastatic castration refractory prostate cancer (mCRPC). Annals of oncology. 2018 | Population |
| Matsubara et al. Phase II study of radium-223 dichloride in Japanese patients with symptomatic castration-resistant prostate cancer. International Journal of Clinical Oncology. 2018 | Study design (non-RCT, opinion, commentary, single-arm, etc.) |
| Khalaf et al. Health-related Quality of Life for Abiraterone Plus Prednisone Versus Enzalutamide in Patients with Metastatic Castration-resistant Prostate Cancer: results from a Phase II Randomized Trial. European urology. 2018 | Duplicate |
| Juarez et al. [Abiraterone in castration resistant prostate cancer.]. Abiraterona en cancer de prostata resistente a la castracion. 2018 | Non-English |
| Denunzio et al. Castration-resistance prostate cancer: What is in the pipeline?. Minerva Urologica e Nefrologica. 2018 | On topic SLR/MA/NMA |
| Corman et al. Sipuleucel-T (SIP-T) with enzalutamide (ENZ) (STRIDE): clinical outcomes in patients (pts) with metastatic castration-resistant prostate cancer (MCRPC) by baseline (BL) prostate-specific antigen (PSA) quartiles. Journal of urology. 2018 | Old conference abstracts (before 2019) |
| Chi et al. A randomized phase II study of cabazitaxel (CAB) vs (ABI) abiraterone or (ENZ) enzalutamide in poor prognosis metastatic castration-resistant prostate cancer (mCRPC). Annals of oncology : official journal of the european society for medical oncology. 2018 | Old conference abstracts (before 2019) |
| Radium-223 for metastatic castration-resistant prostate cancer: results and remaining open issues after the ALSYMPCA trial. 2018 | Study design (non-RCT, opinion, commentary, single-arm, etc.) |
| Hepatic effects assessed by review of safety data in enzalutamide castration-resistant prostate cancer (CRPC) trials. 2018 | Old conference abstracts (before 2019) |
| ERA 223: a phase III trial of radium-223 (Ra-223) in combination with abiraterone acetate and prednisone/prednisolone for the treatment of asymptomatic or mildly symptomatic chemotherapy-naA[spacing macron]ve patients (pts) with bone-predominant metastatic castration-resistant prostate cancer (mCRPC). 2018 | Old conference abstracts (before 2019) |
| Abiraterone acetate plus prednisone and LHRH therapy versus abiraterone acetate plus prednisone while sparing LHRH therapy in patients with progressive, metastatic and chemotherapy-naA[spacing macron]ve, castration-resistant prostate cancer: results from the SPARE-trial (NCT02077634). 2018 | Old conference abstracts (before 2019) |
| Zheng et al. Safety and Efficacy of First-Line Treatments for Chemotherapy-Naive Metastatic Castration-Resistant Prostate Cancer: A Systematic Review and Indirect Comparison. BioMed Research International. 2017 | On topic SLR/MA/NMA |
| Ye et al. A phase 3, double-blind, randomized placebo-controlled efficacy and safety study of abiraterone acetate in chemotherapy-naive patients with mCRPC in China, Malaysia, Thailand and Russia. Asian journal of urology. (no pagination). 2017 | Duplicate |
| Twardowski et al. Randomized phase II trial of sipuleucel-T Immunotherapy preceded by sensitizing radiation therapy and sipuleucel-t alone in patients with metastatic castrate resistant prostate cancer. Journal of clinical oncology. 2017 | Old conference abstracts (before 2019) |
| Szmulewitz et al. A prospective international randomized phase II study evaluating the food effect on the pharmacokinetics (PK) and pharmacodynamics (PD) of abiraterone acetate (AA) in men with castration-resistant prostate cancer (CRPC). Journal of clinical oncology. 2017 | Outcome |
| Stein et al. Randomized phase 2 therapeutic equivalence study of abiraterone acetate fine particle formulation vs. originator abiraterone acetate in patients with metastatic castration-resistant prostate cancer: the STAAR study. Urologic oncology: seminars and original investigations. 2017 | Duplicate |
| Small et al. Long-term follow-up from STAMP, a phase II trial, evaluating sipuleucel-T and concurrent (CON) vs sequential (SEQ) abiraterone acetate + prednisone in metastatic castration-resistant prostate cancer patients (pts). Journal of clinical oncology. 2017 | Old conference abstracts (before 2019) |
| Saad et al. Skeletal-related events significantly impact health-related quality of life in metastatic castration-resistant prostate cancer: data from PREVAIL and AFFIRM trials. Prostate cancer and prostatic diseases. 2017 | Duplicate |
| Poorthuis et al. First-line non-cytotoxic therapy in chemotherapy-naive patients with metastatic castration-resistant prostate cancer: a systematic review of 10 randomised clinical trials. BJU International. 2017 | On topic SLR/MA/NMA |
| Maria et al. Association of risk of febrile neutropenia (FN) with docetaxel in prostate cancer (PC) patients: a meta-analysis of published phase II-III trials. Journal of clinical oncology. 2017 | Old conference abstracts (before 2019) |
| Khalaf et al. Assessment of quality of life (QOL), cognitive function and depression in a randomized phase II study of abiraterone acetate (ABI) plus prednisone (P) vs enzalutamide (ENZA) for metastatic castrate-resistant prostate cancer (mCRPC). Journal of clinical oncology. 2017 | Old conference abstracts (before 2019) |
| Kang et al. Comparing the clinical efficacy of abiraterone acetate, enzalutamide, and orteronel in patients with metastatic castration-resistant prostate cancer by performing a network meta-analysis of eight randomized controlled trials. Oncotarget. 2017 | On topic SLR/MA/NMA |
| Francini et al. Differential side effects profile in patients with mCRPC treated with abiraterone or enzalutamide: A meta-analysis of randomized controlled trials. Oncotarget. 2017 | On topic SLR/MA/NMA |
| Fizazi et al. Patient preference between Cabazitaxel and Docetaxel for first-line chemotherapy in metastatic castrate-resistant prostate cancer (mCRPC): results from the CABADOC randomized trial. Annals of oncology. 2017 | Old conference abstracts (before 2019) |
| Chi et al. A randomized phase II cross-over study of abiraterone + prednisone (ABI) vs enzalutamide (ENZ) for patients (pts) with metastatic, castration-resistant prostate cancer (mCRPC). Journal of clinical oncology. 2017 | Old conference abstracts (before 2019) |
| Antonarakis et al. Randomized, noncomparative, phase II trial of early switch from docetaxel to cabazitaxel or vice versa, with integrated biomarker analysis, in men with chemotherapy-naive, metastatic, castration-resistant prostate cancer. Journal of Clinical Oncology. 2017 | Mixed population |
| Post hoc analyses of East Asian patients from the randomized placebo-controlled PREVAIL trial of enzalutamide in patients with chemotherapy-naive, metastatic castration-resistant prostate cancer. 2017 | Duplicate |
| The Phase 3 COU-AA-302 Study of Abiraterone Acetate Plus Prednisone in Men with Chemotherapy-naive Metastatic Castration-resistant Prostate Cancer: stratified Analysis Based on Pain, Prostate-specific Antigen, and Gleason Score. 2017 | Duplicate |
| The TRITON clinical trial programme: evaluation of the PARP inhibitor rucaparib in patients with metastatic castration-resistant prostate cancer (mCRPC) associated with homologous recombination deficiency (HRD). 2017 | Population |
| Zhang et al. Docetaxel in the treatment of metastatic hormone-sensitive and castration-resistant prostate cancer: a meta-analysis. Journal of clinical oncology. 2016 | Old conference abstracts (before 2019) |
| Ye et al. Efficacy, safety and pharmacokinetics (PK) of enzalutamide (ENZ) vs placebo (PL) in chemotherapy-naive patients (pts) with progressive metastatic castration-resistant prostate cancer (mCRPC): an Asian multinational study. Annals of oncology. 2016 | Old conference abstracts (before 2019) |
| Ye et al. Efficacy and safety of enzalutamide (ENZ) vs placebo (PL) in chemotherapy-naive patients (pts) with progressive metastatic castration-resistant prostate cancer (mCRPC) following androgen deprivation therapy (ADT): an Asian multinational study. Annals of oncology. 2016 | Duplicate |
| Ye, et al. Efficacy and safety of enzalutamide (ENZ) vs placebo (PL) in chemotherapy-naive patients (pts) with progressive metastatic castration-resistant prostate cancer (mCRPC) following androgen deprivation therapy (ADT): An Asian multinational study. Annals of Oncology. 2016 | Old conference abstracts (before 2019) |
| Wirth et al. Analysis of overall survival by number of radium-223 injections received in an international expanded access program (iEAP). Oncology research and treatment. 2016 | Study design (non-RCT, opinion, commentary, single-arm, etc.) |
| Tagawa et al. TAXYNERGY: randomized trial of early switch from first- line docetaxel (D) to cabazitaxel (C) or vice versa with circulating tumor cell (CTC) biomarkers in patients (pts) with metastatic castration-resistant prostate cancer (mCRPC). Journal of clinical oncology. 2016 | Old conference abstracts (before 2019) |
| Sigala et al. Role of the novel generation of androgen receptor pathway targeted agents in the management of castration-resistant prostate cancer: A literature based meta-analysis of randomized trials. European Journal of Cancer. 2016 | On topic SLR/MA/NMA |
| Sigala et al. Incidence and relative risk of adverse events of special interest in patients with castration resistant prostate cancer treated with CYP-17 inhibitors: A meta-analysis of published trials. Critical Reviews in Oncology/Hematology. 2016 | On topic SLR/MA/NMA |
| Sartor et al. Chemotherapy following radium-223 dichloride treatment in ALSYMPCA. Prostate. 2016 | Population |
| Sartor et al. Cabazitaxel vs docetaxel in chemotherapy-naive (CN) patients with metastatic castration-resistant prostate cancer (mCRPC): a three-arm phase III study (FIRSTANA). Journal of clinical oncology. 2016 | Old conference abstracts (before 2019) |
| Ruiz Gracia et al. Meta-analysis of randomized clinical trials in metastatic castration resistant prostate cancer: comparison of hypertension, neurological and psychiatric adverse events on enzalutamide and abiraterone acetate plus prednisone treatment. Annals of oncology. 2016 | On topic SLR/MA/NMA |
| Roviello, et al. Targeting the androgenic pathway in elderly patients with castration-resistant prostate cancer: A meta-analysis of randomized trials. Medicine. 2016 | On topic SLR/MA/NMA |
| Rexer et al. Study on the therapy of castration-resistant prostate cancer: Randomized phase II study of abiraterone acetate with LHRH therapy vs. abiraterone acetate without LHRH therapy in patients with progressive chemotherapy-naive castration-resistant prostate cancer (SPARE) - AP 67/11 of the Association of Urogenital Oncology (AUO). Urologe. 2016 | Non-English |
| Parker et al. Efficacy and Safety of Radium-223 Dichloride in Symptomatic Castration-resistant Prostate Cancer Patients With or Without Baseline Opioid Use From the Phase 3 ALSYMPCA Trial. European Urology. 2016 | Mixed population |
| Parimi et al. Effects of abiraterone (ABI) and enzalutamide (ENZA) on cognitive impairment and depressive symptoms in patients (pts) with metastatic castration-resistant prostate cancer (mCRPC). Journal of clinical oncology. 2016 | Old conference abstracts (before 2019) |
| Oudard et al. FIRSTANA: health-related quality of life (HRQL) and post-hoc analyses for the phase III study assessing cabazitaxel (C) vs docetaxel (D) in chemotherapy-naive patients (pts) with metastatic castration-resistant prostate cancer (mCRPC). Annals of oncology. 2016 | Duplicate |
| Oudard et al. FIRSTANA: Health-related quality of life (HRQL) and post-hoc analyses for the phase III study assessing cabazitaxel (C) vs docetaxel (D) in chemotherapy-naive patients (pts) with metastatic castration-resistant prostate cancer (mCRPC). Annals of Oncology. 2016 | Old conference abstracts (before 2019) |
| Nilsson et al. Patient-reported quality-of-life analysis of radium-223 dichloride from the phase III ALSYMPCA study. Annals of Oncology. 2016 | Mixed population |
| Liepe et al, From palliative therapy to prolongation of survival: 223RaCl2 in the treatment of bone metastases. Therapeutic Advances in Medical Oncology. 2016 | Study design (non-RCT, opinion, commentary, single-arm, etc.) |
| Lebdai et al, What do we know about treatment sequencing of abiraterone, enzalutamide, and chemotherapy in metastatic castration-resistant prostate cancer?. World journal of urology. 2016 | Population |
| Kim et al. The PREVAIL trial of enzalutamide in men with chemotherapy-naA[spacing macron]ve, metastatic castration-resistant prostate cancer: post hoc analysis of Korean patients. Investig clin urol. 2016 | Duplicate |
| Grande et al. Early responses to enzalutamide in AR-V7 positive first line metastatic castration-resistant prostate cancer (mCRPC). A prospective SOGUG clinical trial: The PREMIERE study. Annals of Oncology. 2016 | Study design (non-RCT, opinion, commentary, single-arm, etc.) |
| Gracia et al. Meta-analysis of randomized clinical trials in metastatic castration resistant prostate cancer: Comparison of hypertension, neurological and psychiatric adverse events on enzalutamide and abiraterone acetate plus prednisone treatment. Annals of Oncology. 2016 | Duplicate |
| Corfield et al. Understanding the role of new systemic agents in the treatment of prostate cancer. BJU International. 2016 | On topic SLR/MA/NMA |
| Colloca et al. Incidence and Correlates of Fatigue in Metastatic Castration-Resistant Prostate Cancer: A Systematic Review. Clinical Genitourinary Cancer. 2016 | On topic SLR/MA/NMA |
| Chowdhury et al. Fatigue in men with metastatic castration-resistant prostate cancer treated with enzalutamide: data from randomised clinical trials. Annals of Oncology. 2016 | Duplicate |
| Carles et al. Safety of long-term (LT) treatment (tmt) of chemotherapy (chemo)-naive metastatic castration-resistant prostate cancer (mCRPC) patients (pts) with abiraterone acetate plus prednisone (AA + P) for >= 4 years (yrs). Annals of oncology. 2016 | Old conference abstracts (before 2019) |
| Beer et al. Efficacy and safety of enzalutamide in patients 75 years or older with chemotherapy-naive metastatic castrationresistant prostate cancer: Results from PREVAIL. Annals of Oncology. 2016 | Duplicate |
| Fatigue in men with metastatic castration-resistant prostate cancer treated with enzalutamide: data from randomised clinical trials. 2016 | Old conference abstracts (before 2019) |
| Efficacy and safety of enzalutamide (ENZ) vs placebo (PL) in chemotherapy-naA[spacing macron]ve patients (pts) with progressive metastatic castration-resistant prostate cancer (mCRPC) following androgen deprivation therapy (ADT): an Asian multinational study. 2016 | Duplicate |
| Verri et al. The incidence and relative risk of cardiovascular toxicity in patients treated with new hormonal agents for castration-resistant prostate cancer. European Journal of Cancer. 2015 | On topic SLR/MA/NMA |
| Tunio et al. Comparative efficacy, tolerability, and survival outcomes of various radiopharmaceuticals in castration-resistant prostate cancer with bone metastasis: A meta-analysis of randomized controlled trials. Drug Design, Development and Therapy. 2015 | On topic SLR/MA/NMA |
| Takahashi et al. Administration of Docetaxel over 70 Cycles for Castration-Resistant Prostate Cancer. Clinical Genitourinary Cancer. 2015 | Study design (non-RCT, opinion, commentary, single-arm, etc.) |
| Sun et al. Efficacy and safety of abiraterone acetate in asymptomatic or mildly symptomatic chemotherapy-naive patients with metastatic castrationresistant prostate cancer: a phase III, double-blind, randomized, placebo-controlled study conducted in China, Malaysia, Thailand and Russia. BJU international. 2015 | Old conference abstracts (before 2019) |
| Strolin et al. External-beam radiation therapy (EBRT) use and safety with Radium-223 dichloride (Ra) in patients (pts) with castrationresistant prostate cancer (CRPC) and symptomatic bone metastases (mets) from the ALSYMPCA trial. 2015 | Duplicate |
| Strolin et al. Effects of radium-223 dichloride (Ra-223) on health-related quality of life (HRQoL) assessed by the EQ-5D utility scores in ALSYMPCA. 2015 | Old conference abstracts (before 2019) |
| Strauss et al. Impact of prior docetaxel, Extent of Disease (EOD), and prior Bisphosphonates (Bp) on Hematologic (Heme) safety of radium-223 Dichloride (Ra-223) from ALSYMPCA. 2015 | Old conference abstracts (before 2019) |
| Shore et al. Terrain trial: Prostate-specific antigen kinetics and quality of life results of enzalutamide versus bicalutamide in metastatic castration-resistant prostate cancer. Journal of urology. 2015 | Old conference abstracts (before 2019) |
| Shameem et al. Comparative analysis of the effectiveness of abiraterone before and after docetaxel in patients with metastatic castration-resistant prostate cancer. World journal of clinical oncology. 2015 | On topic SLR/MA/NMA |
| Sartor et al. 3-year follow-up of chemotherapy following radium-223 dichloride (Ra-223) in castration-resistant prostate cancer (CRPC) patients (Pts) with symptomatic bone metastases (Mets) from ALSYMPCA. European Journal of Cancer. ( var.pagings). 2015 | Mixed population |
| Qi et al. Efficacy and toxicity of molecular targeted therapies in combination with docetaxel for metastatic castration-resistant prostate cancer: A meta-analysis of phase III randomized controlled trials. Journal of Chemotherapy. 2015 | Study design (non-RCT, opinion, commentary, single-arm, etc.) |
| Petrylak et al. Immune responses and clinical data from STRIDE, a randomized, phase 2, open label study of sipuleucel-T with concurrent vs sequential enzalutamide administration in metastatic castrationresistant prostate cancer. European Journal of Cancer. ( var.pagings). 2015 | Old conference abstracts (before 2019) |
| Parker et al. 3-year safety follow-up of radium-223 dichloride (Ra-223) in patients (Pts) with castration-resistant prostate cancer (CRPC) and symptomatic bone metastases (Mets) from ALSYMPCA. Journal of clinical oncology. 2015 | Old conference abstracts (before 2019) |
| Pal et al. Cabazitaxel for the therapy of metastatic castration-resistant prostate cancer in the aftermath of the CHAARTED trial. BJU International. 2015 | Study design (non-RCT, opinion, commentary, single-arm, etc.) |
| Mulders et al. Abiraterone acetate improves overall survival in chemotherapy-naive metastatic castrationresistant prostate cancer (mCRPC): Impact of crossover and baseline prognostic factors in the COU-AA-302 final analysis. European Urology, Supplements. 2015 | Old conference abstracts (before 2019) |
| Kimura et al. Subgroup analyses of Japanese patients from the PREVAIL trial of enzalutamide (ENZA) in patients with chemotherapy-naive, metastatic castration-resistant prostate cancer (mCRPC). Journal of clinical oncology. 2015 | Old conference abstracts (before 2019) |
| Kim et al. The PREVAIL trial of enzalutamide (ENZA) in men with chemotherapynaive, metastatic castrationresistant prostate cancer (mCRPC): subgroup analysis of Asian patients. BJU international. 2015 | Old conference abstracts (before 2019) |
| Graff et al. Clinical outcomes and safety in men >75 and < 75 years with metastatic castration-resistant prostate cancer (mCRPC) treated with enzalutamide in the phase 3 PREVAIL trial. Journal of clinical oncology. 2015 | Old conference abstracts (before 2019) |
| Graff et al. Reducing skeletal-related events in metastatic castration-resistant prostate cancer. ONCOLOGY (United States). 2015 | Study design (non-RCT, opinion, commentary, single-arm, etc.) |
| Finkelstein et al. External beam radiation therapy (EBRT) use and safety with radium-223 dichloride (Ra-223) in patients (pts) with castration-resistant prostate cancer (CRPC) and symptomatic bone metastases (mets) from the ALSYMPCA trial. Journal of clinical oncology. 2015 | Old conference abstracts (before 2019) |
| Finkelstein et al. External beam radiation therapy (EBRT) use and safety with radium-223 dichloride (RA-223) in patients with castration-resistant prostate cancer (CRPC) and symptomatic bone metastases (METS) from the alsympca trial. International Journal of Radiation Oncology Biology Physics. ( var.pagings). 2015 | Old conference abstracts (before 2019) |
| Devlin et al. Health-related quality of life (HRQOL) benefits of enzalutamide in patients with metastatic castration-resistant prostate cancer (MCRPC): An in-depth analysis of EQ-5D data from the prevail trial. Value in health. 2015 | Old conference abstracts (before 2019) |
| De Souza et al. Final analysis of the randomised, phase 3, COU-AA-302 study of abiraterone acetate (AA) in chemotherapy-naive patients with metastatic castration-resistant prostate cancer (mCRPC). BJU international. 2015 | Old conference abstracts (before 2019) |
| Cox et al. Radium-223 for the Management of Bone Metastases in Castration-Resistant Prostate Cancer. Journal of the advanced practitioner in oncology. 2015 | Study design (non-RCT, opinion, commentary, single-arm, etc.) |
| Amadori et al. 3-year safety follow-up of radium-223 dichloride (Ra-223) in patients (Pts) with castration resistant prostate cancer (CRPC) and symptomatic bone metastases (Mets) from ALSYMPCA. Annals of Oncology. 2015 | Old conference abstracts (before 2019) |
| Efficacy and Safety of Abiraterone Acetate in Elderly (75 Years or Older) Chemotherapy Naive Patients with Metastatic Castration Resistant Prostate Cancer. Journal of urology. 2015 | Duplicate |
| Zhou et al. Abiraterone for treatment of metastatic castration-resistant prostate cancer: A systematic review and meta-analysis. Asian Pacific Journal of Cancer Prevention. 2014 | On topic SLR/MA/NMA |
| West et al. Estimating scenarios for survival time in men starting systemic therapies for castration-resistant prostate cancer: A systematic review of randomised trials. European Journal of Cancer. 2014 | On topic SLR/MA/NMA |
| Tombal et al. Enzalutamide in men with chemotherapy-naive metastatic castration resistant prostate cancer (MCRPC): Primary and European regional results of the phase 3 prevail study. European Urology, Supplements. 2014 | Old conference abstracts (before 2019) |
| Small et al. Time to disease-related pain and first opioid use in patients with metastatic castration-resistant prostate cancer treated with sipuleucel-T. Prostate Cancer and Prostatic Diseases. 2014 | Study design (non-RCT, opinion, commentary, single-arm, etc.) |
| Shore et al. Further characterization of the effects of prior or no prior docetaxel therapy on CRPC patients with bone metastases receiving Ra-223 in the phase 3 alsympca trial. Journal of urology. 2014 | Old conference abstracts (before 2019) |
| Shore et al. Efficacy and long-term safety analysis of study COU-AA-302: Abiraterone acetate plus prednisone in chemotherapy-naive metastatic castration-resistant prostate cancer. Journal of urology. 2014 | Old conference abstracts (before 2019) |
| Sartor et al. Effect of radium-223 dichloride on symptomatic skeletal events in patients with castration-resistant prostate cancer and bone metastases: Results from a phase 3, double-blind, randomised trial. The Lancet Oncology. 2014 | Mixed population |
| Saad et al. The prevail study: Primary and non-visceral/visceral disease subgroup results for enzalutamide-treated men with metastatic castration-resistant prostate cancer. Urology. 2014 | Old conference abstracts (before 2019) |
| Quinn et al. A randomized phase II, open-label study of sipuleucel-T with concurrent or sequential enzalutamide in metastatic castration-resistant prostate cancer (mCRPC). Journal of clinical oncology. 2014 | Old conference abstracts (before 2019) |
| Petrylak et al. A randomized open-label phase 2a study evaluating the efficacy and safety of radium-223 dichloride (Ra-223) in combination with abiraterone acetate or enzalutamide in patients with castration-resistant prostate cancer (CRPC) and bone metastases. Journal of clinical oncology. 2014 | Study design (non-RCT, opinion, commentary, single-arm, etc.) |
| O'Sullivan et al. Effects of Radium-223 Dichloride on health-related QOL in CRPC Pts with bone mets from the Ph3 ALSYMPCA trial. Radiotherapy and oncology. 2014 | Mixed population |
| Nilsson et al. 1.5-year post-treatment follow-up of radium-223 dichloride (Ra-223) in patients with castration-resistant prostate cancer (CRPC) and bone metastases from the phase 3 ALSYMPCA study. Journal of clinical oncology. 2014 | Mixed population |
| Matsubara et al. A phase 2 trial of abiraterone acetate in Japanese men with metastatic castration-resistant prostate cancer and without prior chemotherapy (JPN-201 Study). Japanese Journal of Clinical Oncology. 2014 | Study design (non-RCT, opinion, commentary, single-arm, etc.) |
| Kellokumpu-Lehtinen et al. Triweekly docetaxel versus biweekly docetaxel as a treatment for advanced castration resistant prostate cancer: Quality of life analysis. Journal of clinical oncology. 2014 | Old conference abstracts (before 2019) |
| Hoskin et al. Efficacy and safety of radium-223 dichloride in patients with castration-resistant prostate cancer and symptomatic bone metastases, with or without previous docetaxel use: A prespecified subgroup analysis from the randomised, double-blind, phase 3 ALSYMPCA trial. The Lancet Oncology. 2014 | Mixed population |
| Beer et al. Enzalutamide in men with chemotherapy-naive metastatic prostate cancer (mCRPC): Results of phase III PREVAIL study. Journal of clinical oncology. 2014 | Old conference abstracts (before 2019) |
| Armstrong et al. Primary, secondary, and quality-of-life endpoint results from PREVAIL, a phase 3 study of enzalutamide in men with metastatic castration resistant prostate cancer (mCRPC). Journal of clinical oncology. 2014 | Old conference abstracts (before 2019) |
| Aragon-Ching et al. Further analysis of PREVAIL: Enzalutamide use in chemotherapy-naive men with metastatic castration-resistant prostate cancer. Asian Journal of Andrology. 2014 | Study design (non-RCT, opinion, commentary, single-arm, etc.) |
| Anonymous. ODM-201 is safe and active in metastatic castration-resistant prostate cancer. Cancer Discovery. 2014 | Study design (non-RCT, opinion, commentary, single-arm, etc.) |
| Wiechno et al. Radium-223 dichloride (Ra-223) efficacy and safety in patients with castration-resistant prostate cancer (CRPC) with bone metastases: Phase 3 ALSYMPCA study findings stratified by age group. European journal of cancer. 2013 | Mixed population |
| Widmark et al. Hematologic safety profile of radium-223 dichloride (Ra-223) from the phase 3 ALSYMPCA trial in castration-resistant prostate cancer (CRPC) patients with bone metastases. European journal of nuclear medicine and molecular imaging. 2013 | Mixed population |
| Wedel et al. Updated analysis of radium-223 dichloride (Ra-223) impact on survival, safety, and skeletal-related events in castration-resistant prostate cancer (CRPC) patients with bone metastases from the phase 3 ALSYMPCA trial. European journal of nuclear medicine and molecular imaging. 2013 | Mixed population |
| Vogelzang et al. Updated analysis of radium-223 dichloride (Ra-223) impact on skeletal-related events (SRE) in patients with castration-resistant prostate cancer (CRPC) and bone metastases from the phase III randomized trial (ALSYMPCA). Journal of clinical oncology. 2013 | Mixed population |
| Vogelzang et al. Efficacy and safety of radium-223 dichloride (Ra-223) in castration-resistant prostate cancer (CRPC) patients with bone metastases who did or did not receive prior docetaxel (D) in the phase III ALSYMPCA trial. Journal of clinical oncology. 2013 | Old conference abstracts (before 2019) |
| Van Poppel et al. Updated interim analysis (IA): Results of randomized phase 3 study COUAA-302 of abiraterone acetate (AA) in metastatic castration-resistant prostate cancer (mCRPC) patients (pts) without prior chemotherapy. European Urology, Supplements. 2013 | Old conference abstracts (before 2019) |
| Straus et al. Hematologic safety of Ra-223 dichloride (Ra-223) in castration-resistant prostate cancer (CRPC) patients with bone metastases from the phase 3 ALSYMPCA trial. Onkologie. 2013 | Old conference abstracts (before 2019) |
| Steuber et al. Updated Survival, Quality of life (QOL) and Safety-data of Radium223 Dichlorid (Ra223) in patients with castration resistant prostate cancer (CRPC) with bone metastasis from the Phase 3 double-blind randomised, multinational study (ALSYMPCA). Onkologie. 2013 | Old conference abstracts (before 2019) |
| Small et al. A randomized phase II trial of sipuleucel-T with concurrent or sequential abiraterone acetate (AA) plus prednisone (P) in metastatic castrate-resistant prostate cancer (mCRPC). Journal of clinical oncology. 2013 | Old conference abstracts (before 2019) |
| Small et al. A randomized phase II, open-label study of sipuleucel-T with concurrent or sequential abiraterone acetate (AA) in metastatic castrate-resistant prostate cancer (mCRPC). Journal of clinical oncology. 2013 | Old conference abstracts (before 2019) |
| Small et al. A Phase 2 trial of sipuleucel-T in combination with concurrent or sequential abiraterone acetate (AA) in patients (pts) with metastatic castrate-resistant prostate cancer (mCRPC). European journal of cancer. 2013 | Old conference abstracts (before 2019) |
| Shore et al. The impact of abiraterone acetate therapy on patient-reported pain and functional status in chemotherapy-naive patients with progressive, metastatic castration-resistant prostate cancer-results from an updated analysis. Journal of urology. 2013 | Old conference abstracts (before 2019) |
| Seal et al. Efficacy, patient-reported outcomes (PROs), and tolerability of the changing therapeutic landscape in patients with metastatic prostate cancer (MPC): a systematic literature review. Value in health : the journal of the International Society for Pharmacoeconomics and Outcomes Research. 2013 | On topic SLR/MA/NMA |
| Sartor et al. Radium-223(R a-223) safety and efficacy in prostate cancer with bone metastases: Phase 3 ALSYMPCA study findings stratified by age. BJU international. 2013 | Old conference abstracts (before 2019) |
| Rathkopf et al. Long-term safety and efficacy analysis of abiraterone acetate (AA) plus prednisone (P) in metastatic castration-resistant prostate cancer (mCRPC) without prior chemotherapy (COU-AA-302). Journal of clinical oncology. 2013 | Old conference abstracts (before 2019) |
| Raedler et al. (radium Ra 223 dichloride): The first alpha particle-emitting radioactive agent for the treatment of castration-resistant prostate cancer with symptomatic bone metastases. American Health and Drug Benefits. 2013 | Study design (non-RCT, opinion, commentary, single-arm, etc.) |
| Parker et al. Hematologic safety of Ra-223 dichloride (Ra-223) in castration-resistant prostate cancer (CRPC) patients with bone metastases from the phase III ALSYMPCA trial. Journal of clinical oncology. 2013 | Old conference abstracts (before 2019) |
| O'Sullivan et al. Hematologic safety of radium-223 dichloride (Ra-223) in the phase 3 ALSYMPCA trial in castration-resistant prostate cancer (CRPC) patients with bone metastases: Baseline prognostic factor subgroup analysis. European journal of cancer. 2013 | Mixed population |
| Nilsson et al. Long-termsafety of radium-223 dichloride (Ra-223) in patients with castration-resistant prostate cancer (CRPC) and bonemetastases from the phase 3 ALSYMPCA study. European Urology, Supplements. 2013 | Mixed population |
| Nilsson et al. Two-year survival follow-up of the randomized, double-blind, placebo-controlled phase II study of radium-223 chloride in patients with castration-resistant prostate cancer and bone metastases. Clinical Genitourinary Cancer. 2013 | Population |
| Miller et al. Abiraterone reduced morbidity in mCRPC. Oncology Report. 2013 | Study design (non-RCT, opinion, commentary, single-arm, etc.) |
| Michalski et al. Radium-223 dichloride (Ra-223) impact on skeletal-related events, external beam radiation therapy (EBRT), and pain in patients with castration-resistant prostate cancer (CRPC) with bone metastases: Updated results from the phase 3 alsympca trial. International Journal of Radiation Oncology Biology Physics. 2013 | Old conference abstracts (before 2019) |
| Loblaw et al. Genitourinary Cancer Disease Site Group of Cancer Care Ontario's Program in Evidence-Based, Care. Systemic therapy in men with metastatic castration-resistant prostate cancer: a systematic review. Clinical oncology (Royal College of Radiologists (Great Britain)). 2013 | On topic SLR/MA/NMA |
| Loblaw et al. Systemic Therapy in Men with Metastatic Castration-resistant Prostate Cancer: ASystematic Review. Clinical Oncology. 2013 | Duplicate |
| Koenig et al. Efficacy and safety of radium-223 dichloride (Ra-223) in castration-resistant prostate cancer (CRPC) patients with bone metastases who had prior or no-prior docetaxel (D) therapy in the phase 3 ALSYMPCA trial. Onkologie. 2013 | Mixed population |
| Heinrich et al. Updated analysis of radium-223 dichloride (Ra-223) impact on pain, skeletal-related events (SRE), and survival from the phase 3 randomized trial (ALSYMPCA) in patients with castration-resistant prostate cancer (CRPC) and bone metastases. European Urology, Supplements. 2013 | Mixed population |
| De Souza et al. Long-term safety and efficacy analysis of abiraterone acetate (AA) plus prednisone (P) in study COU-AA-302for metastatic castration-resistant prostate cancer. BJU international. 2013 | Old conference abstracts (before 2019) |
| Alpha emitter radium-223 and survival in metastatic prostate cancer. New England Journal of Medicine. 2013 | Mixed population |
| Yijun et al. Docetaxel plus prednisone versus mitoxantrone plus prednisone as firstline chemotherapy for metastatic castration-refractory prostate cancer: Long-term effects and safety in chinese. International journal of urology. 2012 | Old conference abstracts (before 2019) |
| Sonpavde et al. The role of sipuleucel-T in therapy for castration-resistant prostate cancer: A critical analysis of the literature. European Urology. 2012 | On topic SLR/MA/NMA |
| Small Ej, Higano C. S. Kantoff P. W. Whitmore J. B. Frohlich M. W. Petrylak D. P.. Time to disease-related pain (TDRP) following sipuleucel-T in asymptomatic patients with metastatic castrate-resistant prostate cancer (mCRPC): Integrated results from three randomized phase 3 trials. European Urology, Supplements. 2012 | Old conference abstracts (before 2019) |
| Shen et al. [Docetaxel plus prednisone versus mitoxantrone plus prednisone as first-line chemotherapy for metastatic hormone-refractory prostate cancer: long-term effects and safety]. Zhonghua wai ke za zhi [Chinese journal of surgery]. 2012 | Non-English |
| Sartor et al. Radium-223 chloride (alpharadin) impact on overall survival and skeletal-related events in patients with castration-resistant prostate cancer with bone metastases: A phase III randomized trial (ALSYMPCA). Journal of urology. 2012 | Old conference abstracts (before 2019) |
| Sartor et al. Radium-223 chloride impact on skeletal-related events in patients with castration-resistant prostate cancer (CRPC) with bone metastases: A phase III randomized trial (ALSYMPCA). Journal of clinical oncology. 2012 | Old conference abstracts (before 2019) |
| Ryan et al. Interim analysis (IA) results of COU-AA-302, a randomized, phase III study of abiraterone acetate (AA) in chemotherapy-naive patients (pts) with metastatic castration-resistant prostate cancer (mCRPC). Journal of clinical oncology. 2012 | Old conference abstracts (before 2019) |
| Pompeo et al. Immunotherapy with Sipuleucel-T (APC8015) in patients with metastatic castration-refractory prostate cancer (mCRPC): A systematic review and meta-analysis. International Braz J Urol. 2012 | On topic SLR/MA/NMA |
| Parker et al. Updated analysis of the phase III, double-blind, randomized, multinational study of radium-223 chloride in castration-resistant prostate cancer (CRPC) patients with bone metastases (ALSYMPCA). Journal of clinical oncology. 2012 | Old conference abstracts (before 2019) |
| Parker et al. Overall survival benefit and safety profile of radium-223 chloride, a first-in-class alpha-pharmaceutical: Results from a phase III randomized trial (ALSYMPCA) in patients with castration-resistant prostate cancer (CRPC) with bone metastases. Journal of clinical oncology. 2012 | Old conference abstracts (before 2019) |
| Parker et al. Overall survival benefit and impact on skeletal-related events for radium- 223 chloride (Alpharadin) in the treatment of castration-resistant prostate cancer (CRPC) patients with bone metastases: A phase III randomized trial (ALSYMPCA). European Urology, Supplements. 2012 | Old conference abstracts (before 2019) |
| Sartor et al. Radium-223 chloride (Ra-223) impact on skeletal-related events (SREs) and ECOG performance status (PS) in patients with castration-resistant prostate cancer (CRPC) with bone metastases: Interim results of a phase III trial (ALSYMPCA). Journal of clinical oncology. 2012 | Old conference abstracts (before 2019) |
| O'Sullivan et al. Results from a phase III randomized trial (ALSYMPCA) of radium-223 chloride, a first-in-class alpha-emitter, in patients with castration-resistant prostate cancer (CRPC) and bone metastases: Overall survival benefit and safety profile. European journal of nuclear medicine and molecular imaging. 2012 | Mixed population |
| Miller et al. Radium-223 chloride impact on skeletal-related events in patients with castration-resistant prostate cancer (CRPC) with bone metastases: A phase III randomized trial (ALSYMPCA). Onkologie. 2012 | Old conference abstracts (before 2019) |
| Kuczyk et al. Overall survival benefit and safety profile of radium-223 chloride, a first-in-class alpha-pharmaceutical: Results from a phase III randomized trial (ALSYMPCA) in patients with castration-resistant prostate cancer (CRPC) with bone metastases. Onkologie. 2012 | Old conference abstracts (before 2019) |
| Kawalec et al. Sipuleucel-T immunotherapy for castration-resistant prostate cancer. A systematic review and meta-analysis. Archives of Medical Science. 2012 | On topic SLR/MA/NMA |
| Di Lorenzoet al. Sipuleucel-T (Provenge) for castration-resistant prostate cancer. BJU International. 2012 | Study design (non-RCT, opinion, commentary, single-arm, etc.) |
| De Souza et al. Abiraterone acetate (AA) in chemotherapy-naive patients with metastatic castration-resistant prostate cancer (MCRPC): Results of interim analysis (IA) of COU-AA-302, a randomized phase 3 study. Asia-Pacific journal of clinical oncology. 2012 | Old conference abstracts (before 2019) |
| Chang et al. Radium-223: Down to the bone, and less is more. Oncology. 2012 | Study design (non-RCT, opinion, commentary, single-arm, etc.) |
| Anonymous. Novel targets, agents, and trials. Clinical Advances in Hematology and Oncology. 2012 | Study design (non-RCT, opinion, commentary, single-arm, etc.) |
| Anonymous. Approved agents and related trials. Clinical Advances in Hematology and Oncology. 2012 | Study design (non-RCT, opinion, commentary, single-arm, etc.) |
| Small et al. Time to disease-related pain after sipuleucel-T in asymptomatic patients with metastatic castrate-resistant prostate cancer (mCRPC): Results from three randomized phase III trials. Journal of clinical oncology. 2011 | Old conference abstracts (before 2019) |
| Shore et al. Central venous catheter utilization during the impact (D9902B) trial: A review of central venous catheter incidence and associated adverse events. Urology. 2011 | Old conference abstracts (before 2019) |
| Serpa et al. Ten years of docetaxel-based therapies in prostate adenocarcinoma: A systematic review and meta-analysis of 2244 patients in 12 randomized clinical trials. Clinical Genitourinary Cancer. 2011 | On topic SLR/MA/NMA |
| Parker et al. Overall survival benefit of radium-223 chloride (Alpharadin) in the treatment of patients with symptomatic bone metastases in Castration-resistant Prostate Cancer (CRPC): A phase III randomized trial (ALSYMPCA). European journal of cancer. 2011 | Mixed population |
| Okihara et al. Feasibility of tri-weekly docetaxel-based chemotherapy for elderly patients (age 75 and older) with castration-resistant prostate cancer. Urologia Internationalis. 2011 | Study design (non-RCT, opinion, commentary, single-arm, etc.) |
| Mark et al. 2011 | Study design (non-RCT, opinion, commentary, single-arm, etc.) |
| Kellokumpu-Lehtinen et al. Phase III, randomized, open-label study of triweekly versus biweekly docetaxel (T) as a treatment for advanced hormone-refractory prostate cancer (HRPC): Final analysis of the Finnish Uro-oncological Group Study 1-2003. Journal of clinical oncology. 2011 | Old conference abstracts (before 2019) |
| Hervonen et al. Phase III, randomized, open-label study of triweekly docetaxel versus biweekly docetaxel as treatments for advanced hormone-refractory prostate cancer: Findings from an interim safety analysis of the Finnish Uro-oncological Group Study 1-2003. Journal of clinical oncology. 2011 | Old conference abstracts (before 2019) |
| Caffo et al. Impact of docetaxel-based chemotherapy on quality of life of patients with castration-resistant prostate cancer: Results from a prospective phase II randomized trial. BJU International. 2011 | Mixed population |
| Azvolinsky. EMCC: Radium-223 plus chemo shows clear OS benefit. Oncology. 2011 | Study design (non-RCT, opinion, commentary, single-arm, etc.) |
| Anonymous. Abiraterone acetate (Zytiga) for metastatic castration-resistant prostate cancer. The Medical letter on drugs and therapeutics. 2011 | Study design (non-RCT, opinion, commentary, single-arm, etc.) |
| Colloca et al. Patient-reported outcomes after cytotoxic chemotherapy in metastatic castration-resistant prostate cancer: A systematic review. Cancer Treatment Reviews. 2010 | On topic SLR/MA/NMA |
| Moinpour et al. Do general dimensions of quality of life add clinical value to symptom data?. Journal of the National Cancer Institute. Monographs. 2007 | Study design (non-RCT, opinion, commentary, single-arm, etc.) |
| Collins et al. A systematic review and economic model of the clinical effectiveness and cost-effectiveness of docetaxel in combination with prednisone or prednisolone for the treatment of hormone-refractory metastatic prostate cancer. Health Technology Assessment. 2007 | On topic SLR/MA/NMA |
| Winquist et al. Non-hormonal systemic therapy in men with hormone-refractory prostate cancer and metastases: A systematic review from the Cancer Care Ontario Program in Evidence-based Care's Genitourinary Cancer Disease Site Group. BMC Cancer. 2006 | On topic SLR/MA/NMA |
| Shelley et al. Chemotherapy for hormone-refractory prostate cancer. Cochrane Database of Systematic Reviews. 2006 | On topic SLR/MA/NMA |
| Klatte et al. A randomized study of docetaxel and dexamethasone with low- or high-dose estramustine for patients with advanced hormone-refractory prostate cancer. BJU International. 2006 | Mixed population |
| Collins et al. A systematic review of the effectiveness of docetaxel and mitoxantrone for the treatment of metastatic hormone-refractory prostate cancer. British Journal of Cancer. 2006 | On topic SLR/MA/NMA |
| Sternberg et al. Does docetaxel plus prednisone prolong the survival of men with metastatic hormone-refractory prostate cancer?. Nature Clinical Practice Urology. 2005 | Study design (non-RCT, opinion, commentary, single-arm, etc.) |
| Sella et al. Can docetaxel plus estramustine prolong the survival of men with metastatic hormone-refractory prostate cancer?. Nature Clinical Practice Urology. 2005 | Study design (non-RCT, opinion, commentary, single-arm, etc.) |
| Oudard et al. Multi-centre randomised phase II study of two schedules of doxcetaxel, estramustine, and prednisone versus mitoxantrone plus prednisone in patients with metastatic hormone-refractory prostate cancer. Journal of clinical oncology. 2005 | Duplicate |
| Oudard, et al. Multicenter randomized phase II study of two schedules of docetaxel, estramustine, and prednisone versus mitoxantrone plus prednisone in patients with metastatic hormone-refractory prostate cancer. Journal of Clinical Oncology. 2005 | Mixed population |
| Montero et al. Docetaxel for treatment of solid tumours: A systematic review of clinical data. Lancet Oncology. 2005. 6:229-239 | On topic SLR/MA/NMA |
| Osobaet al. Health-related quality of life in men with metastatic prostate cancer treated with prednisone alone or mitoxantrone and prednisone. Journal of Clinical Oncology. 1999 | Population |
| Canfield et al. Sipuleucel-T for metastatic castration-resistant prostate cancer | Study design (non-RCT, opinion, commentary, single-arm, etc.) |
| Thiery-Vuillemin et al. An analysis of health-related quality of life in the phase III PROSELICA and FIRSTANA studies assessing cabazitaxel in patients with metastatic castration-resistant prostate cancer. ESMO Open. 2021 | Duplicate |
| Saad et al. Analysis of two poor prognosis subgroups in ACIS evaluating apalutamide + abiraterone acetate plus prednisone (APA + AAP) versus placebo (PBO) + AAP in metastatic castration-resistant prostate cancer (mCRPC). Journal of clinical oncology. 2021 | Duplicate |
| Saad et al. Apalutamide plus abiraterone acetate and prednisone versus placebo plus abiraterone and prednisone in metastatic, castration-resistant prostate cancer (ACIS): a randomised, placebo-controlled, double-blind, multinational, phase 3 study. The lancet. Oncology. 2021 | Duplicate |
| Hall et al. Association between disease indication and steroid use and mineralocorticoid-related toxicity of abiraterone acetate in patients with advanced prostate cancer: A meta-analysis of randomized control trials. Journal of Urology. 2021 | On topic SLR/MA/NMA |
| Xiong et al. Association of Novel Androgen Receptor Axis-Targeted Therapies With Diarrhea in Patients With Prostate Cancer: A Bayesian Network Analysis. Frontiers in medicine. 2021 | On topic SLR/MA/NMA |
| Annala et al. Cabazitaxel versus abiraterone or enzalutamide in poor prognosis metastatic castration-resistant prostate cancer: a multicentre, randomised, open-label, phase II trial. Annals of oncology : official journal of the european society for medical oncology. 2021 | Duplicate |
| Gillessen et al. Decreased fracture rate by mandating bone protecting agents in the EORTC 1333/PEACEIII trial combining Ra223 with enzalutamide versus enzalutamide alone: an updated safety analysis. Journal of clinical oncology. 2021 | Duplicate |
| Anonymous. Decreased Fracture Rate by Mandating Bone-Protecting Agents in the EORTC 1333/PEACE-3 Trial Combining Ra-223 With Enzalutamide Versus Enzalutamide Alone: An Updated Safety Analysis. Clinical Advances in Hematology and Oncology. 2021 | Study design |
| Caffo et al. Docetaxel and prednisone with or without enzalutamide as first-line treatment in patients with metastatic castration-resistant prostate cancer: CHEIRON, a randomised phase II trial. European journal of cancer (Oxford, England). 2021 | Duplicate |
| Wei et al. Efficacy and Safety of Abiraterone Acetate and Enzalutamide for the Treatment of Metastatic Castration-Resistant Prostate Cancer: A Systematic Review and Meta-Analysis. Frontiers in oncology. 2021 | On topic SLR/MA/NMA |
| Niazi et al. Efficacy of PARP Inhibitors as Maintenance Therapy for Metastatic Castration-Resistant Prostate Cancer: A Meta-Analysis of Randomized Controlled Trials. Oncology (Williston Park, N.Y.). 2021 | Population |
| Oudard et al. Health-related quality of life (HRQoL) in ACIS: A phase III trial of apalutamide with abiraterone acetate and prednisone (APA + AAP) vs AAP in metastatic castration-resistant prostate cancer (mCRPC). Annals of Oncology. 2021 | Duplicate |
| Sweeney et al. Ipatasertib plus abiraterone and prednisolone in metastatic castration-resistant prostate cancer (IPATential150): a multicentre, randomised, double-blind, phase 3 trial. Lancet (london, england). 2021 | Duplicate |
| Anonymous. ODENZA, a Prospective, Randomized, Open-Label, Multicenter, Cross-Over Phase 2 Trial of Preference Between Darolutamide and Enzalutamide in Men With Asymptomatic or Mildly Symptomatic Metastatic Castrate-Resistant Prostate Cancer. Clinical Advances in Hematology and Oncology. 2021 | Study design |
| Colomba et al. ODENZA: a French prospective, randomized, open-label, multicenter, cross-over phase II trial of preference between darolutamide and enzalutamide in men with asymptomatic or mildly symptomatic metastatic castrateresistant prostate cancer (CRPC). Journal of clinical oncology. 2021 | Duplicate |
| Maughan et al. Radium-223 plus Enzalutamide Versus Enzalutamide in Metastatic Castration-Refractory Prostate Cancer: final Safety and Efficacy Results. Oncologist. 2021 | Mixed population |
| Petrylak et al. A randomized phase IIa study of quantified bone scan response in patients with metastatic castration-resistant prostate cancer (mCRPC) treated with radium-223 dichloride alone or in combination with abiraterone acetate/prednisone or enzalutamide. ESMO open. 2021 | Mixed population |
| Sternberg et al. Safety analysis of the phase III IPATential150 trial of ipatasertib (ipat) plus abiraterone (abi) in patients with metastatic castration-resistant prostate cancer (mCRPC). Annals of Oncology. 2021 | Duplicate |
| Wang et al. The association of AR-V7 with resistance to Abiraterone in metastatic castration-resistant prostate cancer. *Journal of men's health.* 2022 | Study design (non-RCT, opinion, commentary, single-arm, etc) |
| Salgadoet al. Evaluation of maintenance of the common androgen deprivation therapy with the new antiandrogen therapy in patients with castration-resistant prostate cancer: a systematic review. *International urology and nephrology.* 2022 | On topic SLR/MA/NMA |
| Saad et al. PROpel: Phase III trial of olaparib (ola) and abiraterone (abi) versus placebo (pbo) and abi as first-line (1L) therapy for patients (pts) with metastatic castration-resistant prostate cancer (mCRPC). *Journal of Clinical Oncology.* 2022 | Duplicate |
| Rizzo et al. Incidence of grade 3-4 adverse events, dose reduction, and treatment discontinuation in castration-resistant prostate cancer patients receiving PARP inhibitors: a meta-analysis. *Expert opinion on drug metabolism & toxicology.* 2022 | On topic SLR/MA/NMA |
| Ohlmann et al. LHRH sparing therapy in patients with chemotherapy-naive, mCRPC treated with abiraterone acetate plus prednisone: results of the randomized phase II SPARE trial. *Prostate cancer and prostatic diseases*. 2022 | Duplicate |
| Lehtonen et al. 2-weekly versus 3-weekly docetaxel for metastatic castration-resistant prostate cancer: complete quality of life results from the randomised, phase-III PROSTY trial. *Acta oncologica (Stockholm, Sweden).* 2022 | Duplicate |
| Indrawan et al. P35-4 Corticosteroid switch in metastatic castration-resistant prostate cancer treated with abiraterone: A systematic review. *Annals of Oncology.* 2022 | On topic SLR/MA/NMA |
| Gutta et al. CO101 A Systematic Literature Review of Randomized Controlled Trials Reporting Efficacy and Safety of Treatments in Patients with Advanced Prostate Cancer in Asian Countries. *Value in Health.* 2022 | On topic SLR/MA/NMA |
| Fallara et al. Androgen annihilation versus advanced androgen blockage as first line treatment for metastatic castration resistant prostate cancer: A systematic review and meta-analysis. *Critical reviews in oncology/hematology.* 2022 | On topic SLR/MA/NMA |
| Chi et al. Phase 3 MAGNITUDE study: First results of niraparib (NIRA) with abiraterone acetate and prednisone (AAP) as first-line therapy in patients (pts) with metastatic castration-resistant prostate cancer (mCRPC) with and without homologous recombination repair (HRR) gene alterations. *Journal of Clinical Oncology.* 2022 | Duplicate |
| Baciarello et al. Patient Preference Between Cabazitaxel and Docetaxel for First-line Chemotherapy in Metastatic Castration-resistant Prostate Cancer: the CABADOC Trial. *European urology.* 2022 | Population |
| Ahmed Naqvi et al. Differential efficacy of PARP inhibitors in metastatic castration-resistant prostate cancer with DNA repair defects: A systematic review and meta-analysis. *Journal of Clinical Oncology.* 2022 | On topic SLR/MA/NMA |

1. Treatment Regimens

Table 4: Treatment Regimen Abbreviations

| **Abbreviation** | **Name** |
| --- | --- |
| AA+DEX | Abiraterone acetate 1000mg PO QD + Dexamethasone 0.5mg PO QD |
| AAP | Abiraterone acetate PO QD 1000mg + Prednisone/Prednisolone 5mg PO BID/10mg PO QD |
| AAP2.5BID | Abiraterone acetate PO QD 1000mg + Prednisone 2.5mg BID |
| AAP5QD | Abiraterone acetate 1000mg PO QD + Prednisone 5mg PO QD |
| AAP-AAF | Abiraterone acetate AAF 500mg PO QD + Methylprednisolone 4mg PO BID |
| AAP-G | Abiraterone acetate 300mg (generic tablet) PO QD + Prednisone 5mg PO BID |
| AAPnoLHRH | Abiraterone acetate 1000 mg PO QD + Prednisone 5 mg PO BID with no LHRH therapy |
| AAPorENZA | Abiraterone acetate 1000mg PO QD + Prednisone 5mg PO BID or Enzalutamide 160mg PO QD |
| APA+AAP | Apalutamide 240mg PO QD + Abiraterone acetate 1000mg PO QD + Prednisone 5mg PO BID |
| BIC | Bicalutamide 50mg PO QD |
| BSC | Best supportive care (Placebo, Prednisone 5mg PO BID, Hydrocortisone 40mg PO QD, and Prednisolone 5mg PO BID) |
| CABA20 | Cabazitaxel 20mg/m^2^ IV Q3W + Prednisone 10mg PO QD |
| CABA25+AAP | Cabazitaxel 25mg/m^2^ IV Q3W + Abiraterone Acetate 1000mg PO QD + Prednisone 5mg PO BID |
| CABA25+PS | Cabazitaxel 25mg/m^2^ IV Q3W + Prednisone 5mg PO BID/10mg PO QD |
| DOC30+PS | Docetaxel 30mg/m^2^ IV Q1W + Prednisone/Prednisolone 5mg PO BID |
| DOC50+PL10 | Docetaxel 50mg/m^2^ IV Q2W + Prednisolone 10mg PO QD |
| DOC75+PS | Docetaxel 75mg/m^2^ IV Q3W + Prednisone/Prednisolone 5mg PO BID/10mg PO QD |
| ENZA | Enzalutamide 160mg PO QD |
| ENZA+AAP | Enzalutamide 160mg PO QD + Abiraterone acetate PO QD 1000mg + Prednisone 5mg BID |
| IPA+AAP | Ipatasertib 400mg PO QD + Abiraterone acetate 1000mg PO QD + Prednisone/Prednisolone 5mg PO BID |
| MIT12+PS | Mitoxantrone 12mg/m^2^ IV Q3W + Prednisone/Prednisolone 5mg PO BID/10mg PO QD |
| MIT14+HC | Mitoxantrone 14mg/m^2^ IV Q3W + Hydrocortisone 40mg PO QD |
| NIRA+AAP | Niraparib 200mg PO QD + Abiraterone acetate 1000mg PO QD + Prednisone 10mg PO QD |
| OLAP | Olaparib 300mg PO BID |
| OLAP+AAP | Olaparib 300mg PO BID + Abiraterone acetate 1000mg PO QD + Prednisone/Prednisolone 5 mg PO BID |
| Ra50+ENZA | Radium-223 50 kBq/kg Q4W + Enzalutamide 160mg PO QD |
| Ra55+AAP | Radium-223 55 kBq/kg Q4W + Abiraterone acetate 1000mg PO QD + Prednisone/Prednisolone 5mg PO BID |
| SIP-T | Sipuleucel-T (1 infusion every 2 weeks for 6 weeks) |
| TALA+ENZA | Talazoparib 0.5mg QD + Enzalutamide 160mg PO QD |

Abbreviations: BID **=** twice daily; IV = intravenous; PO = orally; Q1W = weekly; Q2W = every two weeks; Q3W = every three weeks; Q4W = every four weeks; QD = daily.

1. Evidence Networks

Figure 2: Evidence Network for rPFS


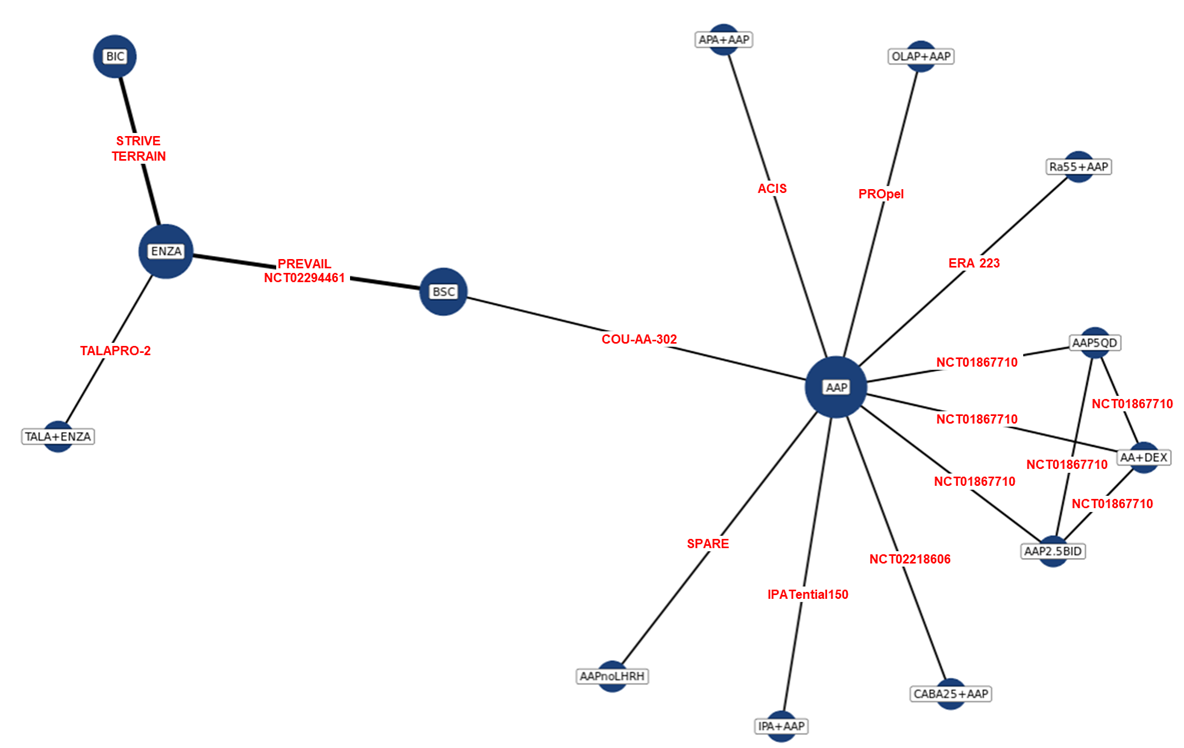


Abbreviations: rPFS = radiographic progression-free survival

Note: for full list of treatment regimens refer to **Appendix B**.

Figure 3: Evidence Network for OS


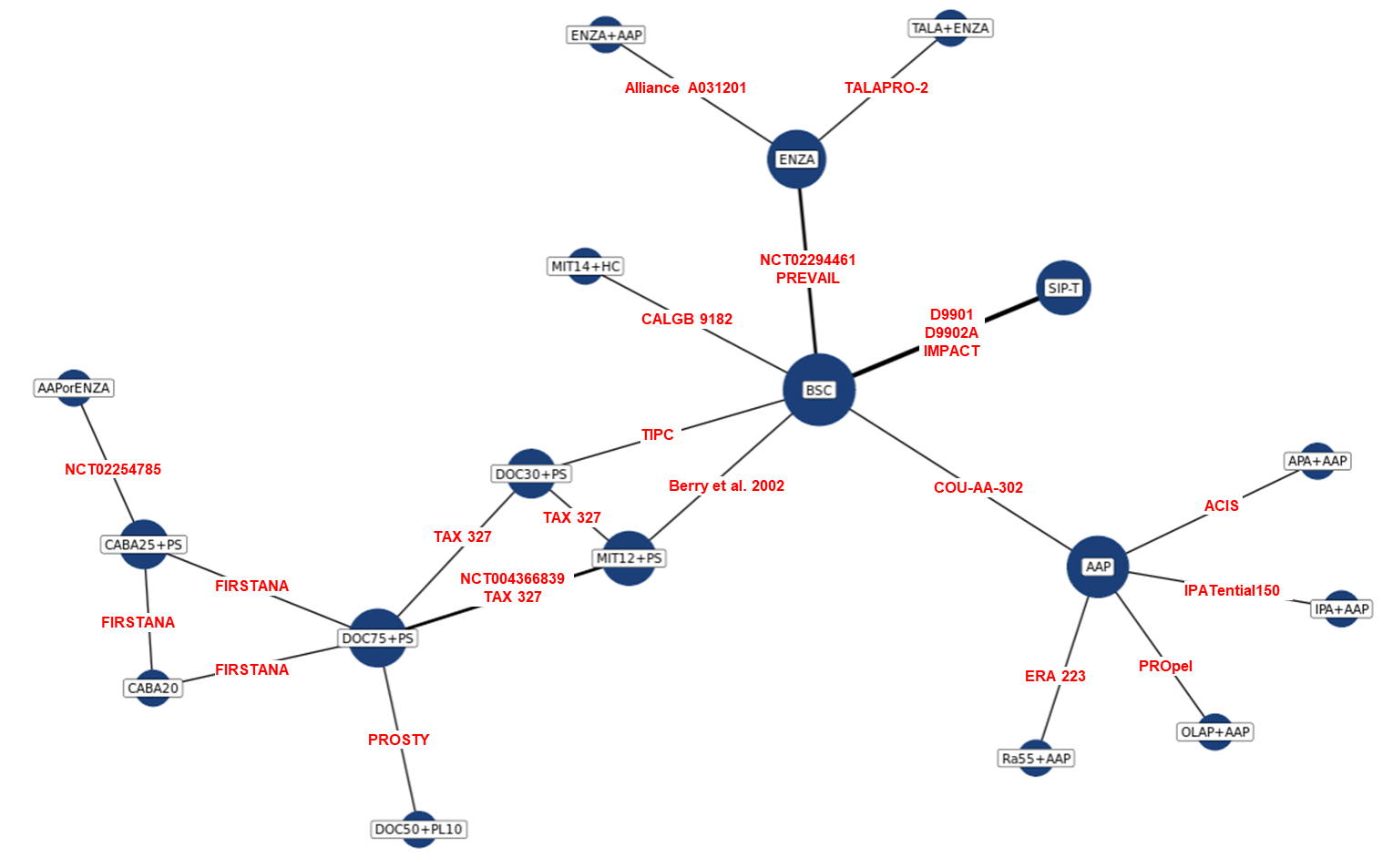


Abbreviations: OS = overall survival

Note: for full list of treatment regimens refer to **Appendix B**.

Figure 4: Evidence Network for Time to PSA Progression


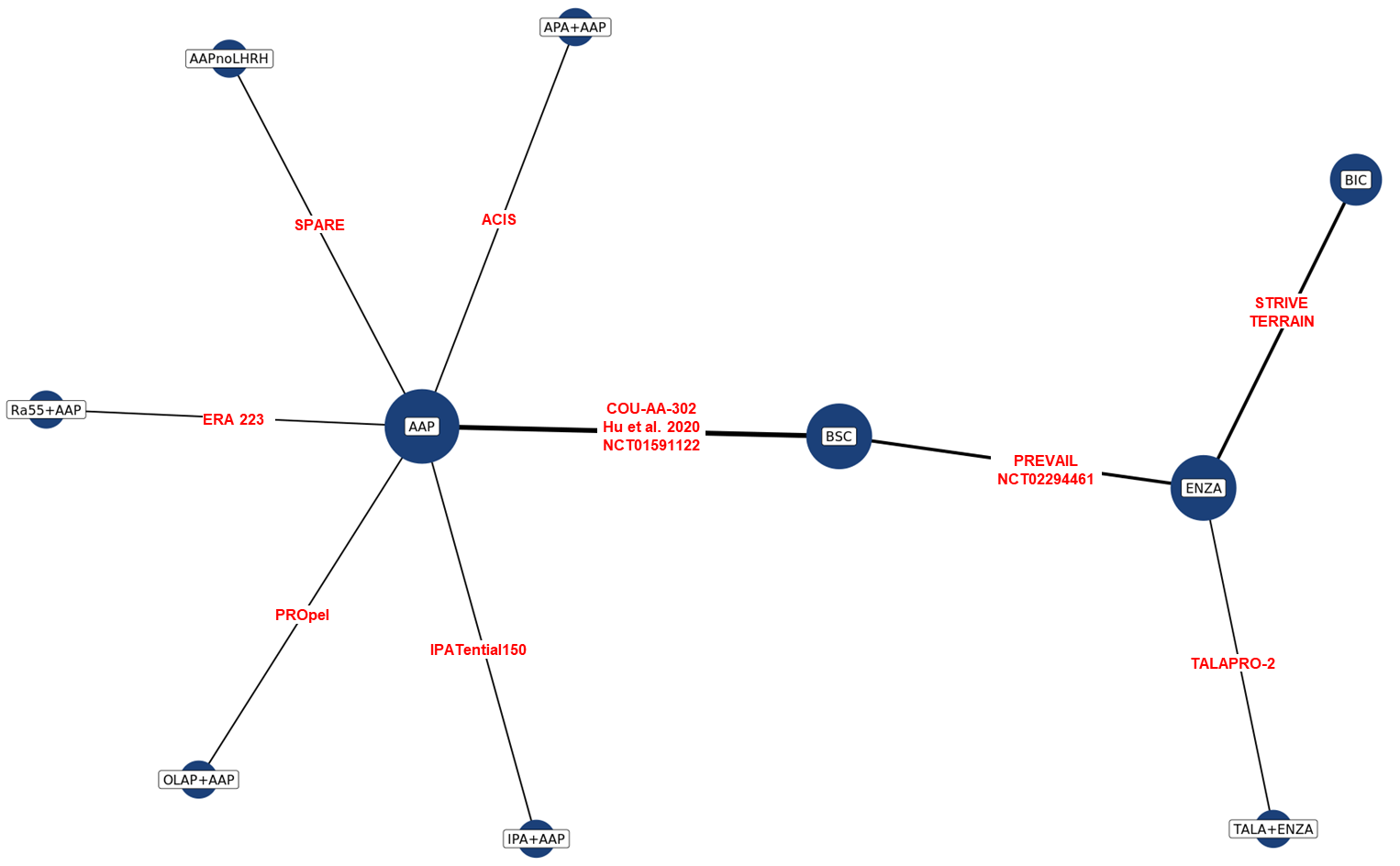


Abbreviations: PSA = prostate specific antigen

Note: for full list of treatment regimens refer to **Appendix B**.

Figure 5: Evidence Network for Time to Cytotoxic Chemotherapy Initiation


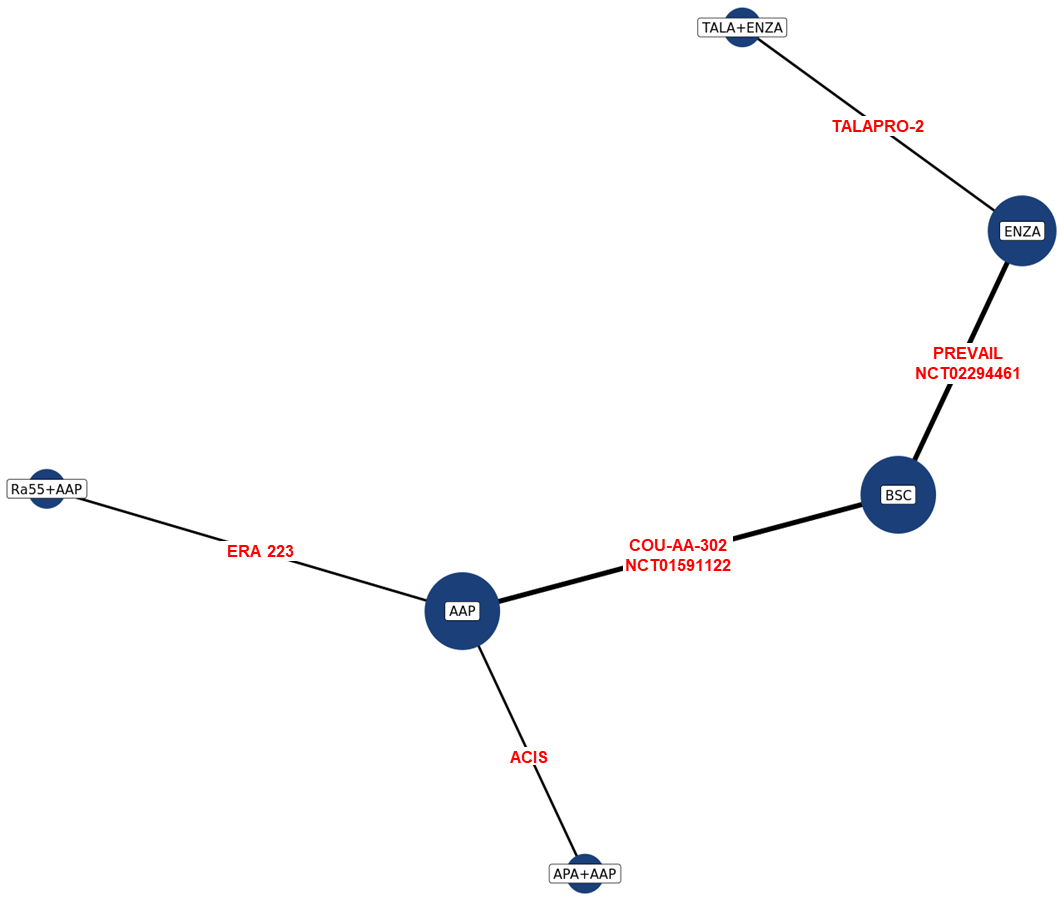


Note: for full list of treatment regimens refer to **Appendix B**.

Figure 6: Evidence Network for PSA Response


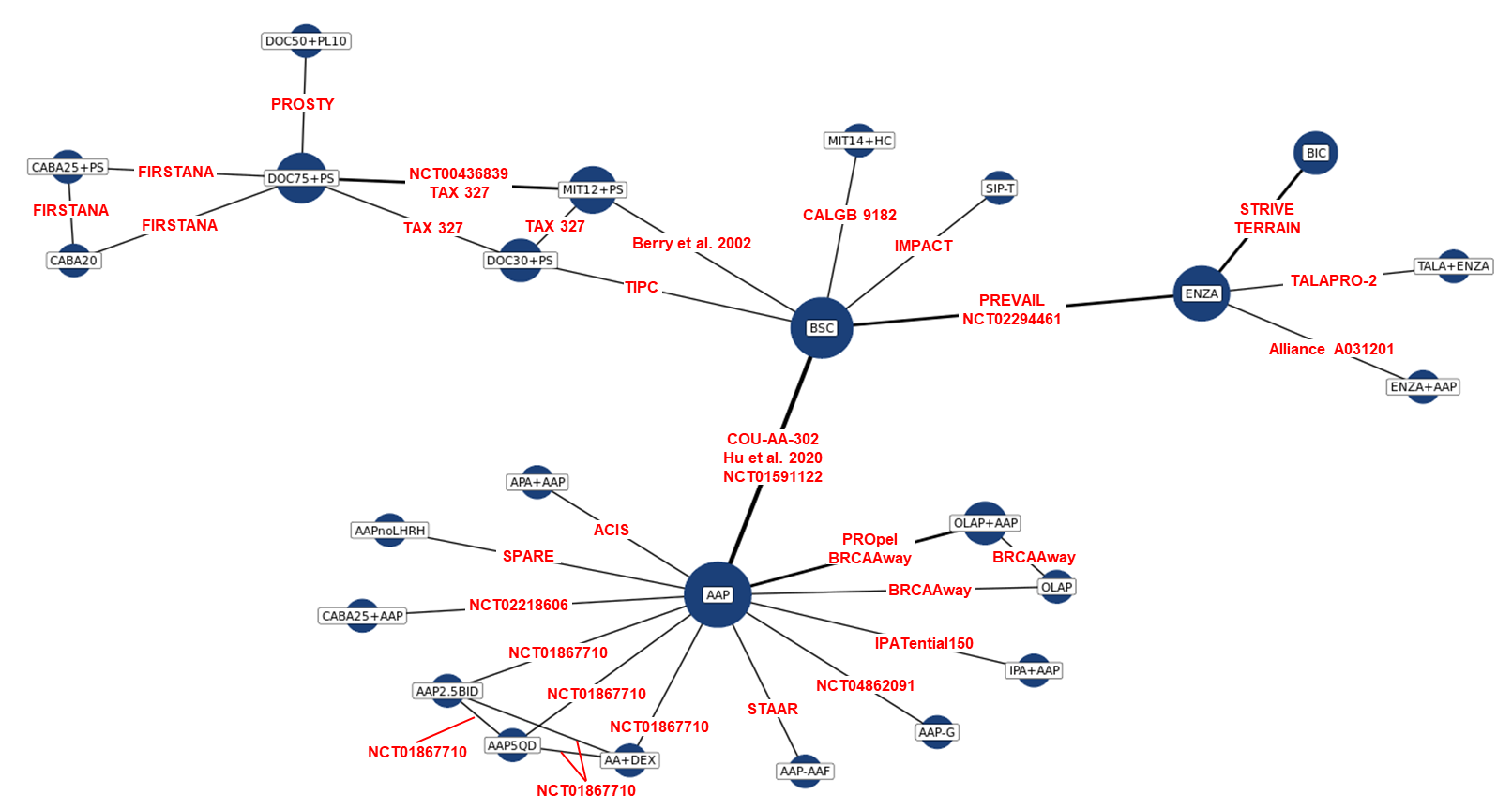


Abbreviations: PSA = prostate specific antigen

Note: for full list of treatment regimens refer to **Appendix B**.

Figure 7: Evidence Network for ORR


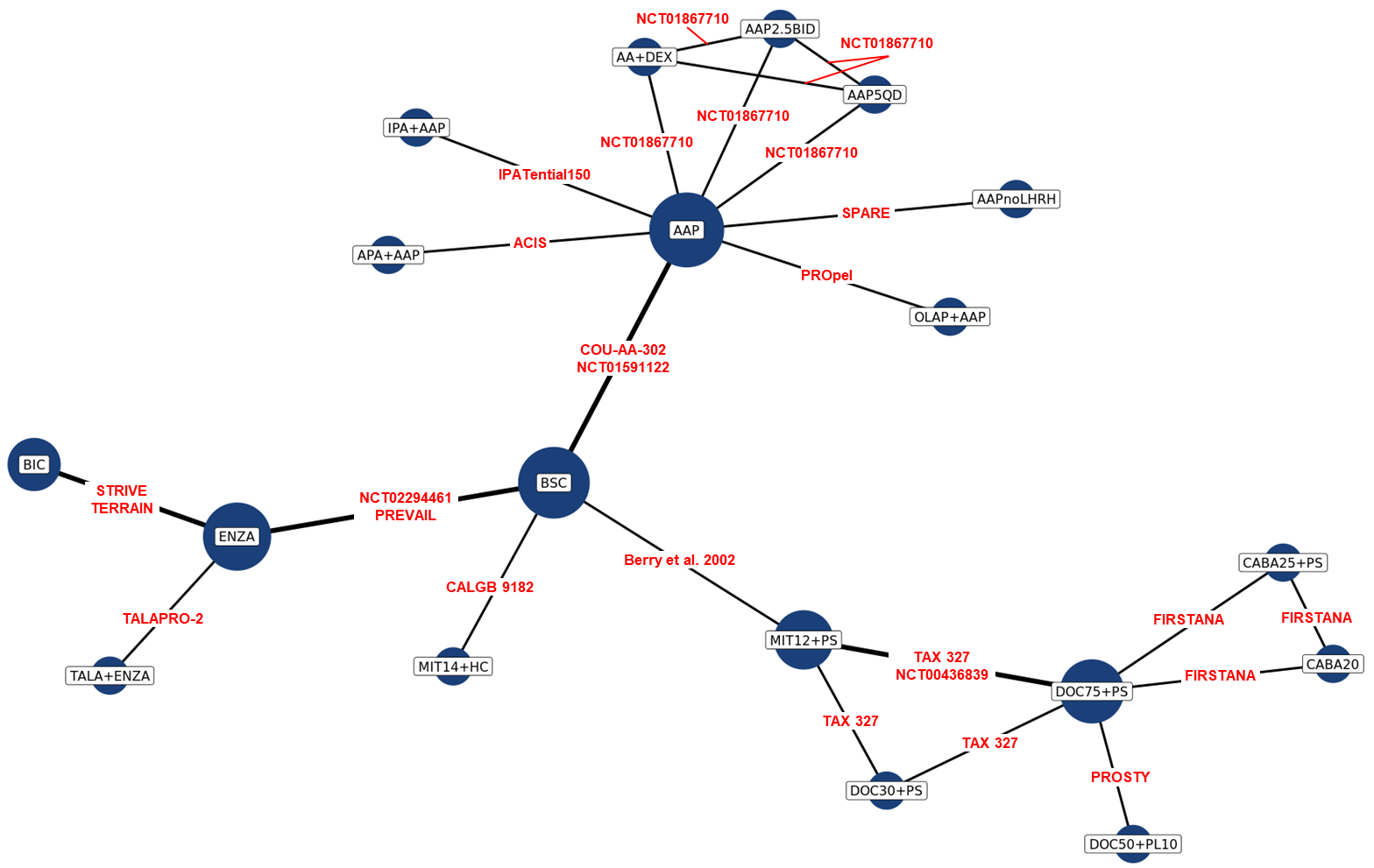


Abbreviations: ORR = objective response rate

Note: for full list of treatment regimens refer to **Appendix B**.

Figure 8: Evidence Network for Grade ≥3 Anemia


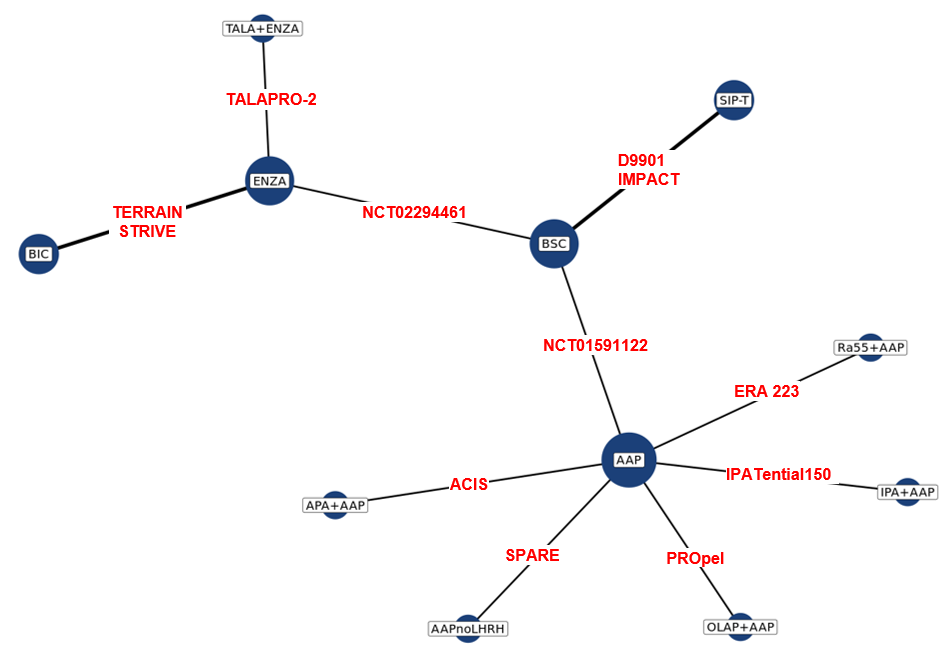


Note: for full list of treatment regimens refer to **Appendix B**.

Figure 9: Evidence Network for All Grade Asthenia


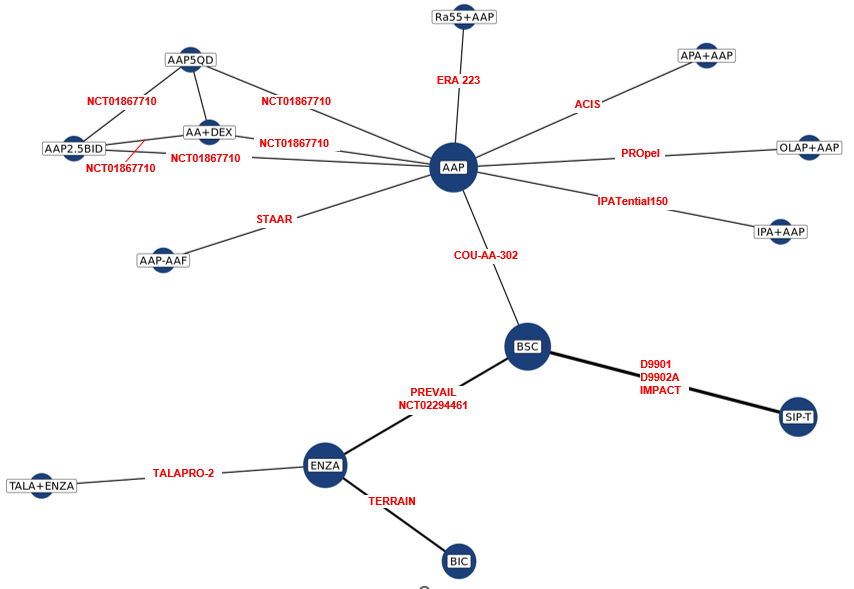


Note: for full list of treatment regimens refer to **Appendix B**.

Figure 10: Evidence Network for Grade ≥3 Asthenia


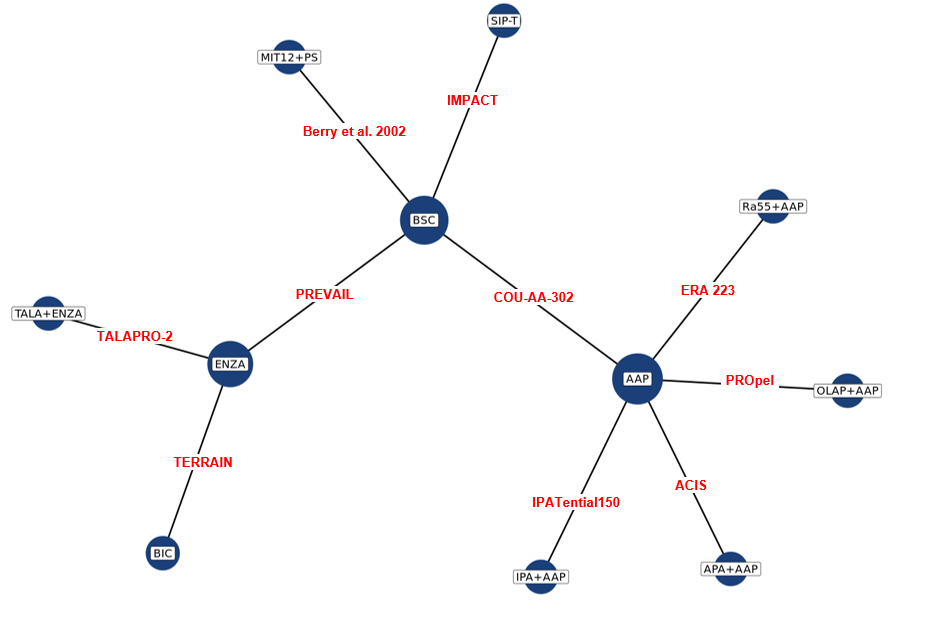


Note: for full list of treatment regimens refer to **Appendix B**.

Figure 11: Evidence Network for All Grade Fatigue


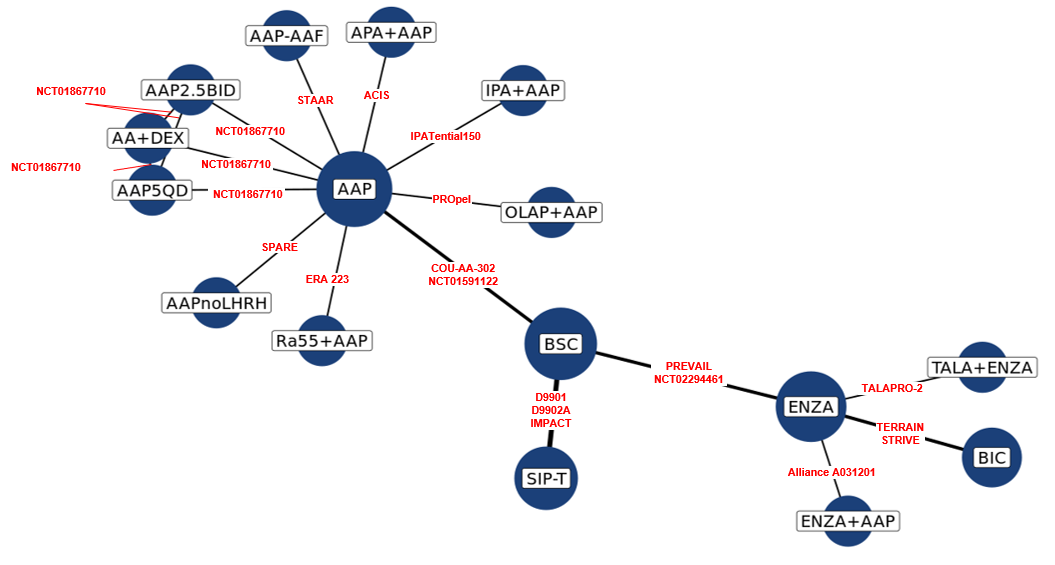


Note: for full list of treatment regimens refer to **Appendix B**.

Figure 12: Evidence Network for Grade ≥3 Fatigue


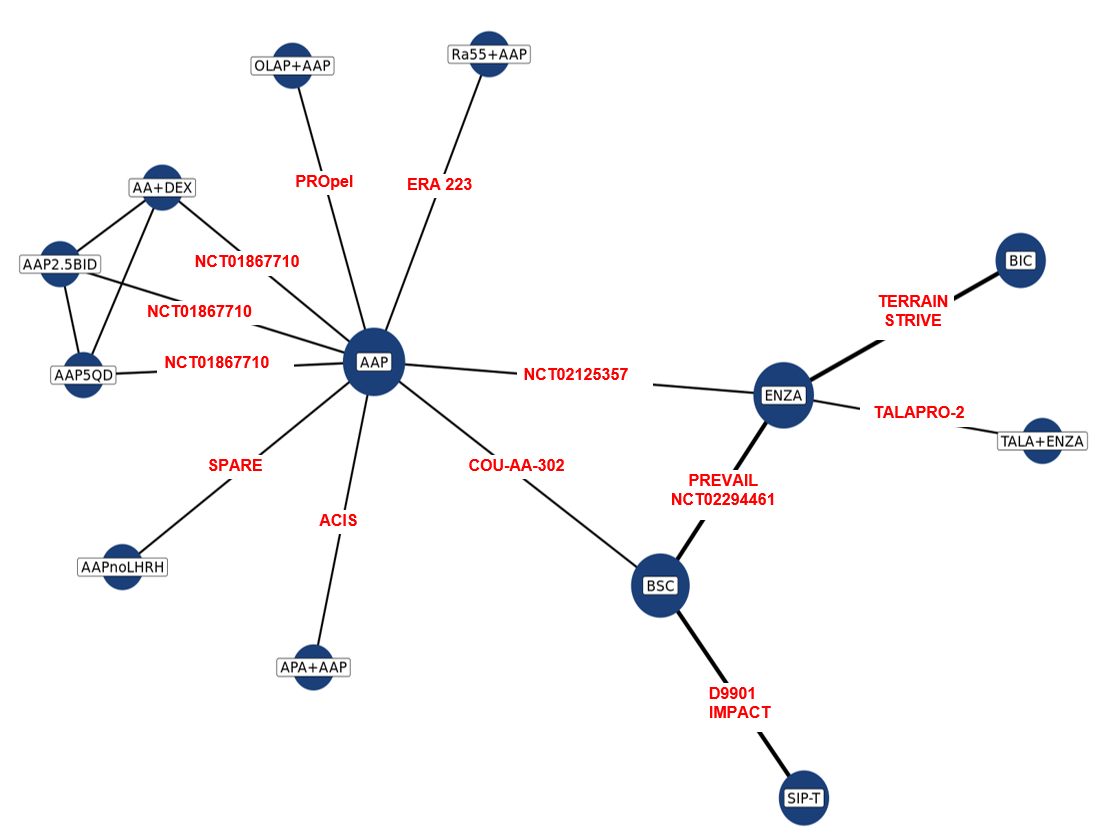


Note: for full list of treatment regimens refer to **Appendix B**.

Figure 13: Evidence Network for All Grade Nausea


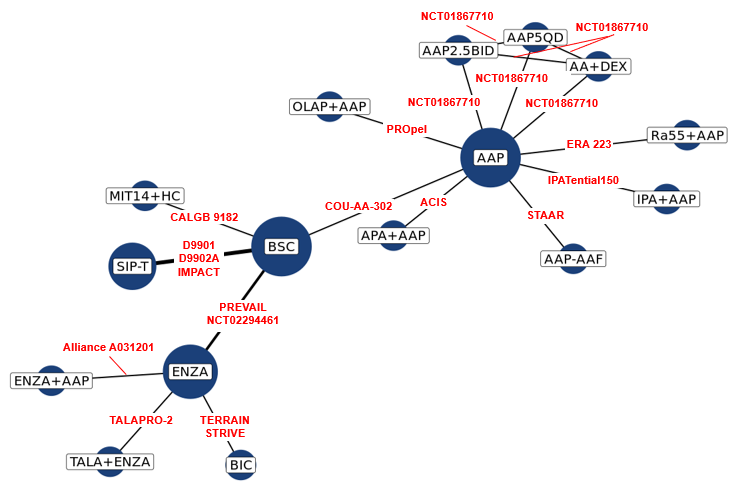


Note: for full list of treatment regimens refer to **Appendix B**

Figure 14: Evidence Network for Grade ≥3 Nausea


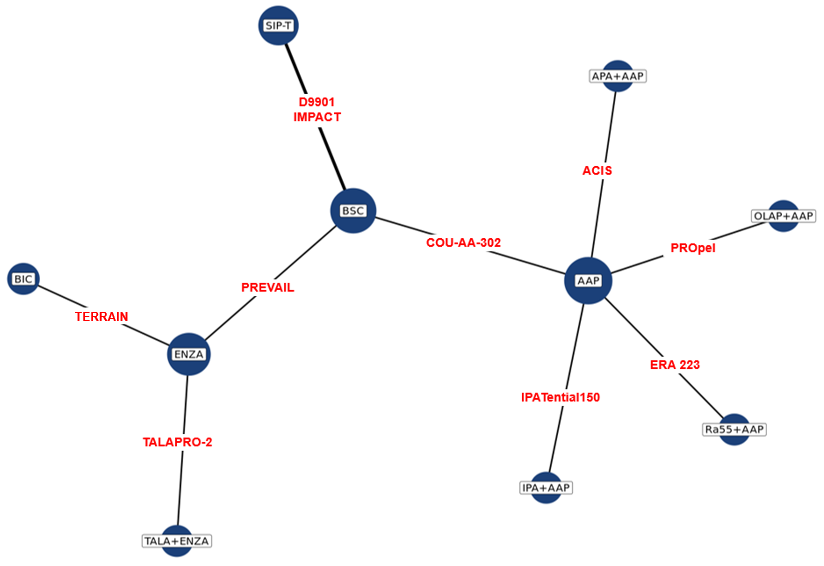


Note: for full list of treatment regimens refer to **Appendix B**

Figure 15: Evidence Network for All Grade Decreased Appetite


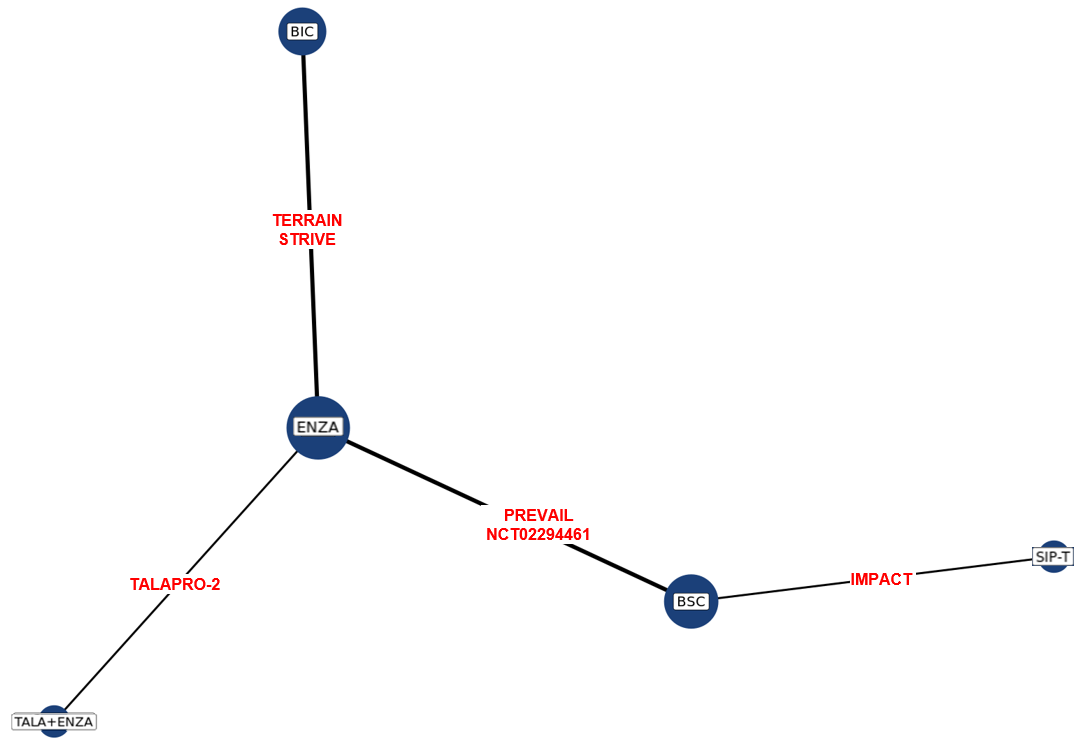


Note: for full list of treatment regimens refer to **Appendix B**.

Figure 16: Evidence Network for Grade ≥3 Decreased Appetite


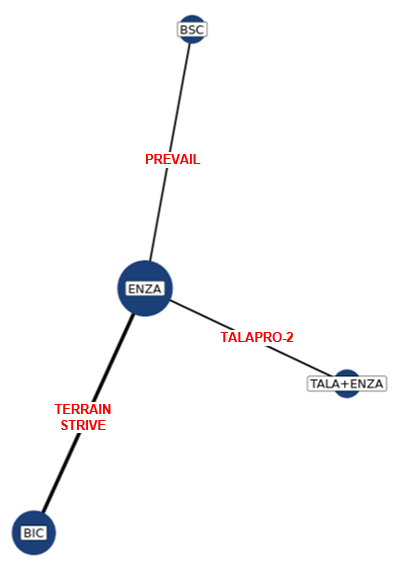


Note: for full list of treatment regimens refer to **Appendix B**.

1. NMA Assumptions

**Network Meta-analysis Assumptions**

To enable comparison of treatments via a connected network, several assumptions were made; these assumptions were supported by clinical opinion and similar to a previously published NMA.[^11^](#_ENREF_11) First, trials were excluded if they were disconnected from the main network or if reported medians for time to event outcomes had no associated HR or KM curve. The main network was defined as one where the TALAPRO-2 trial (talazoparib plus enzalutamide) was included. Second, placebo and corticosteroid-only arms of clinical trials were combined into a single “best supportive care” (BSC) node and assumed equivalent relative to the outcomes under consideration. Third, prednisone and prednisolone at the same dose were assumed to be comparable corticosteroids, e.g., docetaxel 75 mg/m^2^ intravenously (IV) every three weeks (Q3W) + prednisone 10 mg orally (PO) daily (QD) and docetaxel 75 mg/m^2^ IV Q3W + prednisolone 10 mg PO QD were aggregated into one node. Forth, prednisone 5 mg twice daily (BID) and prednisone 10 mg QD were assumed to be comparable doses, e.g., abiraterone acetate PO QD 1000 mg + prednisone 5 mg BID and abiraterone acetate 1000 mg PO QD + prednisone 10 mg PO QD were aggregated into one node. Lastly, prednisone 2.5 mg BID and prednisone 5 mg QD were assumed to be comparable doses.

1. PRISMA Diagrams

Figure 17: Original PRISMA Diagram


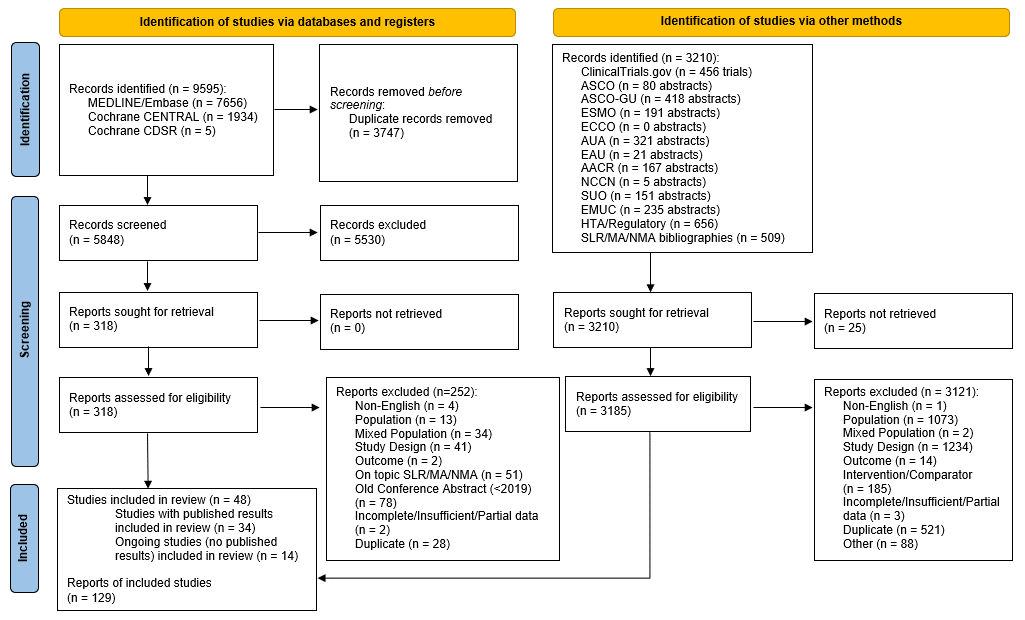


Abbreviations: AACR = American Association for Cancer Research; ASCO = American Society of Clinical Oncology; ASCO-GU = American Society of Clinical Oncology – Genitourinary Cancers Symposium; AUA = American Urological Association; EAU = European Association of Urology; ECCO = European CanCer Organization; EMUC = European Multidisciplinary Congress on Urological Cancers; ESMO = European Society for Medical Oncology; HTA = Health Technology Assessment; MA = Meta-analysis; NCCN = National Comprehensive Cancer Network; NMA = Network meta-analysis; RCTs = Randomized controlled trials; SLR = systematic literature review; SUO = Society of Urologic Oncology

Source*:* Page et al., 2021[^12^](#_ENREF_12)

Figure 18: February 2022 Updated PRISMA Diagram


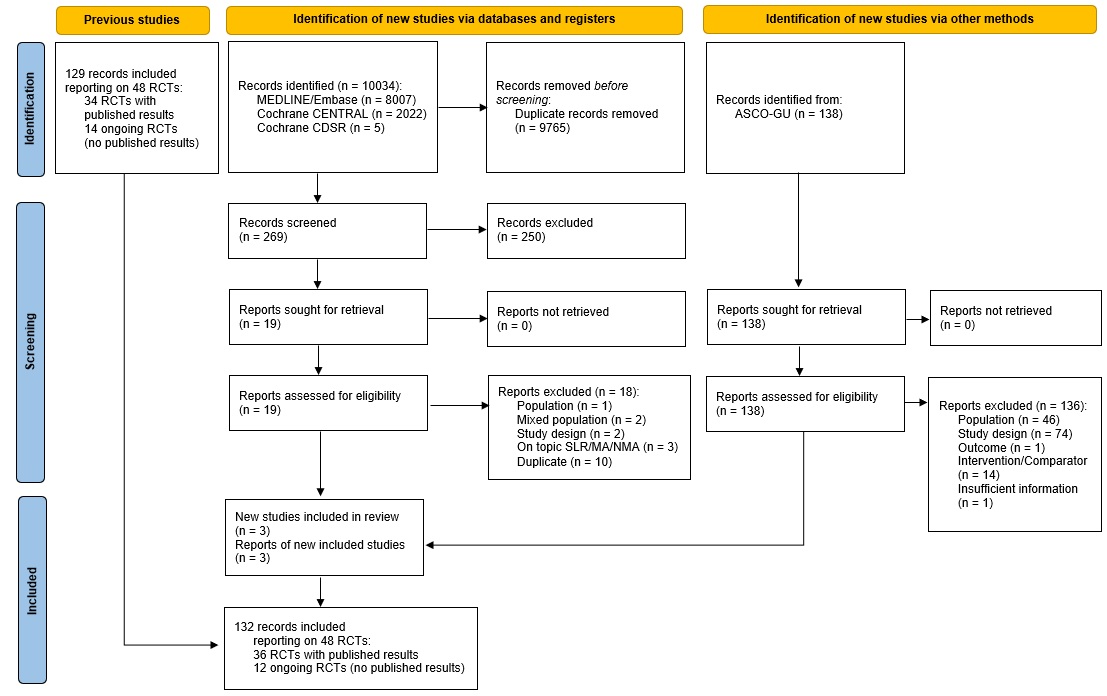


Abbreviations: ASCO-GU = American Society of Clinical Oncology – Genitourinary Cancers Symposium; MA = Meta-analysis; NMA = Network meta-analysis; RCTs = Randomized controlled trials; SLR = Systematic Literature Review

Source*:* Page et al., 2021^16^

Figure 19: October 2022 Updated PRISMA Diagram


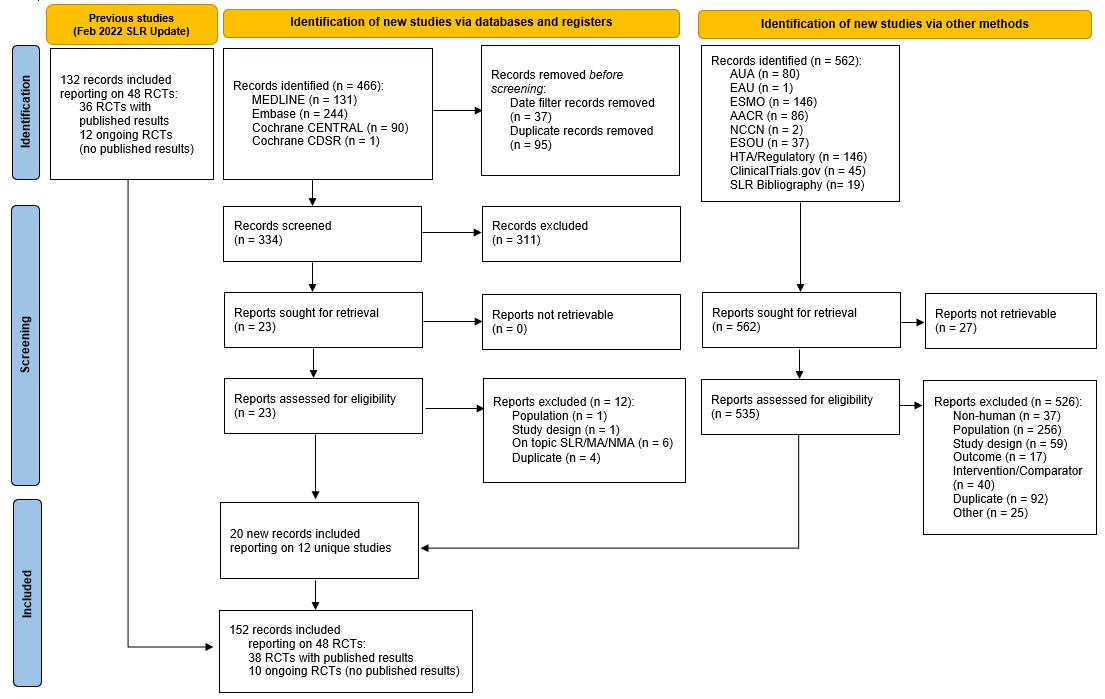


Abbreviations: ASCO-GU = American Society of Clinical Oncology – Genitourinary Cancers Symposium; MA = Meta-analysis; NMA = Network meta-analysis; RCTs = Randomized controlled trials; SLR = systematic literature review

Source*:* Page et al., 2021^16^

1. Study Quality Assessment

Table 5: Assessment of Study Quality

| **Reference** | **Trial; NCT** | **Was randomization carried out appropriately?** | **Was the concealment of treatment allocation adequate?** | **Were the groups similar at the outset of the study in terms of prognostic factors?** | **Were the care providers, participants, and the outcome assessors blind to treatment allocation?** | **Were there any unexpected imbalances in drop-outs between groups?** | **Is there any evidence to suggest that the authors measured more outcomes than they reported?** | **Did the analysis include an ITT analysis? If so, was this appropriate and were appropriate methods used to account for missing data?** |
| --- | --- | --- | --- | --- | --- | --- | --- | --- |
|  |  |  |  |  |  |  |  |  |
| Ryan-2013[^13^](#_ENREF_13) | COU-AA-302; NCT00887198 | Yes | Not clear | Yes | Not clear | Not clear | No | Yes |
| Ye-2017[^14^](#_ENREF_14) | NCT01591122 | Not clear | Not clear | Yes | Not clear | No | No | Yes |
| Attard-2019[^15^](#_ENREF_15) | NCT01867710 | Yes | Yes | Yes | No | No | No | Yes |
| Stein-2018[^16^](#_ENREF_16) | STAAR; NCT02737332 | Yes | Not clear | Yes | No | No | No | Yes |
| Beer-2014[^17^](#_ENREF_17) | PREVAIL; NCT01212991 | Yes | Yes | Yes | Yes | Not clear | No | Yes |
| Shore-2016[^18^](#_ENREF_18) | TERRAIN; NCT01288911 | Yes | Not clear | Yes | Yes | No | No | Yes |
| Penson-2016[^19^](#_ENREF_19) | STRIVE; NCT01664923 | Yes | Not clear | Yes | Not clear | Yes | No | Yes |
| Khalaf-2019[^20^](#_ENREF_20) | NCT02125357 | Yes | Not clear | Yes | No | No | No | Yes |
| Saad-2021[^21^](#_ENREF_21) | ACIS; NCT02257736 | Yes | Yes | Yes | Yes | No | No | Yes |
| Kantoff-1999[^22^](#_ENREF_22) | CALGB 9182 | Not clear | Not clear | Yes | No | Not clear | No | Not clear |
| Berry-2002[^23^](#_ENREF_23) | NR | Not clear | Not clear | Yes | No | Not clear | No | Yes |
| De Wit-2004[^24^](#_ENREF_24) | TAX 327 | Yes | Yes | Yes | No | Yes | No | Yes |
| Zhou-2015[^25^](#_ENREF_25) | NCT00436839 | Yes | Yes | Yes | No | No | No | Yes |
| Caffo-2015[^26^](#_ENREF_26) | EudraCT: 2006-005728-17 | Yes | Yes | Yes | No | Yes | No | No |
| Kellokumpu-Lehtinen-2013[^27^](#_ENREF_27) | PROSTY; NCT00255606 | Yes | Not clear | Yes | No | No | No | No |
| Cash-2018[^28^](#_ENREF_28) | PRINCE | Yes | No | Yes | No | No | No | No |
| Fossa-2007[^29^](#_ENREF_29) | TIPC | Yes | Not clear | No | Not clear | Not clear | No | No |
| Oudard-2017[^30^](#_ENREF_30) | FIRSTANA; NCT01308567 | Yes | Not clear | Yes | No | No | No | Yes |
| Annala-2021[^31^](#_ENREF_31) | NCT02254785 | Yes | No | Yes | No | No | No | Yes |
| Small-2006[^32^](#_ENREF_32) | D9901; NCT00005947 | Yes | Yes | Yes | Yes | No | No | Yes |
| Higano-2009[^33^](#_ENREF_33) | D9902A; NCT01133704 | Not clear | Not clear | Yes | Yes | Yes | No | Yes |
| Kantoff-2010[^34^](#_ENREF_34) | IMPACT; NCT00065442 | Not clear | Not clear | Yes | Yes | No | No | Yes |
| Small-2015[^35^](#_ENREF_35) | STAMP; NCT01487863 | Not clear | Not clear | Yes | No | No | No | Yes |
| Parker-2013[^36^](#_ENREF_36) | NCT00337155 | Not clear | Not clear | Yes | Not clear | No | No | No |
| Smith-2019[^37^](#_ENREF_37) | ERA 223; NCT02043678 | Yes | Not clear | Yes | Not clear | No | No | Yes |
| Sweeney-2021[^38^](#_ENREF_38) | IPATential150; NCT03072238 | Yes | Yes | Yes | Yes | No | No | Yes |
| Pu-2022[^39^](#_ENREF_39) | NCT02294461 | Yes | Not clear | Yes | Yes | Yes | No | Yes |
| Ohlmann-2022[^40^](#_ENREF_40) | SPARE;  NCT02077634 | Yes | No | Yes | No | No | Yes | Yes |
| Clarke-2022[^41^](#_ENREF_41) | PROpel; NCT03732820 | Yes | Not clear | Yes | Yes | Not clear | No | Yes |
| Agarwal-2023[^9^](#_ENREF_9) | TALAPRO-2; NCT03395197 | Yes | Yes | Yes | Yes | No | No | Yes |

1. Feasibility Assessment

There were instances where trials were excluded from specific outcome networks; however, these trials remained in other outcome networks and were therefore included in the total of 33 trials. Three trials were excluded from the time to PSA progression network[^20^](#_ENREF_20)^,^[^23^](#_ENREF_23)^,^[^30^](#_ENREF_30) and two trials were excluded from the PSA response network[^20^](#_ENREF_20)^,^[^37^](#_ENREF_37) on the basis of having a broader outcome definition for these endpoints, which were considered significantly different than the majority of the studies. Certain trials were also excluded due to only reporting median values with no associated HR or KM curve required to conduct an NMA, including two trials for OS[^42^](#_ENREF_42)^,^[^43^](#_ENREF_43), two trials for time to PSA progression[^15^](#_ENREF_15)^,^[^43^](#_ENREF_43), and one trial for time to initiation of cytotoxic chemotherapy.[^34^](#_ENREF_34)

1. Trial Characteristics

Table 6: Included Trial Characteristics

| **Trial; NCT** | | **Phase** | **Study Design** | **Blinded** | **Setting** | **Treatment arms** | | **N randomized** | **Study Follow-up Time** |
| --- | --- | --- | --- | --- | --- | --- | --- | --- | --- |
| **TALAPRO-2;**  **NCT03395197** | | 3 | - Randomized - Parallel Assignment - Comparative | Yes | Multicenter | Talazoparib 0.5 mg  Enzalutamide 160mg | | 402 | Median follow-up: 28 months |
|  |  |  |  |  |  | Placebo Enzalutamide 160mg | | 403 | Median follow-up: 27.1 months |
| **COU-AA-302;**  **NCT00887198** | | 3 | - Randomized - Parallel Assignment - Comparative | Yes | Multicenter | Abiraterone acetate 1000 mg daily  Prednisone 5 mg twice daily | | 546 | Median follow-up: 22.2 months |
|  |  |  |  |  |  | Placebo  Prednisone 5 mg twice daily | | 542 |  |
| **NCT01591122** | | 3 | - Randomized - Parallel Assignment - Comparative | Yes | Multicenter | Abiraterone acetate 1000 mg once daily Prednisone 5 mg twice daily | | 157 | NR |
|  |  |  |  |  |  | Placebo  Prednisone 5 mg twice daily | | 156 |  |
| **NCT01867710** | | 2 | - Randomized - Parallel Assignment - Non-comparative | No | Multicenter | Abiraterone acetate 1000 mg once daily  Prednisone 5 mg twice daily | | 41 | Maximum follow-up time on treatment of 35.6 months |
|  |  |  |  |  |  | Abiraterone acetate 1000 mg once daily  Prednisone 5 mg once daily | | 41 |  |
|  |  |  |  |  |  | Abiraterone acetate 1000 mg once daily  Prednisone 2.5 mg twice daily | | 40 |  |
|  |  |  |  |  |  | Abiraterone acetate 1000 mg once daily  Dexamethasone 0.5 mg once daily | | 42 |  |
| **STAAR;**  **NCT02737332** | | 2 | - Randomized - Parallel Assignment - Comparative | No | Multicenter | Abiraterone acetate (fine particle) 500 mg daily  Methylprednisolone 4 mg twice daily | | 24 | NR |
|  |  |  |  |  |  | Abiraterone acetate (originator) 1000 mg daily  Prednisone 5 mg twice daily | | 29 |  |
| **Hu et al. 2020** | | 3 | - Randomized - Parallel Assignment - Comparative | Yes | Multicenter | Abiraterone acetate 1000 mg once daily  Prednisone 5 mg twice daily | | 178 | Median follow-up of 22.8 months |
|  |  |  |  |  |  | Placebo  Prednisone 5 mg twice daily | | 84 |  |
| **PREVAIL;**  **NCT01212991** | | 3 | - Randomized - Parallel Assignment - Comparative | Yes | Multicenter | Enzalutamide 160 mg once daily | | 872 | NR |
|  |  |  |  |  |  | Placebo | | 845 |  |
| **NCT02294461** | | 3 | - Randomized - Parallel Assignment - Comparative | Yes | Multicenter | Enzalutamide 160 mg | | 198 | Median follow-up: 7.33 months |
|  |  |  |  |  |  | Placebo | | 190 | Median follow-up: 3.02 months |
| **TERRAIN;**  **NCT01288911** | | 2 | - Randomized - Parallel Assignment - Comparative | Yes | Multicenter | Enzalutamide 160 mg | | 184 | Median: 20.0 months  (IQR: 15.0-25.6) |
|  |  |  |  |  |  | Bicalutamide 50 mg | | 191 | Median: 16.7 months  (IQR: 10.2-21.9) |
| **STRIVE;**  **NCT01664923** | | 2 | - Randomized - Parallel Assignment - Comparative | Yes | Multicenter | Enzalutamide 160 mg | | 198^a^ | NR |
|  |  |  |  |  |  | Bicalutamide 50 mg | | 198^a^ |  |
| **Alliance A031201;**  **NCT01949337** | | 3 | - Randomized - Parallel Assignment - Comparative | No | Multicenter | Enzalutamide 160 mg | | 657 | NR |
|  |  |  |  |  |  | Enzalutamide 160 mg  Abiraterone acetate 1000 mg  Prednisone 5 mg | | 654 |  |
| **NCT02125357** | | 2 | - Randomized - Crossover Assignment^b^ - Comparative | No | Multicenter | Abiraterone acetate 1000 mg once daily  Prednisone 5 mg twice daily  (crossed over to other arm at progression) | | 101 | Median follow-up: 30.7 months (IQR: 25.1-36.2) |
|  |  |  |  |  |  | Enzalutamide 160 mg  (crossed over to other arm at progression) | | 101 |  |
| **ACIS;**  **NCT02257736** | | 3 | - Randomized - Parallel Assignment - Comparative | Yes | Multicenter | Apalutamide 240 mg  Abiraterone acetate 1000 mg  Prednisone 5 mg twice daily | | 492 | Primary analysis: median follow-up of 25.7 months  Final OS analysis: median follow-up of 54.8 months |
|  |  |  |  |  |  | Placebo  Abiraterone acetate 1000 mg  Prednisone 5 mg twice daily | | 490 |  |
| **ODENZA;**  **NCT03314324** | | 2 | - Randomized - Crossover Assignment^c^ - Comparative | No | Multicenter | Darolutamide 600mg twice daily | | 249 | NR |
|  |  |  |  |  |  | Enzalutamide 160 mg once daily | |  |  |
| **NCT04862091** | | 2 | - Randomized - Parallel Assignment - Comparative | No | Multicenter | Abiraterone acetate 1000 mg (ZYTIGA® formulation) once daily  Prednisone 5 mg twice daily | | 34 | 84 days |
|  |  |  |  |  |  | Abiraterone acetate (tablets) 300mg once daily  Prednisone 5 mg twice daily | | 34 |  |
| **SPARE;**  **NCT02077634** | | 2 | - Randomized - Parallel Assignment - Comparative | No | Multicenter | Abiraterone acetate 1000 mg daily  Prednisone 5 mg twice daily | | 34 | 12 months |
|  |  |  |  |  |  | LHRH therapy  Abiraterone acetate 1000 mg daily  Prednisone 5 mg twice daily | | 34 |  |
| **CALGB 9182** | | 3 | - Randomized - Parallel Assignment - Comparative | No | NR | Mitoxantrone 14 mg/m^2^ (3-weekly dosing)  Hydrocortisone 40 mg daily | | 119 | 24-month follow-up period^d^ |
|  |  |  |  |  |  | Hydrocortisone 40 mg daily | | 123 |  |
| **Berry et al. 2002** | | 3 | - Randomized - Parallel Assignment - Comparative | No | Multicenter | Mitoxantrone 12 mg/m^2^ once every 3 weeks (for 6 cycles)  Prednisone 5 mg twice daily | | 56 | Median follow up: 21.8 months (range 2.4-50) |
|  |  |  |  |  |  | Prednisone 5 mg twice daily | | 63 |  |
| **TAX 327** | | 3 | - Randomized - Parallel Assignment - Comparative | No | Multicenter | Docetaxel 75 mg/m^2^ (3-weekly dosing)  Prednisone 5 mg twice daily | | 335 | Median follow-up: 20.8 months |
|  |  |  |  |  |  | Docetaxel 30 mg/m^2^ (weekly dosing)  Prednisone 5 mg twice daily | | 334 | Median follow-up: 20.7 months |
|  |  |  |  |  |  | Mitoxantrone 12 mg/m^2^  Prednisone 5 mg twice daily | | 337 | Median follow-up: 20.7 months |
| **NCT00436839** | | 3 | - Randomized - Parallel Assignment - Comparative | No | Multicenter | Docetaxel 75 mg/m^2^ (3-weekly dosing)  Prednisone 10 mg daily | | 113 | Median follow-up: 39.13 months |
|  |  |  |  |  |  | Mitoxantrone 12 mg/m^2^ (3-weekly dosing)  Prednisone 10 mg daily | | 115 | Median follow-up: 28.45 months |
| **PROSTY;**  **NCT00255606** | | 3 | - Randomized - Parallel Assignment - Comparative | No | Multicenter | Docetaxel 50 mg/m^2^ (2-weekly dosing)  Prednisone 10 mg | | 177 | Median duration of follow-up of patients alive after randomization: 18 months (IQR 6-20) |
|  |  |  |  |  |  | Docetaxel 75mg/m^2^ (3-weekly dosing)  Prednisone 10 mg | | 184 |  |
| **TIPC** | | 2 | - Randomized - Parallel Assignment - Comparative | NR | Multicenter | Docetaxel 30 mg/m^2^  Prednisolone 5 mg twice daily | | 71 | NR |
|  |  |  |  |  |  | Prednisolone 5 mg twice daily | | 63 |  |
| **FIRSTANA;**  **NCT01308567** | | 3 | - Randomized - Parallel Assignment - Comparative | No | Multicenter | Cabazitaxel 20 mg/m^2^  Prednisone 10 mg | | 389 | NR |
|  |  |  |  |  |  | Cabazitaxel 25 mg/m^2^  Prednisone 10 mg | | 388 |  |
|  |  |  |  |  |  | Docetaxel 75 mg/m^2^  Prednisone 10 mg | | 391 |  |
| **NCT02218606** | | 2 | - Randomized - Parallel Assignment^c^ - Non-comparative | No | Multicenter | Cabazitaxel 25 mg/m^2^  Abiraterone acetate 1000 mg  Prednisone 5 mg twice daily | | 39 | NR |
|  |  |  |  |  |  | Abiraterone acetate 1000 mg  Prednisone 5 mg twice daily | | 42 |  |
| **NCT02254785** | | 2 | - Randomized - Parallel Assignment^d^ - Comparative | No | Multicenter | Cabazitaxel 25 mg/m^2^ (as first-line treatment)  Prednisone 5 mg twice daily | | 45^e^ | Median follow-up: 21.9 months |
|  |  |  |  |  |  | AAPorENZA ([abiraterone acetate 1000 mg plus prednisone 5 mg twice daily or enzalutamide 160 mg]) (as first-line treatment) | | 50^e^ |  |
| **D9901;**  **NCT00005947** | | 3 | - Randomized - Parallel Assignment - Comparative | Yes | Multicenter | Sipuleucel-T | | 82 | 36 months |
|  |  |  |  |  |  | Placebo | | 45 |  |
| **D9902A;**  **NCT01133704** | | 3 | - Randomized - Parallel Assignment - Comparative | Yes | Multicenter | Sipuleucel-T | | 65 | All patients were to be followed for 36 months from the time of randomization or until death, whichever occurred first^c^ |
|  |  |  |  |  |  | Placebo | | 33 |  |
| **IMPACT;**  **NCT00065442** | | 3 | - Randomized - Parallel Assignment - Comparative | Yes | Multicenter | Sipuleucel-T | | 341 | 34.1 months |
|  |  |  |  |  |  | Placebo | | 171 |  |
| **ERA 223;**  **NCT02043678** | | 3 | - Randomized - Parallel Assignment - Comparative | Yes | Multicenter | Radium-223 55 kBq/kg  Abiraterone acetate 1000 mg  Prednisone or prednisolone 5 mg | | 401 | Median follow-up was 21·2 months (IQR 17·0–25·8) |
|  |  |  |  |  |  | Placebo  Abiraterone acetate 1000 mg  Prednisone or prednisolone 5 mg twice daily | | 405 |  |
| **EORTC 1333/PEACEIII;**  **NCT02194842** | | 3 | - Randomized - Parallel Assignment - Comparative | No | Multicenter | Radium-223 50 kBq/kg  Enzalutamide 160 mg | | 253 | Median follow-up in patients without BPA: 36.7 months  Median follow-up in patients receiving BPA: 23.1 months |
|  |  |  |  |  |  | Enzalutamide 160 mg | |  |  |
| **IPATential150;**  **NCT03072238** | | 3 | - Randomized - Parallel Assignment - Comparative | Yes | Multicenter | Ipatasertib 400 mg  Abiraterone acetate 1000 mg  Prednisone 5 mg | | 547 | Median follow-up: 31 months |
|  |  |  |  |  |  | Placebo  Abiraterone acetate 1000 mg  Prednisone 5 mg | | 554 |  |
| **PROpel;**  **NCT03732820** | | 3 | - Randomized - Parallel Assignment - Comparative | Yes | Multicenter | Olaparib 300 mg twice daily  Abiraterone acetate 1000mg once daily  Prednisone/Prednisolone 5mg twice daily | | 399^f^ | Median follow-up for disease progression: 19.3 months (range 0.03-30.59) |
|  |  |  |  |  |  | Placebo  Abiraterone acetate 1000mg once daily  Prednisone/Prednisolone 5mg twice daily | | 397^f^ | Median follow-up for disease progression: 19.4 months (0.03-30.16) |
| **BRCAAway;**  **NCT03012321** | | 2 | - Randomized - Crossover Assignment^b^ - Comparative | No | Multicenter | Olaparib 300 mg twice daily | | 20 | Median follow-up was 16.8 months |
|  |  |  |  |  |  | Olaparib 300 mg twice daily  Abiraterone acetate 1000mg once daily  Prednisone 5mg twice daily | | 20 | Median follow-up was 12.2 months |
|  |  |  |  |  |  | Abiraterone acetate 1000mg  Prednisone 5mg twice daily | | 20 | Median follow-up was 8.3 months |
|  | This characteristic is mutually exclusive or very different between TALAPRO-2 and comparator study. | | | | | |  |  |  |

^a^ The STRIVE trial enrolled patients with CRPC, irrespective of presence of metastatic disease. A subgroup analysis was available for CRPC patients with metastatic disease (enzalutamide [n= 128], bicalutamide [n=129]) and only outcomes pertaining to this subgroup were discussed in the report.

^b^ Patients were allowed to cross over to the alternate treatment arm after progression.

^c^ Patients crossed over to the alternate treatment arm at week 12.
^d^ Records reporting follow-up time in years were converted to months.
^e^ The trial enrolled 27.3% patients who received prior docetaxel for mCRPC. A subgroup analysis was available for patients with no prior docetaxel use
^f^ Patients in the PROpel trial consisted of 25.8% in the Olaparib + AAP arm and 20.2% in the placebo + AAP arm who were symptomatic (defined as those with a BPI-SF score ≥4 and/or opiate use).
^h^ Patients randomized reflect those within the HRR BM+ cohort as limited data for the HRR BM- cohort was reported.
Abbreviations: AAP = abiraterone acetate plus prednisone; BM = biomarker; BPI-SF = Brief Pain Inventory - Short Form; CRPC = castration-resistant prostate cancer; HRR = homologous recombination repair; IQR = interquartile range; kBq = kilobecquerel; kg = kilogram; mCRPC = metastatic castration-resistant prostate cancer; mg = milligram; NA = not applicable; NR = not reported.

Table 7: Patient Eligibility Criteria

| **Trial; NCT** | **Age/Status** | **Disease-Related** |
| --- | --- | --- |
| **TALAPRO-2;**  **NCT03395197** | - ≥18 years | - Histologically or cytologically confirmed adenocarcinoma of the prostate |
|  | - ECOG ≤1 | - Asymptomatic or mildly symptomatic mCRPC |
|  | - Life expectancy ≥12 months | - Surgically or medically castrated, with serum testosterone ≤50 ng/dL (≤1.73 nmol/L) |
|  |  | - Metastatic disease in bone documented on bone scan or in soft tissue |
|  |  | - Treatment naive in the mCRPC state. ADT is not exclusionary |
|  |  | - Progressive disease at entry: minimum of 2 rising PSA values, soft tissue disease progression, or bone disease progression |
| **COU-AA-302;**  **NCT00887198** | - ≥18 years | - Histologically or cytologically confirmed adenocarcinoma of the prostate |
|  | - ECOG 0-1 | - Asymptomatic or mildly symptomatic from prostate cancer |
|  | - Life expectancy ≥6 months | - Surgically or medically castrated, with serum testosterone <50 ng/dL (<1.73 nmol/L) |
|  |  | - mCRPC |
|  |  | - No prior treatment with cytotoxic chemotherapy, ketoconazole, or abiraterone acetate. Previous therapy with an antiandrogen is required |
|  |  | - PSA progression according to PCWG - 2 criteria or radiographic progression in soft tissue or bone with or without PSA progression |
| **NCT01591122** | - ≥18 years | - Confirmed diagnosis of adenocarcinoma of the prostate |
|  | - ECOG 0-1 | - Asymptomatic or mildly symptomatic from prostate cancer |
|  | - Life expectancy ≥6 months | - Surgically or medically castrated, with testosterone levels of <50 ng/dL |
|  |  | - Evidence of metastatic disease |
|  |  | - No prior cytotoxic chemotherapy, radiation or radionuclide therapy, or biologic therapy for mCRPC. Previous antiandrogen therapy and progression after withdrawal is required |
|  |  | - PSA progression based on PCWG2 criteria or radiographic progression as per modified RECIST |
| **NCT01867710** | - ≥18 years | - Histologically or cytologically confirmed adenocarcinoma of the prostate |
|  | - ECOG 0-1 | - Asymptomatic from prostate cancer |
|  | - Life expectancy ≥6 months | - Surgically or medically castrated, with testosterone levels of <50 ng/dL (<2.0 nmol/L) |
|  |  | - Metastatic disease documented by positive bone scan or by computed tomography or magnetic resonance imaging |
|  |  | - No prior cytotoxic chemotherapy, biologic therapy, or androgen receptor inhibition with abiraterone acetate plus prednisone for mCRPC |
|  |  | - Progression documented by PSA according to PCWG2 or radiographic progression according to modified RECIST v1.1 criteria |
| **STAAR;**  **NCT02737332** | - ≥18 years | - Pathologically confirmed adenocarcinoma of the prostate |
|  | - ECOG 0-1 | - No symptomatic criterion reported |
|  | - Life expectancy ≥6 months | - mCRPC with serum testosterone ≤50 ng/dL |
|  |  | - Metastatic disease documented by CT/ MRI or bone scan |
|  |  | - No prior abiraterone acetate, enzalutamide, radium-223, or chemotherapy. Ongoing therapy with a GnRH agonist or antagonist |
|  |  | - Disease progression according to PCWG2 criteria: 2 rises of PSA from a baseline measurement of at least 2 ng/mL, imaging progression by RECIST criteria, or nuclear scan progression by new lesion |
| **Hu et al. 2020** | - ≥18 years | - Histologically or cytologically confirmed adenocarcinoma of the prostate |
|  | - No ECOG criterion reported | - No symptomatic criterion reported |
|  | - No life expectancy criterion reported | - Patients have mCRPC, no serum testosterone criteria reported |
|  |  | - Patients have mCRPC |
|  |  | - No prior chemotherapy. Previous ADT is required |
|  |  | - No progressive disease criterion reported |
| **PREVAIL;**  **NCT01212991** | - ≥18 years | - Histologically or cytologically confirmed adenocarcinoma of the prostate |
|  | - ECOG 0-1 | - Asymptomatic or mildly symptomatic from prostate cancer |
|  | - Life expectancy ≥6 months | - Surgically or medically castrated, with serum testosterone ≤50 ng/dL (≤1.73 nmol/L) |
|  |  | - mCRPC |
|  |  | - No prior treatment with cytotoxic chemotherapy, ketoconazole, or abiraterone acetate. Continued ADT is required. |
|  |  | - Progressive disease defined by rising PSA levels or progressive soft tissue or bony disease |
| **NCT04862091** | - ≥18 years | - Histologically or cytologically confirmed adenocarcinoma of the prostate |
|  | - ECOG ≤1 | - Asymptomatic or mildly symptomatic mCRPC |
|  | - Life expectancy ≥6 months | - Surgically or medically castrated, with serum testosterone <50 ng/dL (<1.7 nmol/L) |
|  |  | - Metastatic lesions with imaging evidence (such as positive bone scan or metastatic lesions on CT/MRI) |
|  |  | - No prior biotherapy or cytotoxic chemotherapy, CYP17 inhibitors, or investigational drugs or marketed drugs of new androgen receptor antagonists |
|  |  | - Progressive disease at entry: minimum of 3 rising PSA values, soft tissue disease progression, or bone disease progression |
| **NCT02294461** | - ≥18 years | - Histologically confirmed adenocarcinoma of the prostate |
|  | - ECOG 0-1 | - Asymptomatic or mildly symptomatic from prostate cancer |
|  | - No life expectancy criterion reported | - Patients have mCRPC, no serum testosterone criteria reported |
|  |  | - Patients have mCRPC |
|  |  | - No prior treatment with cytotoxic chemotherapy. Ongoing ADT is required |
|  |  | - Progressive disease as defined by rising PSA levels or progressive soft tissue or bone disease |
| **SPARE;**  **NCT02077634** | - ≥18 years | - Histologically or cytologically confirmed adenocarcinoma of the prostate |
|  | - ECOG ≤2 | - Asymptomatic or mildly symptomatic from prostate cancer |
|  | - Life expectancy ≥6 months | - Medically castrated, with testosterone levels of <20-50 ng/dl (<2.0 nM) |
|  |  | - Metastatic disease documented by positive CT/MRI and/or bone scan. If lymph node is the only evidence of metastasis, it must be ≥2 cm in diameter |
|  |  | - No prior cytotoxic chemotherapy, biologic therapy, abiraterone acetate or other CYP17 inhibitors, enzalutamide or investigational agents |
|  |  | - Prostate cancer progression documented by PSA according to PCWG2 or radiographic progression according to modified RECIST criteria |
| **TERRAIN;**  **NCT01288911** | - ≥18 years | - Histologically confirmed adenocarcinoma of the prostate |
|  | - ECOG 0-1 | - Asymptomatic or mildly symptomatic from prostate cancer |
|  | - Life expectancy ≥12 months | - Surgically or medically castrated, with serum testosterone ≤50 ng/dL (≤1.7 nmol/L) |
|  |  | - Metastatic disease in bone documented on bone scan or in soft tissue |
|  |  | - No prior cytotoxic chemotherapy or ketoconazole for prostate cancer |
|  |  | - Progressive disease at entry: minimum of 3 rising PSA values, soft tissue disease progression, or bone disease progression |
| **STRIVE;**  **NCT01664923^a^** | - ≥18 years | - Histologically or cytologically confirmed adenocarcinoma of the prostate |
|  | - ECOG 0-1 | - Asymptomatic or mildly symptomatic from prostate cancer |
|  | - Life expectancy ≥12 months | - Surgically or medically castrated, with serum testosterone ≤50 ng/dL (≤1.73 nmol/L) |
|  |  | - mCRPC |
|  |  | - No prior radiation or radionuclide therapy, ketoconazole, abiraterone, or cytotoxic chemotherapy for prostate cancer |
|  |  | - Progressive disease at entry: minimum of 2 rising PSA values, soft tissue disease progression, or bone disease progression |
| **Alliance A031201;**  **NCT01949337** | - ≥18 years | - Histologically or cytologically confirmed adenocarcinoma of the prostate |
|  | - ECOG 0-1 | - Asymptomatic or mildly symptomatic from prostate cancer |
|  | - No life expectancy criterion reported | - Total testosterone ≤ 50 ng/dL (1.7 nmol/L) |
|  |  | - mCRPC |
|  |  | - No prior taxane-based chemotherapy, enzalutamide, abiraterone, other novel antiandrogen, or androgen synthesis inhibitor. Ongoing ADT is required |
|  |  | - Progressive disease at entry: minimum of 2 rising PSA values, soft tissue disease progression, or bone disease progression |
| **NCT02125357** | - ≥18 years | - Histologically confirmed adenocarcinoma of the prostate |
|  | - ECOG 0-2 | - No symptomatic criterion reported |
|  | - Life expectancy ≥6 months | - Surgically or medically castrated, with serum testosterone ≤1.7 nmol/L |
|  |  | - Evidence of metastatic disease on bone scan or CT scan |
|  |  | - No prior therapy with CYP17 inhibitors, enzalutamide or other experimental anti-androgens, or systemic chemotherapy for mCRPC |
|  |  | - Progressive disease at entry: minimum of 2 rising PSA values, soft tissue disease progression, or bone disease progression |
| **ACIS;**  **NCT02257736** | - ≥18 years | - Histologically or cytologically confirmed adenocarcinoma of the prostate |
|  | - ECOG 0-1 | - A score of ≤3 on the BPI-SF Question #3 (worst pain in last 24 hours) |
|  | - No life expectancy criterion reported | - Surgically or medically castrated, with testosterone levels of <50 ng/dL (<1.7 nM) |
|  |  | - Metastatic disease as documented by bone scan or metastatic lesions by CT / MRI scans |
|  |  | - No prior chemotherapy for prostate cancer (unless administered in an adjuvant or neoadjuvant setting) or androgen biosynthesis inhibitors |
|  |  | - Progression documented by PSA according to PCWG2 or radiographic progression of soft tissue according to modified RECIST v1.1 modified based on PCWG2, or radiographic progression of bone according to PCWG2 |
| **ODENZA;**  **NCT03314324** | - ≥18 years | - Histologically confirmed adenocarcinoma of the prostate |
|  | - ECOG 0-1^b^ | - Asymptomatic or mildly symptomatic prostate cancer |
|  | - No life expectancy criterion reported | - Surgically or medically castrated, with serum testosterone <50 ng/dL (<1.7 nmol/L) |
|  |  | - Evidence of metastatic disease |
|  |  | - Prior abiraterone, enzalutamide, ODM-201, ARN- 509 or any other next-generation AR axis-targeting drug, taxanes, or radium-223 |
|  |  | - Progressive disease (PSA progression or radiological progression or clinical progression) as per PCWG3 criteria |
| **CALGB 9182** | - No age criterion reported | - No prostate cancer confirmation criterion reported |
|  | - No ECOG criterion reported | - No symptomatic criterion reported |
|  | - No life expectancy criterion reported | - Surgically or medically castrated, no serum testosterone criterion reported |
|  |  | - Metastatic prostate cancer |
|  |  | - No prior treatment criterion reported |
|  |  | - Antiandrogen withdrawal and subsequent documented disease progression is required |
| **Berry et al. 2002** | - No age criterion reported | - Adenocarcinoma of the prostate diagnosis |
|  | - ECOG 0-2 | - Asymptomatic carcinoma of the prostate |
|  | - No life expectancy criterion reported | - Surgically or medically castrated, no serum testosterone criterion reported |
|  |  | - No metastatic criterion reported |
|  |  | - No prior immunotherapy, chemotherapy, or concurrent use of exogenous corticosteroids |
|  |  | - Pretreatment disease progression was defined as increasing PSA (2-fold or greater increase over 2 determinations), 25% increase in number of bone scan lesions or 25% increase in size of soft tissue lesions |
| **TAX 327** | - No age criterion reported | - No prostate cancer confirmation criterion reported |
|  | - Karnofsky performance status score of ≥60% | - No symptomatic criterion reported |
|  | - No life expectancy criterion reported | - No serum testosterone criteria reported |
|  |  | - mHRPC |
|  |  | - No prior cytotoxic agents (except estramustine) or radioisotopes |
|  |  | - Clinical or radiologic evidence of progressive disease or three increasing values of serum PSA |
| **NCT00436839** | - No age criterion reported | - Histologically or cytologically proven prostate adenocarcinoma |
|  | - Karnofsky performance status score of ≥70% | - No symptomatic criterion reported |
|  | - Life expectancy ≥3 months | - Surgically or medically castrated, with serum testosterone <50 ng/dL (ie <1.735 nmol/L) |
|  |  | - Hormone-refractory (androgen-independent) metastatic prostate cancer |
|  |  | - No prior treatment with cytotoxic agent (except estramustine), isotope therapy, bisphosphonates, or any other anti-cancer therapy |
|  |  | - Documented progression detected by PSA increase |
| **PROSTY;**  **NCT00255606** | - ≥18 years | - Histologically or cytologically confirmed adenocarcinoma of the prostate |
|  | - WHO performance status 0-2 | - No symptomatic criterion reported |
|  | - No life expectancy criterion specified | - Testosterone within castration levels by orchiectomy or medical castration |
|  |  | - Metastatic disease by imaging or clinical examination |
|  |  | - No prior cancer chemotherapy except estramustine, radiotherapy to >25% of bone marrow, radioisotope therapy, or cytostatic treatment |
|  |  | - Hormone-refractory disease, defined as PSA level > 10 µg/L and rising between 2 sequential measurements |
| **TIPC** | - <85 years | - No prostate cancer confirmation criterion reported |
|  | - ECOG ≤2 | - No symptomatic criterion reported |
|  | - No life expectancy criterion reported | - AIPC, serum testosterone within the castration range |
|  |  | - Distant metastases |
|  |  | - No prior systemic therapy after the diagnosis of AIPC |
|  |  | - Disease progression: Increase in PSA levels in two blood samples |
| **FIRSTANA;**  **NCT01308567** | - ≥18 years | - Histologically- or cytologically-confirmed prostate adenocarcinoma |
|  | - ECOG ≤2 | - No symptomatic criterion reported |
|  | - No life expectancy criterion reported | - Effective castration (serum testosterone levels ≤0.50 ng/mL) by orchiectomy and/or medically |
|  |  | - Metastatic disease |
|  |  | - No prior chemotherapy for prostate cancer, isotope therapy, whole pelvic radiotherapy, or radiotherapy to >30% of bone marrow |
|  |  | - Progressive disease defined as one of the following at entry: increase in measurable disease, appearance of new lesions, or rising PSA |
| **NCT02218606** | - ≥18 years | - Histologic or cytologic diagnosis of prostate cancer |
|  | - ECOG 0-2 | - No symptomatic criterion reported |
|  | - No life expectancy criterion reported | - Surgically or medically castrated, with serum testosterone <50 ng/dL |
|  |  | - mCRPC |
|  |  | - No prior abiraterone acetate or other specific CYP17 inhibitors, or chemotherapy regimen |
|  |  | - Progressive disease defined as one of the following: PSA progression, soft-tissue progression, or progression of bone disease |
| **NCT02254785^c^** | - ≥18 years | - Histological diagnosis of prostate adenocarcinoma |
|  | - ECOG 0-2 | - No symptomatic criterion reported |
|  | - No life expectancy criterion reported | - Castrate levels of testosterone (serum testosterone < 50 ng/dL (1.7 nmol/L) |
|  |  | - Evidence of metastatic disease on a chest, abdominal, or pelvic CT scan and/or bone scan |
|  |  | - No prior chemotherapy (one course of docetaxel was allowed), next-generation antiandrogen or CYP17 inhibitors, and contraindications to cabazitaxel, abiraterone, or enzalutamide |
|  |  | - Evidence of radiological and/or PSA progression. For PSA progression, there must be ≥2 sequential rising PSA values (PCWG2 criteria) |
| **D9901;**  **NCT00005947** | - ≥18 years | - Histologically confirmed adenocarcinoma of the prostate |
|  | - ECOG 0-1 | - Subjects have no cancer-related pain and do not regularly require analgesics for cancer-related pain |
|  | - Life expectancy ≥3 months | - Hormonal therapy consisting of castration by orchiectomy or medically with castration levels of testosterone (< 50 ng/dL) |
|  |  | - Metastatic disease as evidenced by soft tissue and/or bony metastases |
|  |  | - No prior immunotherapy or concurrent systemic corticosteroids |
|  |  | - Progressive disease, as defined by PSA Consensus Criteria |
| **D9902A;**  **NCT01133704** | - ≥18 years | - Histologically documented adenocarcinoma of the prostate |
|  | - ECOG 0-1 | - Patients who required opioid analgesics for cancer pain were excluded |
|  | - Life expectancy ≥3 months | - Castration levels of testosterone (defined as <50 ng/dL) |
|  |  | - Metastatic disease as evidenced by soft tissue and/or bony metastases |
|  |  | - No prior immunotherapy |
|  |  | - Evidence of progressive disease based on successive radiographic studies or PSA progression, as defined by PSA Consensus Criteria |
| **IMPACT;**  **NCT00065442** | - ≥18 years | - Histologically documented adenocarcinoma of the prostate |
|  | - ECOG 0-1 | - Asymptomatic and minimally symptomatic disease; the absence of or minimal current cancer-related pain |
|  | - Life expectancy ≥6 months | - Medical or surgical castration with serum testosterone level of <50 ng/dL (≤1.73 nmol/L) |
|  |  | - Cancer that has spread outside the prostate (metastatic) to lymph nodes or bone |
|  |  | - No prior systemic glucocorticoids, external-beam radiation, surgery, or systemic therapy for prostate cancer (except medical or surgical castration) |
|  |  | - Disease progression as demonstrated by PSA progression OR progression of measurable disease OR progression of non-measurable disease |
| **ERA 223;**  **NCT02043678** | - ≥ 18 years | - Histologically confirmed adenocarcinoma of the prostate |
|  | - ECOG 0-1 | - Asymptomatic or mildly symptomatic prostate cancer |
|  | - Life expectancy ≥6 months | - Maintenance of medical castration or surgical castration with testosterone <50 ng/dL (1.7nmol/L) |
|  |  | - ≥bone metastases on bone scan ≥2 weeks prior to randomization with no lung, liver, other visceral and/or brain metastasis |
|  |  | - No prior cytotoxic chemotherapy for the treatment of CRPC, including taxanes, mitoxantrone and estramustine |
|  |  | - Prostate cancer progression documented by PSA according to PCWG2 criteria or radiological progression according to RECIST version 1.1 |
| **EORTC 1333/ PEACEIII;**  **NCT02194842** | - ≥ 18 years | - Histologically confirmed diagnosis of prostate adenocarcinoma |
|  | - WHO performance status 0-1 | - Asymptomatic or mildly symptomatic (defined as no opioids and Brief Pain Inventory score) |
|  | - No life expectancy criterion reported | - Castrate serum levels of testosterone (< 50 ng/dL) |
|  |  | - Metastatic to bone with ≥ 2 bone metastases with or without additional lymph node metastases. Visceral metastases are not allowed |
|  |  | - No prior docetaxel, enzalutamide, radium-223, CYP17 inhibitors, ketoconazole, radiotherapy, radionuclides, experimental drugs, or anticancer therapy |
|  |  | - Progressive castration-resistant prostate cancer according to PCWG2 |
| **IPATential150;**  **NCT03072238** | - ≥ 18 years | - Histologically confirmed prostate adenocarcinoma |
|  | - ECOG 0-1 | - Asymptomatic or mildly symptomatic form of prostate cancer |
|  | - Life expectancy ≥6 months | - Ongoing medical androgen deprivation or bilateral orchiectomy, with serum testosterone ≤50 ng/dL (≤1.7 nmol/L) |
|  |  | - Metastatic disease defined by clear evidence of bone lesions on bone scan and/or measurable soft tissue disease |
|  |  | - No prior chemotherapy, biological therapy, CYP17 inhibitors, androgen blockers, radiopharmaceuticals, or PI3K inhibitors |
|  |  | - Progressive disease before initiating study treatment |
| **PROpel;**  **NCT03732820^d^** | - ≥18 years (≥19 years in South Korea) | - Histologically or cytologically confirmed prostate adenocarcinoma |
|  | - ECOG 0-1 | - No symptomatic criterion reported |
|  | - Life expectancy ≥6 months | - Ongoing androgen deprivation with gonadotropin-releasing hormone analogue or bilateral orchiectomy, with serum testosterone <50ng/dL (2.0 nmol/L) |
|  |  | - Metastatic status defined as at least 1 documented metastatic lesion |
|  |  | - First-line mCRPC |
|  |  | - Documented evidence of progressive disease |
| **BRCAAway;**  **NCT03012321** | - ≥18 years | - Histological or cytological proof of prostate adenocarcinoma |
|  | - ECOG 0-2 | - No symptomatic criterion reported |
|  | - Life expectancy ≥6 months | - mCRPC, no serum testosterone criterion reported |
|  |  | - No prior CYP17, PARP inhibitors, chemotherapy, enzalutamide, ARN-509 or other investigational androgen receptor-directed therapy |
|  |  | - Documented progressive mCRPC: PSA progression, Progression of bidimensionally measurable soft tissue or nodal metastasis, or Progression of bone disease (evaluable disease) (new bone lesion(s)) |

|  | This characteristic is mutually exclusive or very different between TALAPRO-2 and comparator study. |
| --- | --- |

^a^ Only outcomes pertaining to CRPC patients with metastatic disease were presented.
^b^ ECOG 2 was accepted if the impairment was not due to prostate cancer
^c^ Only outcomes pertaining to the docetaxel-naïve subgroup were presented.
^d^ Patients in the PROpel trial consisted of 25.8% in the Olaparib + AAP arm and 20.2% in the placebo + AAP arm who were symptomatic (defined as those with a BPI-SF score ≥4 and/or opiate use)

Abbreviations: ADT = androgen deprivation therapy; anti-PD-1 = anti-programmed cell death; anti-PD-L = anti-programmed cell death-ligand; AAP = abiraterone acetate plus prednisone; BPI-SF = Brief Pain Inventory-Short Form; CRPC = castration-resistant prostate cancer; CT = computed tomography; dL = decaliter; EAU = European Association of Urology; ECOG = Eastern Cooperation Oncology Group; GnRH = gonadotropin releasing hormone; IDC-P = intraductal carcinoma of the prostate; L = liter; mCRPC = metastatic castration-resistant prostate cancer; mHRPC = metastatic hormone-refractory prostate cancer; MRI = magnetic resonance imaging; ng = nanogram; nmol = nanomole; PCWG2 = Prostate Cancer Working Group 2; PCWG3 = Prostate Cancer Working Group 3; PARP = poly (adenosine diphosphate [ADP]-ribose) polymerase; PSA = prostate-specific antigen; RECIST = Response Evaluation Criteria in Solid Tumors; WHO = World Health Organization.

Table 8: Reported Efficacy Outcomes

| **Trial; NCT** | **OS** | **rPFS** | **PFS** | **PFS2** | **ORR** | **CR** | **PR** | **DoR** | **PSA response** | **Time to PSA progression** | **Time to cytotoxic chemotherapy** | **Time to initiation of antineoplastic therapy** | **Time to first SSE** | **Time to opioid use** |
| --- | --- | --- | --- | --- | --- | --- | --- | --- | --- | --- | --- | --- | --- | --- |
| **TALAPRO-2;**  **NCT03395197** | ✓ | ✓ | NR | ✓ | ✓ | ✓ | ✓ | ✓ | ✓ | ✓ | ✓ | ✓ | ✓ | ✓ |
| **COU-AA-302;**  **NCT00887198** | ✓ | ✓ | NR | NR | ✓ | ✓ | NR | NR | ✓ | ✓ | ✓ | NR | NR | ✓ |
| **NCT01591122** | NR | NR | NR | NR | ✓ | ✓ | ✓ | NR | ✓ | ✓ | ✓ | NR | NR | ✓ |
| **NCT01867710** | NR | ✓ | NR | NR | ✓ | NR | NR | NR | ✓ | ✓ | NR | NR | NR | NR |
| **STAAR;**  **NCT02737332** | NR | NR | NR | NR | NR | NR | NR | NR | ✓ | NR | NR | NR | NR | NR |
| **Hu et al. 2020** | ✓ | NR | NR | NR | NR | NR | NR | NR | ✓ | ✓ | NR | NR | NR | NR |
| **PREVAIL;**  **NCT01212991** | ✓ | ✓ | NR | NR | ✓ | ✓ | ✓ | NR | ✓ | ✓ | ✓ | NR | ✓ | NR |
| **NCT02294461** | ✓ | ✓ | NR | NR | ✓^a^ | ✓ | ✓ | NR | ✓ | ✓ | ✓ | NR | ✓ | NR |
| **TERRAIN;**  **NCT01288911** | NR | ✓ | ✓ | NR | ✓ | NR | NR | NR | ✓ | ✓ | NR | NR | NR | NR |
| **STRIVE; NCT01664923^b^** | NR | ✓ | ✓ | NR | ✓^a^ | ✓ | ✓ | NR | ✓ | ✓ | NR | NR | NR | NR |
| **Alliance A031201;**  **NCT01949337** | ✓ | NR | NR | NR | NR | NR | NR | NR | ✓ | NR | NR | NR | NR | NR |
| **NCT02125357^c^** | NR | NR | NR | NR | NR | NR | NR | NR | ✓ | ✓ | NR | NR | NR | NR |
| **ACIS;**  **NCT02257736** | ✓ | ✓ | NR | ✓ | ✓ | NR | NR | NR | ✓ | ✓ | ✓ | NR | NR | ✓ |
| **ODENZA;**  **NCT03314324^c^** | NR | NR | NR | NR | NR | NR | NR | NR | ✓ | NR | NR | NR | NR | NR |
| **NCT04862091** | NR | NR | NR | NR | NR | NR | NR | NR | ✓ | NR | NR | NR | NR | NR |
| **SPARE;**  **NCT02077634** | NR | ✓ | NR | NR | ✓^d^ | ✓ | ✓ | NR | ✓ | ✓ | NR | NR | NR | NR |
| **CALGB 9182** | ✓ | NR | NR | NR | ✓^d^ | ✓ | ✓ | NR | ✓ | NR | NR | NR | NR | NR |
| **Berry et al. 2002** | ✓ | NR | ✓ | NR | ✓^d^ | ✓ | ✓ | NR | ✓ | ✓ | NR | NR | NR | NR |
| **TAX 327** | ✓ | NR |  | NR | ✓ | NR | NR | NR | ✓ | NR | NR | NR | NR | NR |
| **NCT00436839** | ✓ | NR | ✓ | NR | ✓ | ✓ | ✓ | NR | ✓ | NR | NR | NR | NR | NR |
| **PROSTY;**  **NCT00255606** | ✓ | NR |  | NR | ✓ | NR | NR | NR | ✓ | NR | NR | NR | NR | NR |
| **TIPC** | ✓ | NR | ✓ | NR | NR | NR | NR | NR | ✓ | NR | NR | NR | NR | NR |
| **FIRSTANA;**  **NCT01308567** | ✓ | NR | ✓ | NR | ✓ | ✓ | ✓ | NR | ✓ | ✓^e^ | NR | NR | ✓ | NR |
| **NCT02218606^c^** | ✓ | ✓ | NR | NR | NR | NR | NR | NR | ✓ | ✓ | NR | NR | NR | NR |
| **NCT02254785^c,f^** | ✓ | NR | NR | ✓ | NR | NR | NR | NR | NR | NR | NR | NR | NR | NR |
| **D9901;**  **NCT00005947** | ✓ | NR | NR | NR | NR | NR | NR | NR | NR | NR | NR | NR | NR | NR |
| **D9902A;**  **NCT01133704** | ✓ | NR | NR | NR | NR | NR | NR | NR | NR | NR | NR | NR | NR | NR |
| **IMPACT;**  **NCT00065442** | ✓ | NR | NR | NR | NR | NR | NR | NR | ✓ | NR | ✓ | NR | NR | NR |
| **ERA 223;**  **NCT02043678** | ✓ | ✓ | NR | NR | NR | NR | NR | NR | ✓ | ✓ | ✓ | NR | NR | ✓ |
| **EORTC 1333/PEACEIII;**  **NCT02194842** | NR | NR | NR | NR | NR | NR | NR | NR | NR | NR | NR | NR | NR | NR |
| **IPATential150;**  **NCT03072238** | ✓ | ✓ | NR | NR | ✓ | ✓ | ✓ | ✓ | ✓ | ✓ | NR | NR | NR | NR |
| **PROpel;**  **NCT03732820^g^** | ✓ | ✓ | NR | ✓ | ✓ | ✓ | ✓ | NR | NR | NR | NR | NR | NR | NR |
| **BRCAAway;**  **NCT03012321** | NR | NR | ✓ | NR | NR | NR | NR | NR | ✓ | NR | NR | NR | NR | NR |

^a^ Best Overall Soft Tissue Response was reported.
^b^ Only outcomes pertaining to CRPC patients with metastatic disease were presented.
^c^ Only outcomes measured before crossover occurred were discussed in the report.
^d^ Values were calculated from CR and PR rates, where CR + PR = ORR.

^e^ PSA-PFS was reported; defined as time from randomization to the first occurrence of PSA progression or death, whichever was earlier

^f^ Only outcomes pertaining to the docetaxel-naïve subgroup were presented.
^g^ Patients in the PROpel trial consisted of 25.8% in the Olaparib + AAP arm and 20.2% in the placebo + AAP arm who were symptomatic (defined as those with a BPI-SF score ≥4 and/or opiate use)

Abbreviations: AAP = abiraterone acetate plus prednisone; BPI-SF = Brief Pain Inventory – Short Form; CR = complete response; DoR = duration of response; mCRPC = metastatic castration-resistant prostate cancer; NR = not reported; ORR = objective response rate; PFS = progression-free survival; PFS2 = progression-free survival on next line; PR = partial response; PSA = prostate-specific antigen; rPFS = radiographic progression-free survival; SSE = symptomatic skeletal event.

1. Additional Random-Effects Results

Table 9: Random-Effects P-best and SUCRA Scores for rPFS

| **Treatment** | **Probability Best (%)** | **SUCRA (%)** |
| --- | --- | --- |
| TALA+ENZA | 52 | 93 |
| CABA25+AAP | 17 | 81 |
| AA+DEX | 14 | 76 |
| AAPnoLHRH | 14 | 64 |
| OLAP+AAP | 1 | 65 |
| APA+AAP | 1 | 63 |
| ENZA | 0 | 72 |
| IPA+AAP | 0 | 49 |
| AAP5QD | 0 | 38 |
| AAP | 0 | 35 |
| Ra55+AAP | 0 | 26 |
| AAP2.5BID | 0 | 19 |
| BIC | 0 | 16 |
| BSC | 0 | 4 |

Abbreviations: rPFS = radiographic progression-free survival; SUCRA = surface under the cumulative ranking curve.

Note: for full list of treatment regimens refer to **Appendix B**.

Table 10: Random-Effects P-best and SUCRA Scores for OS

| **Treatment** | **Probability Best (%)** | **SUCRA (%)** |
| --- | --- | --- |
| DOC50+PL10 | 63 | 93 |
| TALA+ENZA | 15 | 77 |
| ENZA+AAP | 5 | 67 |
| OLAP+AAP | 4 | 62 |
| AAPorENZA | 4 | 26 |
| CABA25+PS | 3 | 71 |
| CABA20 | 2 | 67 |
| IPA+AAP | 1 | 49 |
| APA+AAP | 1 | 42 |
| DOC75+PS | 0 | 71 |
| ENZA | 0 | 59 |
| DOC30+PS | 0 | 55 |
| SIP-T | 0 | 48 |
| AAP | 0 | 34 |
| MIT12+PS | 0 | 32 |
| Ra55+AAP | 0 | 18 |
| MIT14+HC | 0 | 16 |
| BSC | 0 | 12 |

Abbreviations: OS = overall survival; SUCRA = surface under the cumulative ranking curve.

Note: for full list of treatment regimens refer to  **Appendix B**.

Figure 20: Random-Effects League Table for Time to PSA Progression


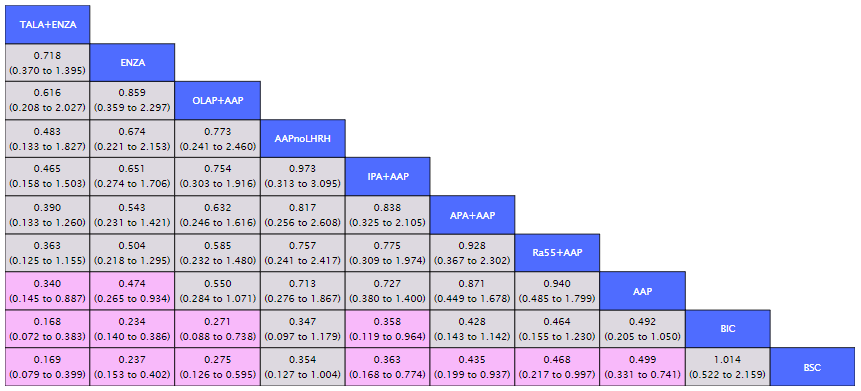


Note: Values are HRs (95% credible interval) for relative effectiveness for all possible pairs of treatments in the network.

Note: HR < 1 implies that column is better than row.

Note: Pink squares are statistically significant.

Abbreviations: HR = hazard ratio; PSA = prostate specific antigen.

Note: for full list of treatment regimens refer to  **Appendix B**.

Table 11: Random-Effects P-best and SUCRA Scores for Time to PSA Progression

| **Treatment** | **Probability Best (%)** | **SUCRA (%)** |
| --- | --- | --- |
| TALA+ENZA | 70 | 93 |
| OLAP+AAP | 12 | 74 |
| AAPnoLHRH | 8 | 58 |
| ENZA | 6 | 80 |
| IPA+AAP | 3 | 58 |
| APA+AAP | 1 | 46 |
| Ra55+AAP | 1 | 41 |
| AAP | 0 | 35 |
| BIC | 0 | 8 |
| BSC | 0 | 7 |

Abbreviations: PSA = prostate specific antigen; SUCRA = surface under the cumulative ranking curve

Note: for full list of treatment regimens refer to  **Appendix B**.

Figure 21: Random-Effects League Table for Time to Cytotoxic Chemotherapy Initiation


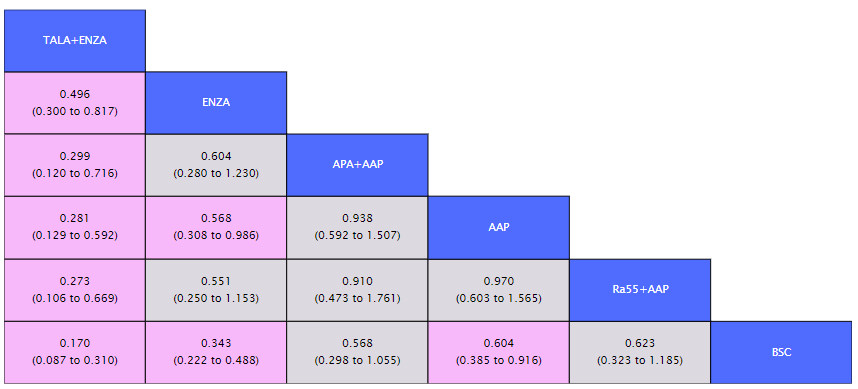


Note: Values are HRs (95% credible interval) for relative effectiveness for all possible pairs of treatments in the network

Note: HR < 1 implies that column is better than row

Note: Pink squares are statistically significant

Abbreviations: HR = hazard ratio

Note: for full list of treatment regimens refer to  **Appendix B**.

Table 12: Random-Effects P-best and SUCRA Scores for Time to Cytotoxic Chemotherapy Initiation

| **Treatment** | **Probability Best (%)** | **SUCRA (%)** |
| --- | --- | --- |
| TALA+ENZA | 98 | 99 |
| ENZA | 1 | 78 |
| APA+AAP | 1 | 47 |
| Ra55+AAP | 1 | 36 |
| AAP | 0 | 39 |
| BSC | 0 | 2 |

Abbreviations: SUCRA = surface under the cumulative ranking curve.

Note: for full list of treatment regimens refer to  **Appendix B**.

Figure 22: Random-Effects League Table for PSA Response


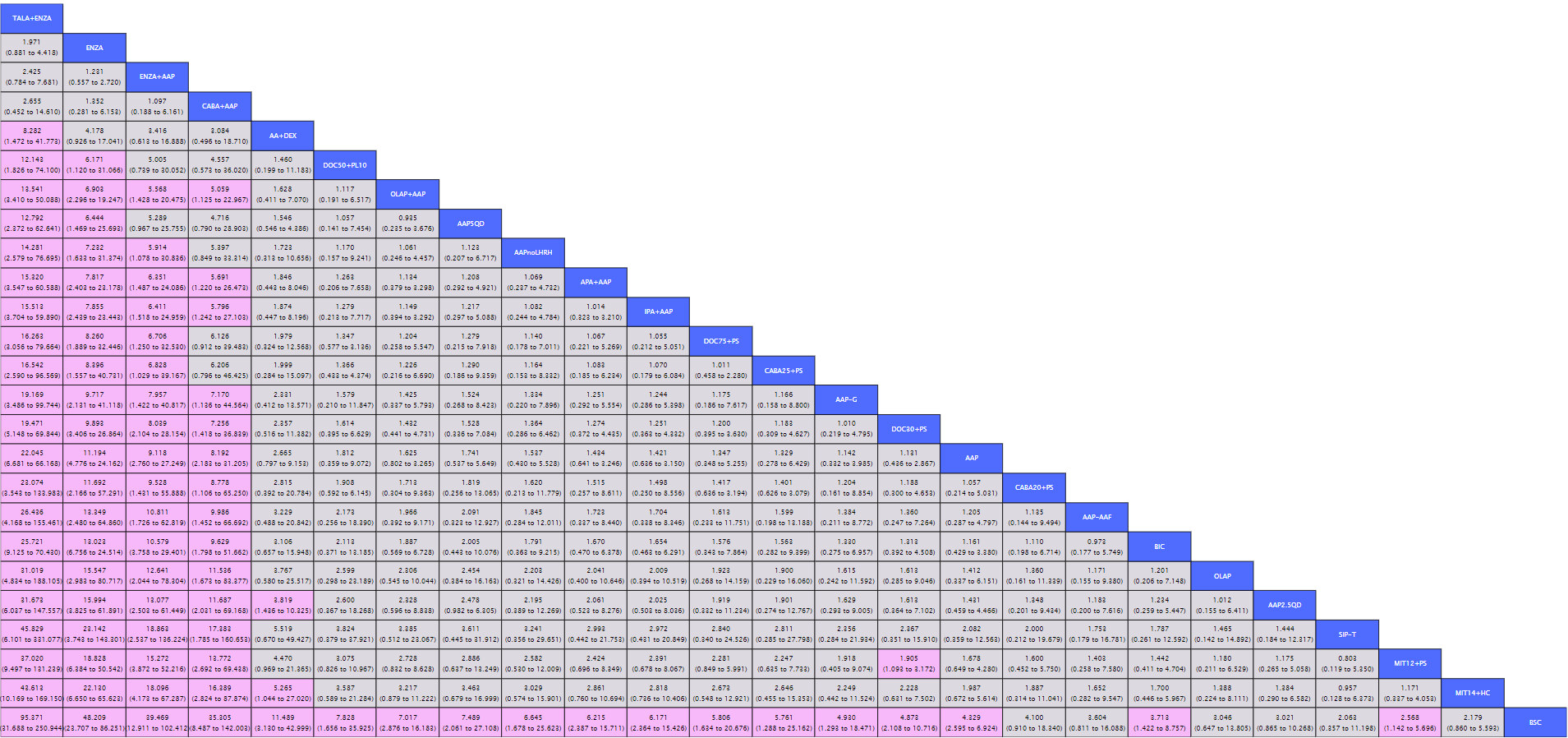


Note: Values are ORs (95% credible interval) for relative effectiveness for all possible pairs of treatments in the network.

Note: OR > 1 implies that column is better than row.

Note: Pink squares are statistically significant.

Abbreviations: OR = odds ratio.

Note: for full list of treatment regimens refer to  **Appendix B**.

Table 13: Random-Effects P-best and SUCRA Scores for PSA Response

| **Treatment** | **Probability Best (%)** | **SUCRA (%)** |
| --- | --- | --- |
| TALA+ENZA | 83 | 99 |
| CABA+AAP | 12 | 89 |
| ENZA+AAP | 3 | 90 |
| ENZA | 2 | 93 |
| AA+DEX | 0 | 73 |
| DOC50+PL10 | 0 | 63 |
| OLAP+AAP | 0 | 61 |
| AAP5QD | 0 | 60 |
| AAPnoLHRH | 0 | 56 |
| APA+AAP | 0 | 55 |
| IPA+AAP | 0 | 55 |
| DOC75+PS | 0 | 52 |
| CABA25+PS | 0 | 51 |
| AAP-G | 0 | 45 |
| DOC30+PS | 0 | 45 |
| AAP | 0 | 39 |
| CABA20+PS | 0 | 37 |
| AAP-AAF | 0 | 35 |
| BIC | 0 | 34 |
| OLAP | 0 | 30 |
| AAP2.5QD | 0 | 27 |
| SIP-T | 0 | 21 |
| MIT12+PS | 0 | 20 |
| MIT14+HC | 0 | 18 |
| BSC | 0 | 2 |

Abbreviations: PSA = prostate specific antigen; SUCRA = surface under the cumulative ranking curve.

Note: for full list of treatment regimens refer to  **Appendix B**.

Figure 23: Random-Effects League Table for ORR


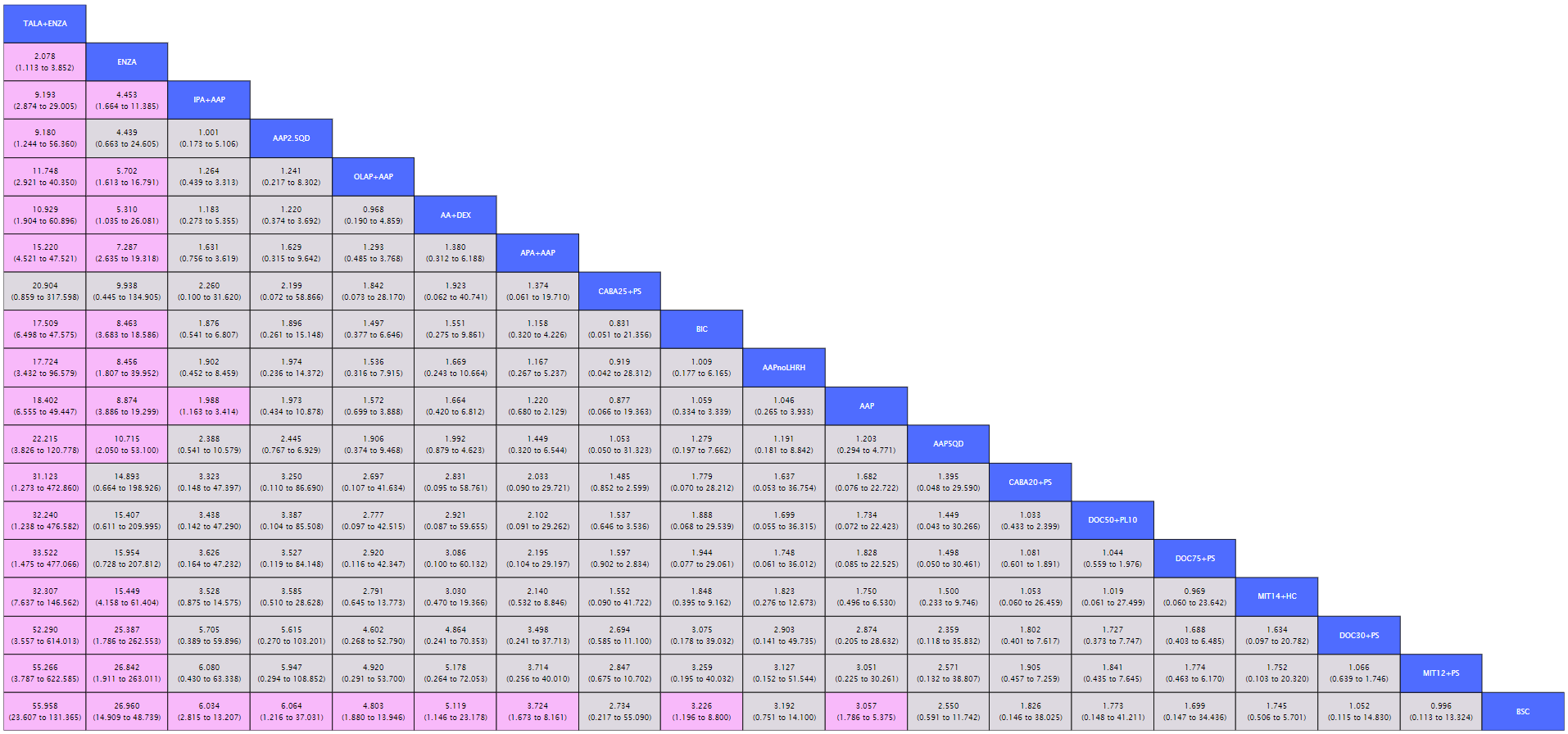


Note: Values are ORs (95% credible interval) for relative effectiveness for all possible pairs of treatments in the network.

Note: OR > 1 implies that column is better than row.

Note: Pink squares are statistically significant.

Abbreviations: OR = odds ratio; ORR = objective response rate.

Note: for full list of treatment regimens refer to  **Appendix B**.

Table 14: Random-Effects P-best and SUCRA Scores for ORR

| **Treatment** | **Probability Best (%)** | **SUCRA (%)** |
| --- | --- | --- |
| TALA+ENZA | 94 | 99 |
| CABA25+PS | 3 | 54 |
| ENZA | 1 | 93 |
| AAP2.5QD | 1 | 69 |
| IPA+AAP | 0 | 72 |
| OLAP+AAP | 0 | 64 |
| AA+DEX | 0 | 64 |
| APA+AAP | 0 | 54 |
| BIC | 0 | 49 |
| AAPnoLHRH | 0 | 48 |
| AAP | 0 | 45 |
| AAP5QD | 0 | 40 |
| CABA20+PS | 0 | 39 |
| DOC50+PL10 | 0 | 38 |
| DOC75+PS | 0 | 36 |
| MIT14+HC | 0 | 30 |
| DOC30+PS | 0 | 22 |
| MIT12+PS | 0 | 19 |
| BSC | 0 | 15 |

Abbreviations: ORR = objective response rate; SUCRA = surface under the cumulative ranking curve.

Note: for full list of treatment regimens refer to  **Appendix B**.

1. Fixed-Effect Results

Figure 24: Fixed-Effect Forest Plot (TALA+ENZA vs. Active Treatments) for rPFS

**
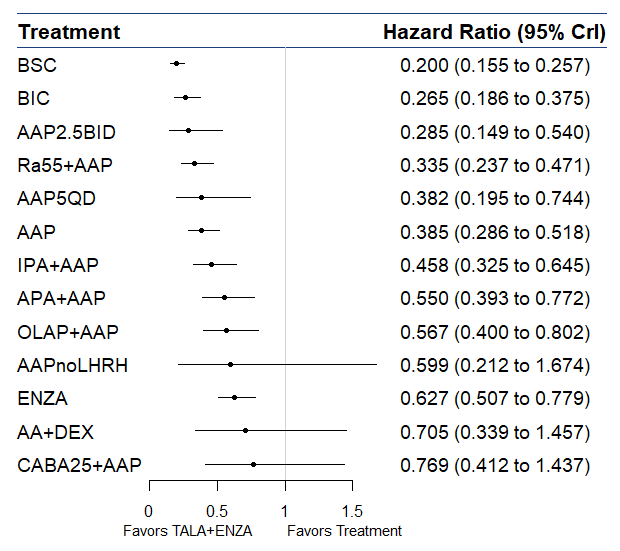
**

Abbreviations: Crl = credible interval; rPFS = radiographic progression-free survival.

Note: for full list of treatment regimens refer to  **Appendix B**.

Figure 25: Fixed-Effect Forest Plot (TALA+ENZA vs. Active Treatments) for OS

**
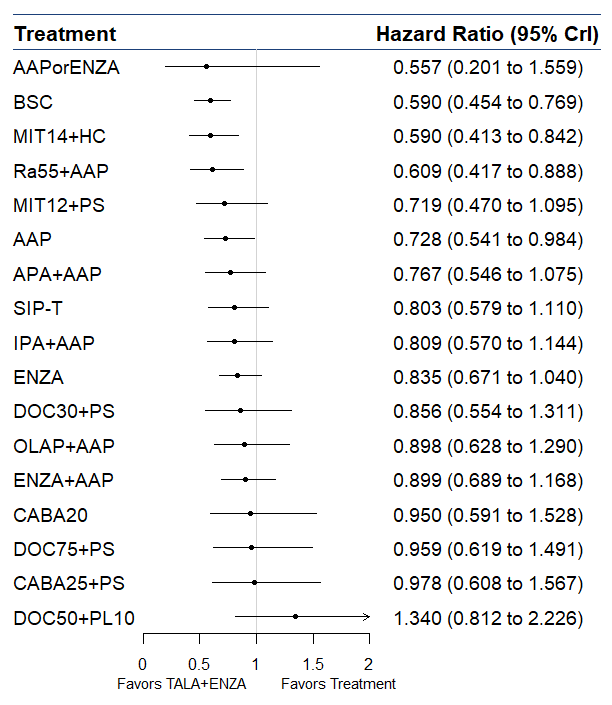
**

Abbreviations: Crl = credible interval; OS = overall survival.

Note: for full list of treatment regimens refer to  **Appendix B**.

Figure 26: Fixed-Effect Forest Plot (TALA+ENZA vs. Active Treatments) for Time to PSA Progression


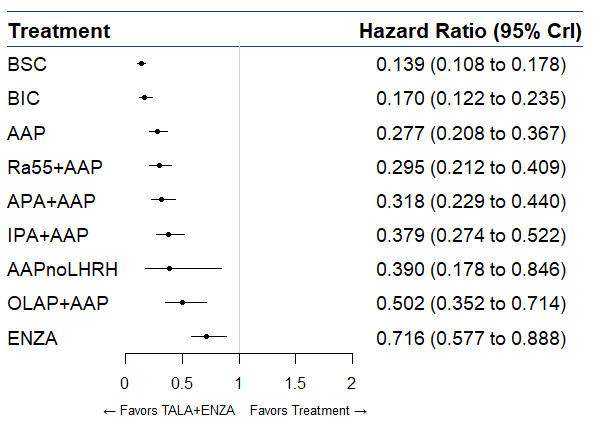


Abbreviations: Crl = credible interval; PSA = prostate specific antigen

Note: for full list of treatment regimens refer to  **Appendix B**.

Figure 27: Fixed-Effect Forest Plot (TALA+ENZA vs. Active Treatments) for Time to Cytotoxic Chemotherapy Initiation


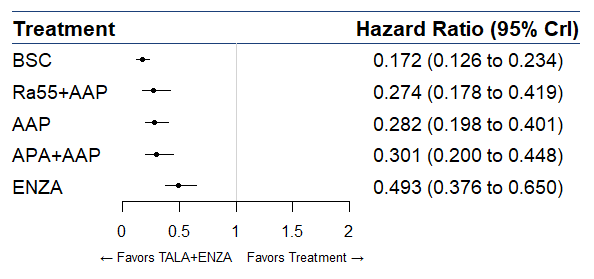
 Abbreviations: CrI = credible interval.

Note: for full list of treatment regimens refer to  **Appendix B**.

Figure 28: Fixed-Effect Forest Plot (TALA+ENZA vs. Active Treatments) for PSA Response


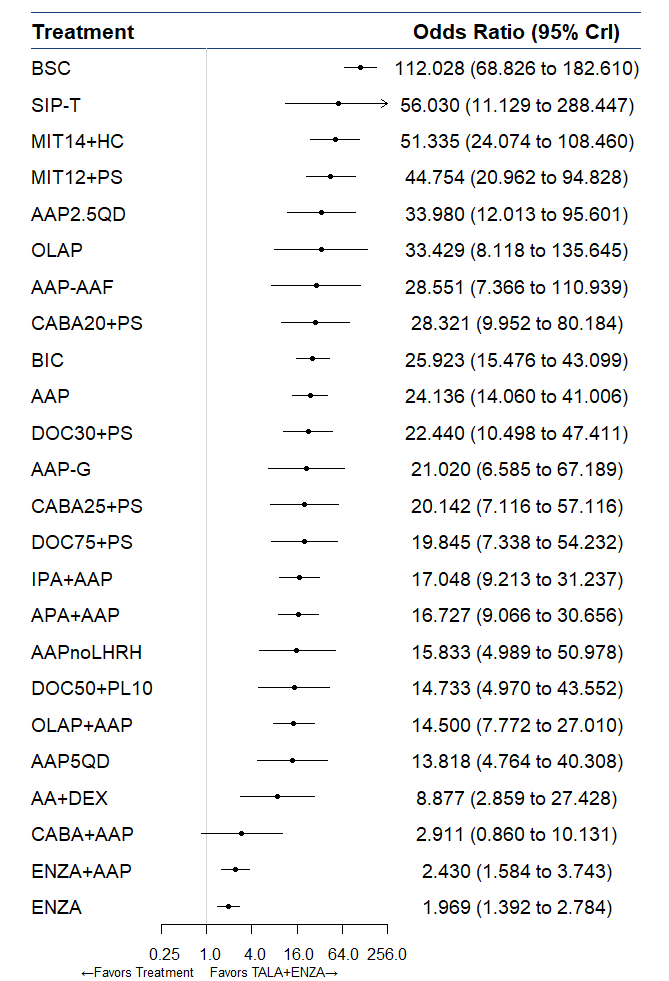


Abbreviations: CrI = credible interval; PSA = prostate specific antigen.

Note: for full list of treatment regimens refer to  **Appendix B**.

Figure 29: Fixed-Effect Forest Plot (TALA+ENZA vs. Active Treatments) for ORR


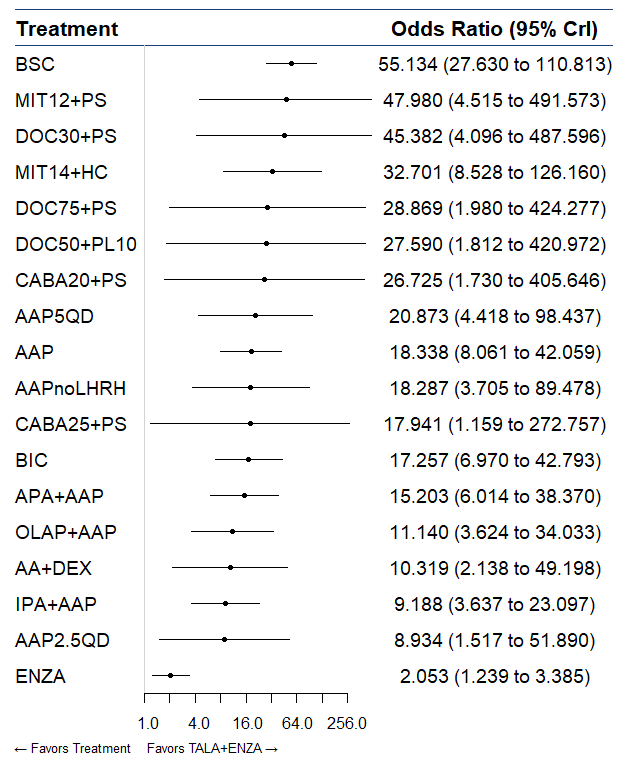


Abbreviations: CrI = credible interval; ORR = objective response rate.

Note: for full list of treatment regimens refer to  **Appendix B**.

Figure 30: Fixed-Effect Forest Plot (TALA+ENZA vs. Active Treatments) for All Grade Asthenia


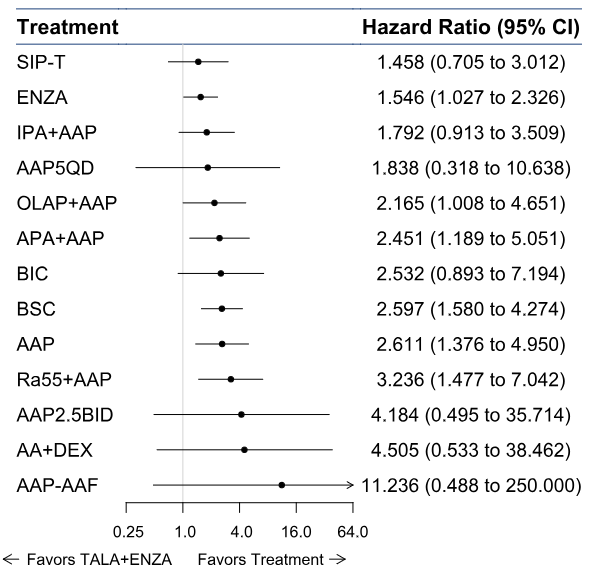


Abbreviations: CI = confidence interval.

Note: for full list of treatment regimens refer to  **Appendix B**.

Figure 31: Fixed-Effect Forest Plot (TALA+ENZA vs. Active Treatments) for All Grade Fatigue


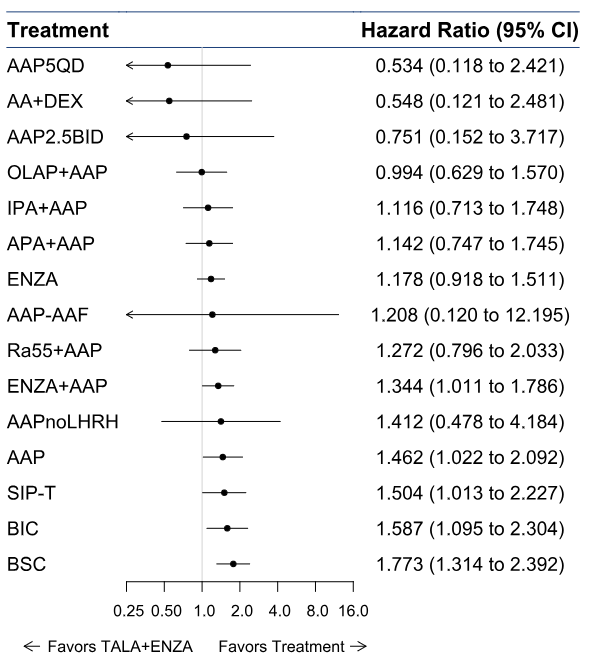


Abbreviations: CI = confidence interval.

Note: for full list of treatment regimens refer to  **Appendix B**.

Figure 32: Fixed-Effect Forest Plot (TALA+ENZA vs. Active Treatments) for All Grade Nausea


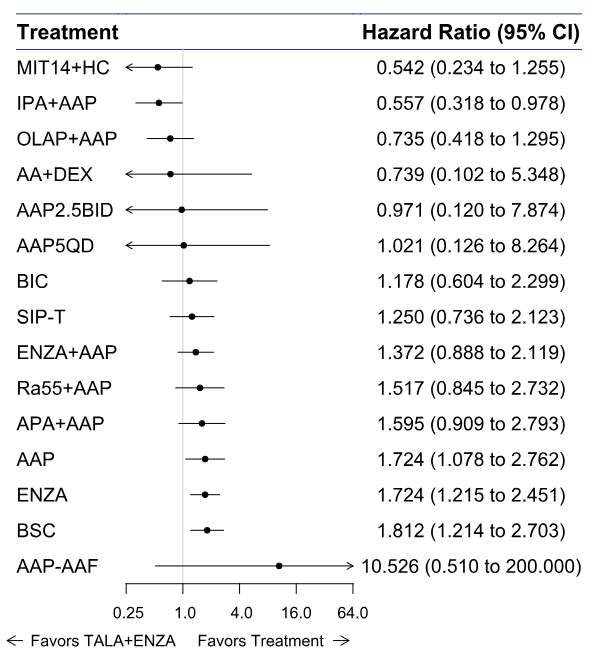


Abbreviations: CI = confidence interval.

Note: for full list of treatment regimens refer to  **Appendix B**.

Figure 33: Fixed-Effect Forest Plot (TALA+ENZA vs. Active Treatments) for All Grade Decreased Appetite


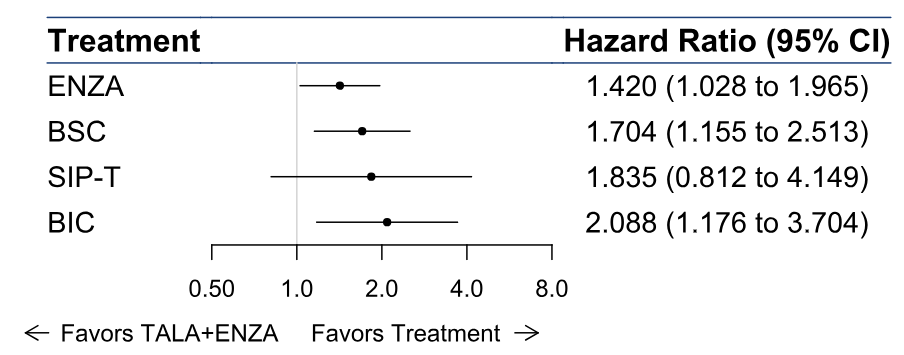


Abbreviations: CI = confidence interval.

Note: for full list of treatment regimens refer to  **Appendix B**.

Figure 34: Fixed-Effect League Table for rPFS


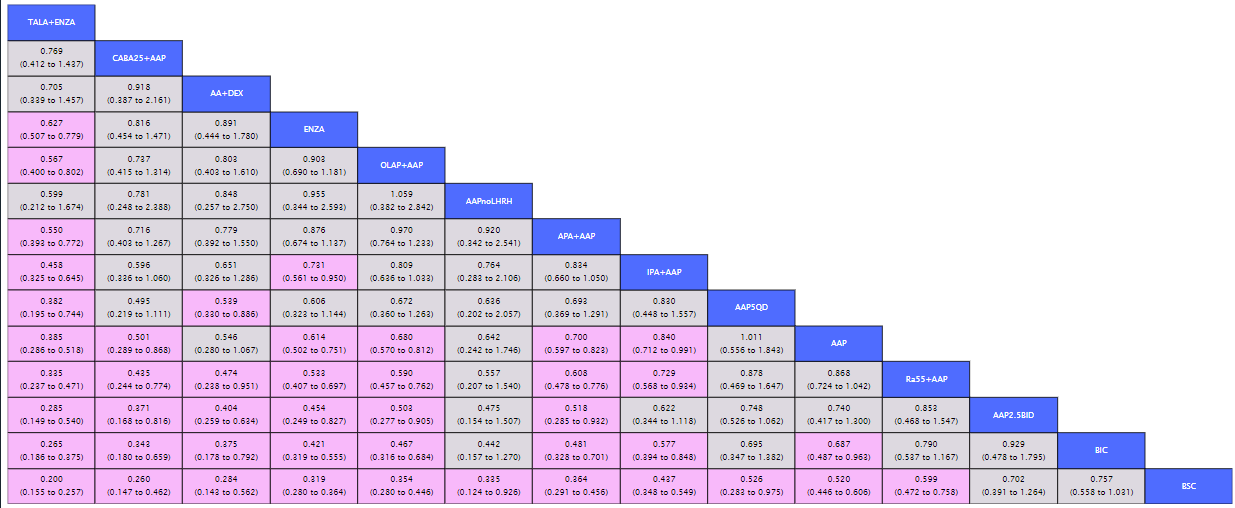


Note: Values are HRs (95% credible interval) for relative effectiveness for all possible pairs of treatments in the network

Note: HR < 1 implies that column is better than row

Note: Pink squares are statistically significant

Note: for full list of treatment regimens refer to **Appendix B**

Figure 35: Fixed-Effect League Table for OS


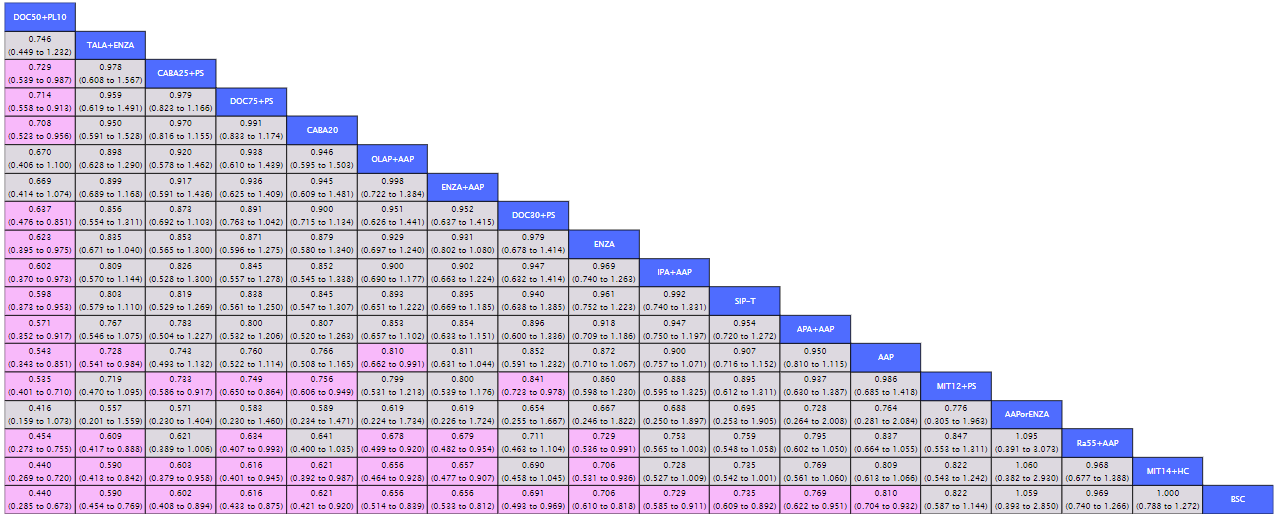


Note: Values are HRs (95% credible interval) for relative effectiveness for all possible pairs of treatments in the network

Note: HR < 1 implies that column is better than row

Note: Pink squares are statistically significant

Note: for full list of treatment regimens refer to **Appendix B**

Figure 36: Fixed-Effect League Table for Time to PSA Progression


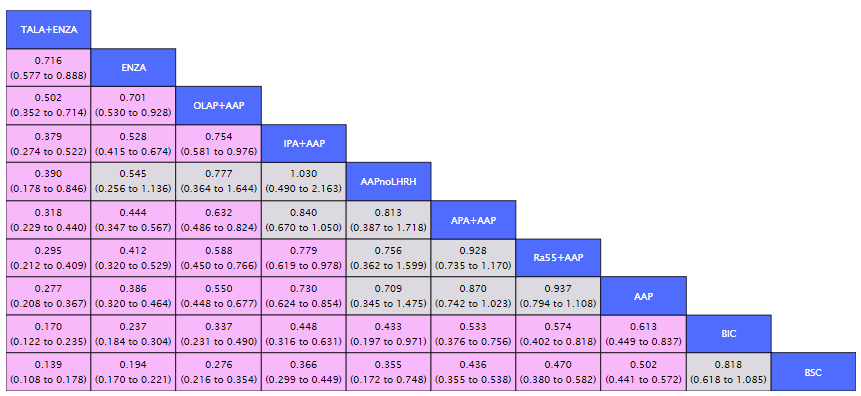


Note: Values are HRs (95% credible interval) for relative effectiveness for all possible pairs of treatments in the network

Note: HR < 1 implies that column is better than row

Note: Pink squares are statistically significant

Note: for full list of treatment regimens refer to **Appendix B**

Figure 37: Fixed-Effect League Table for Time to Cytotoxic Chemotherapy Initiation


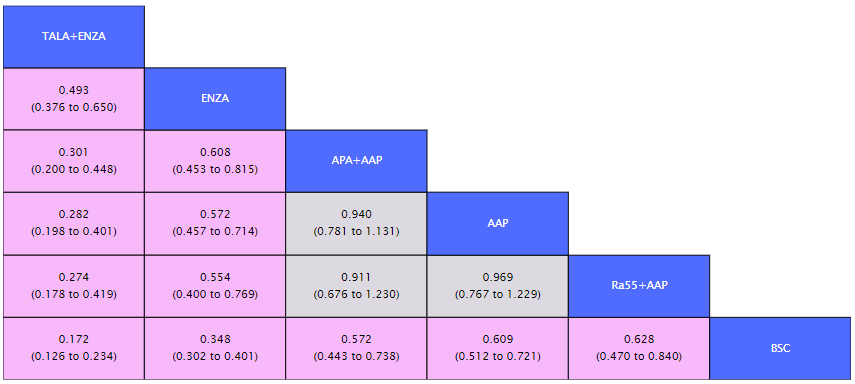


Note: Values are HRs (95% credible interval) for relative effectiveness for all possible pairs of treatments in the network

Note: HR < 1 implies that column is better than row

Note: Pink squares are statistically significant

Note: for full list of treatment regimens refer to **Appendix B**

Figure 38: Fixed-Effect League Table for PSA Response


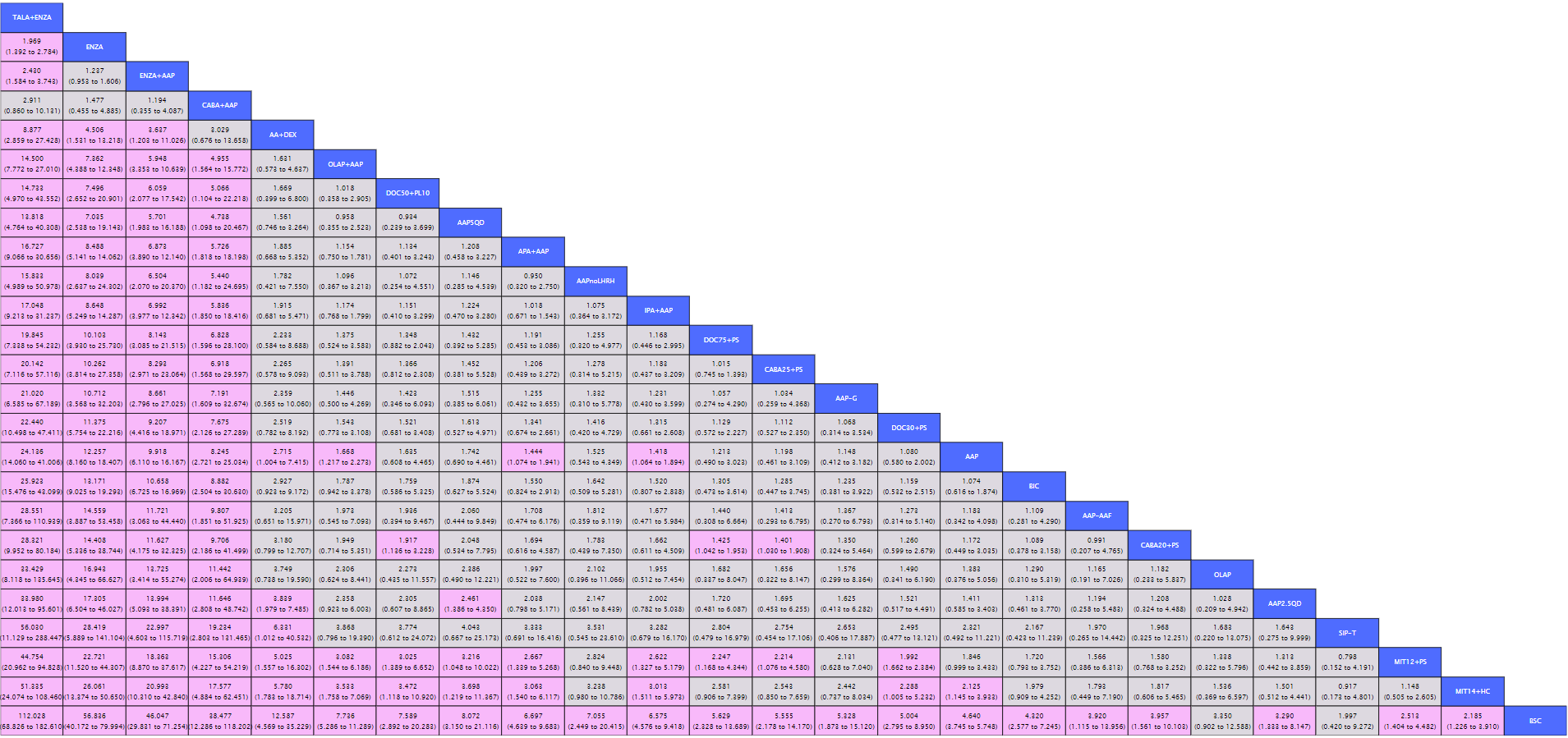


Note: Values are HRs (95% credible interval) for relative effectiveness for all possible pairs of treatments in the network

Note: HR < 1 implies that column is better than row

Note: Pink squares are statistically significant

Note: for full list of treatment regimens refer to **Appendix B**

Figure 39: Fixed-Effect League Table for ORR


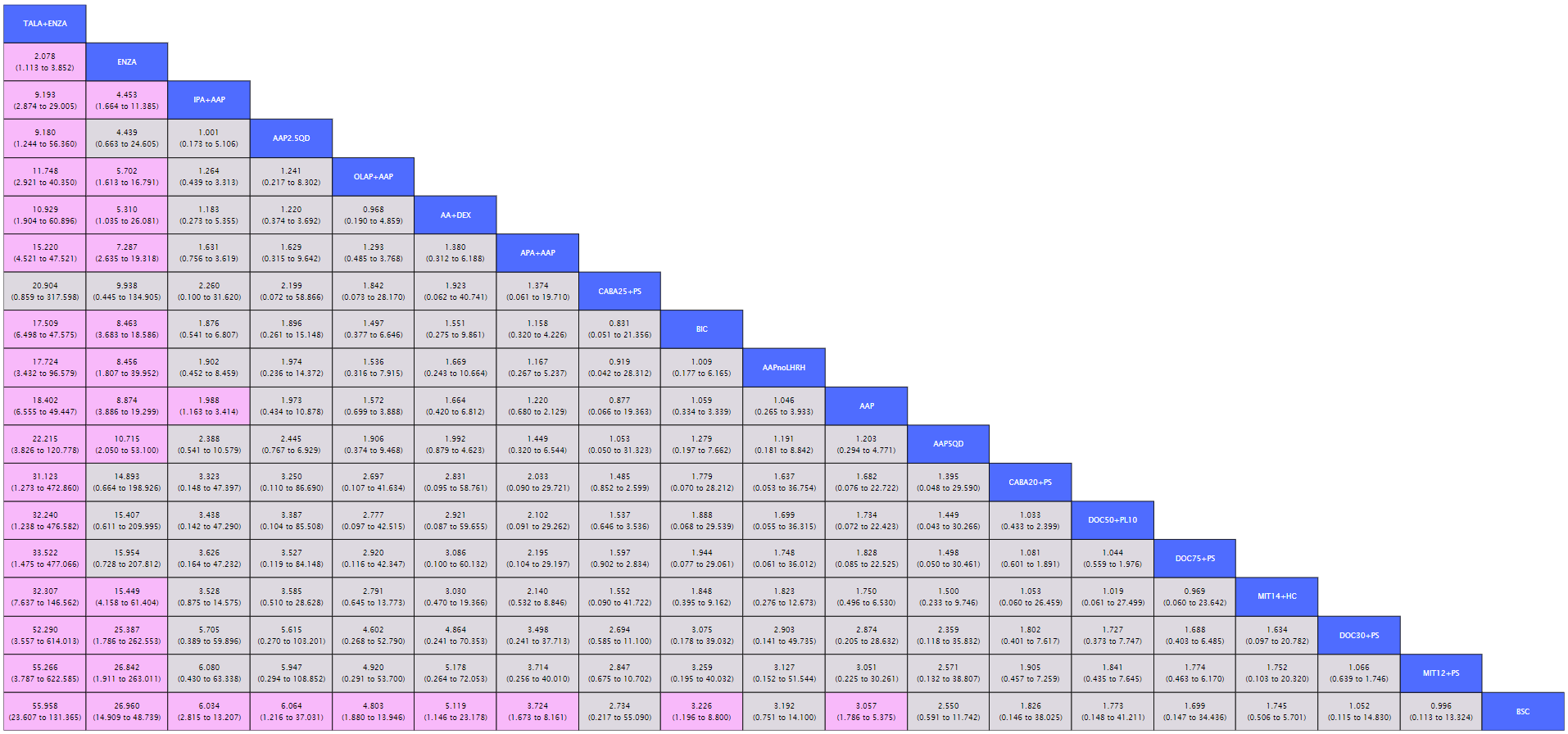


Note: Values are HRs (95% credible interval) for relative effectiveness for all possible pairs of treatments in the network

Note: HR < 1 implies that column is better than row

Note: Pink squares are statistically significant

Note: for full list of treatment regimens refer to **Appendix B**

Figure 40: Fixed-Effect League Table for Anemia (Grade ≥3)


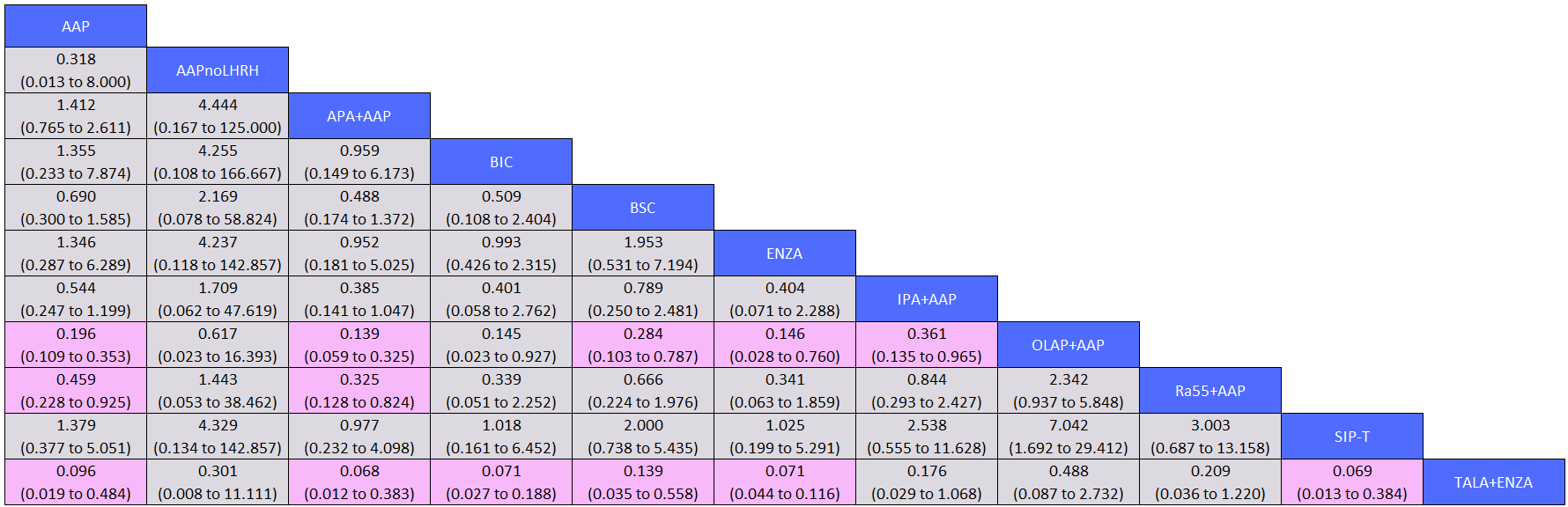


Note: Values are HRs (95% credible interval) for relative effectiveness for all possible pairs of treatments in the network

Note: HR < 1 implies that column is better than row

Note: Pink squares are statistically significant

Note: for full list of treatment regimens refer to **Appendix B**

Figure 41: Fixed-Effect League Table for Asthenia (All Grade)


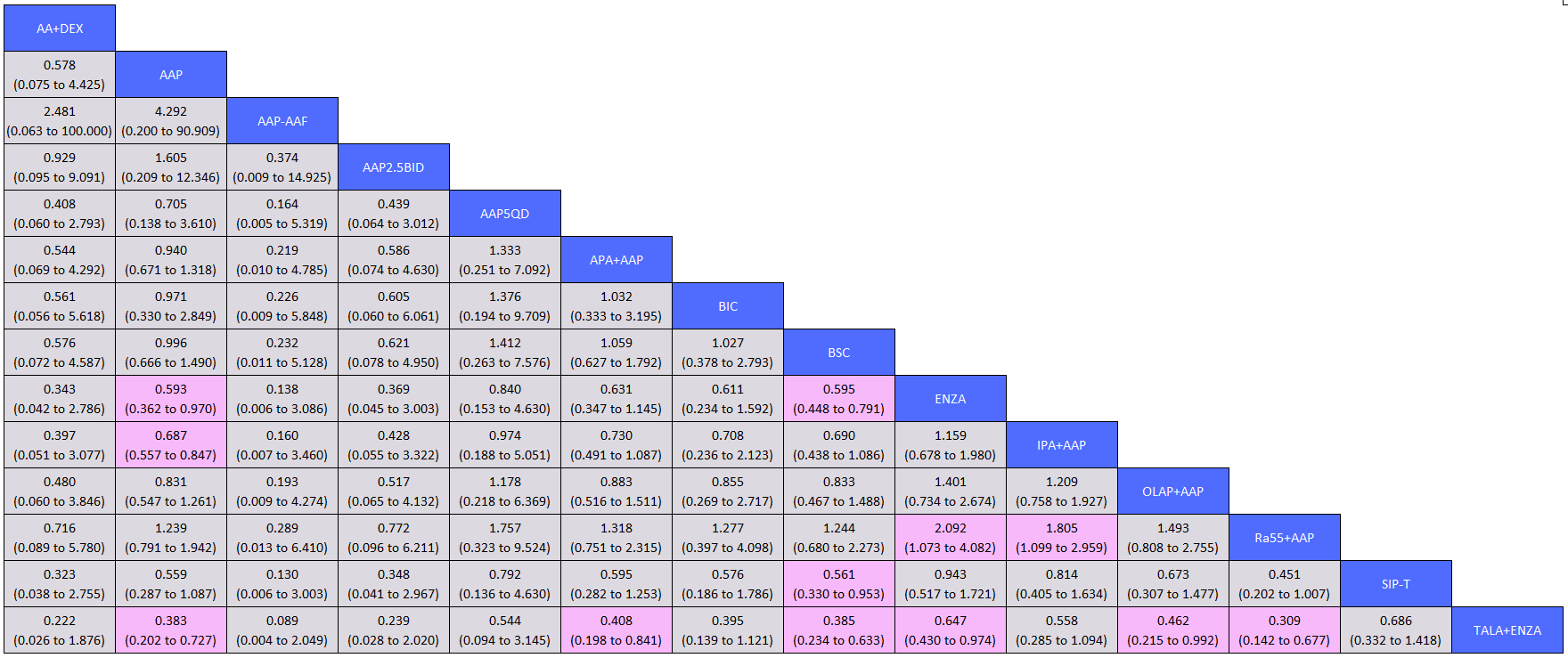


Note: Values are HRs (95% credible interval) for relative effectiveness for all possible pairs of treatments in the network

Note: HR < 1 implies that column is better than row

Note: Pink squares are statistically significant

Note: for full list of treatment regimens refer to **Appendix B**

Figure 42: Fixed-Effect League Table for Asthenia (Grade ≥3)


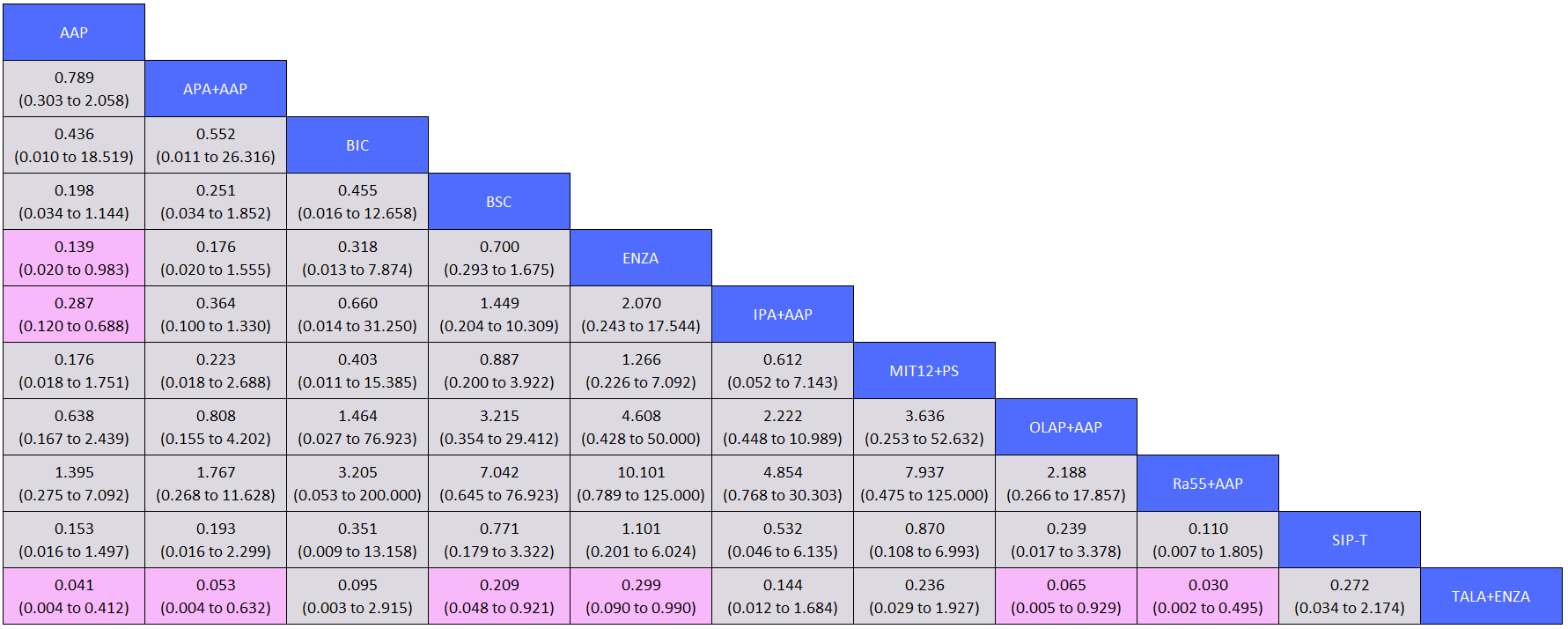


Note: Values are HRs (95% credible interval) for relative effectiveness for all possible pairs of treatments in the network

Note: HR < 1 implies that column is better than row

Note: Pink squares are statistically significant

Note: for full list of treatment regimens refer to **Appendix B**

Figure 43: Fixed-Effect League Table for Fatigue (All Grade)


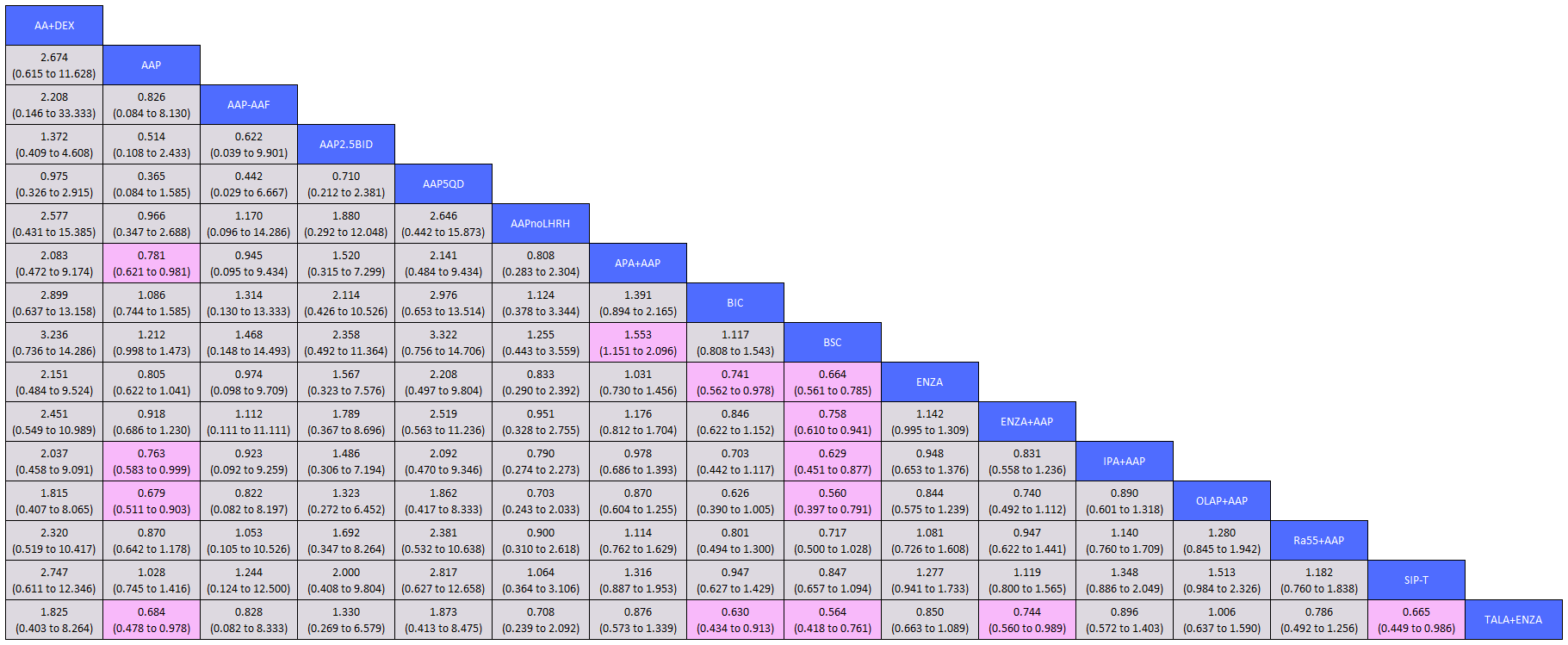


Note: Values are HRs (95% credible interval) for relative effectiveness for all possible pairs of treatments in the network

Note: HR < 1 implies that column is better than row

Note: Pink squares are statistically significant

Note: for full list of treatment regimens refer to **Appendix B**

Figure 44: Fixed-Effect League Table for Fatigue (Grade ≥3)


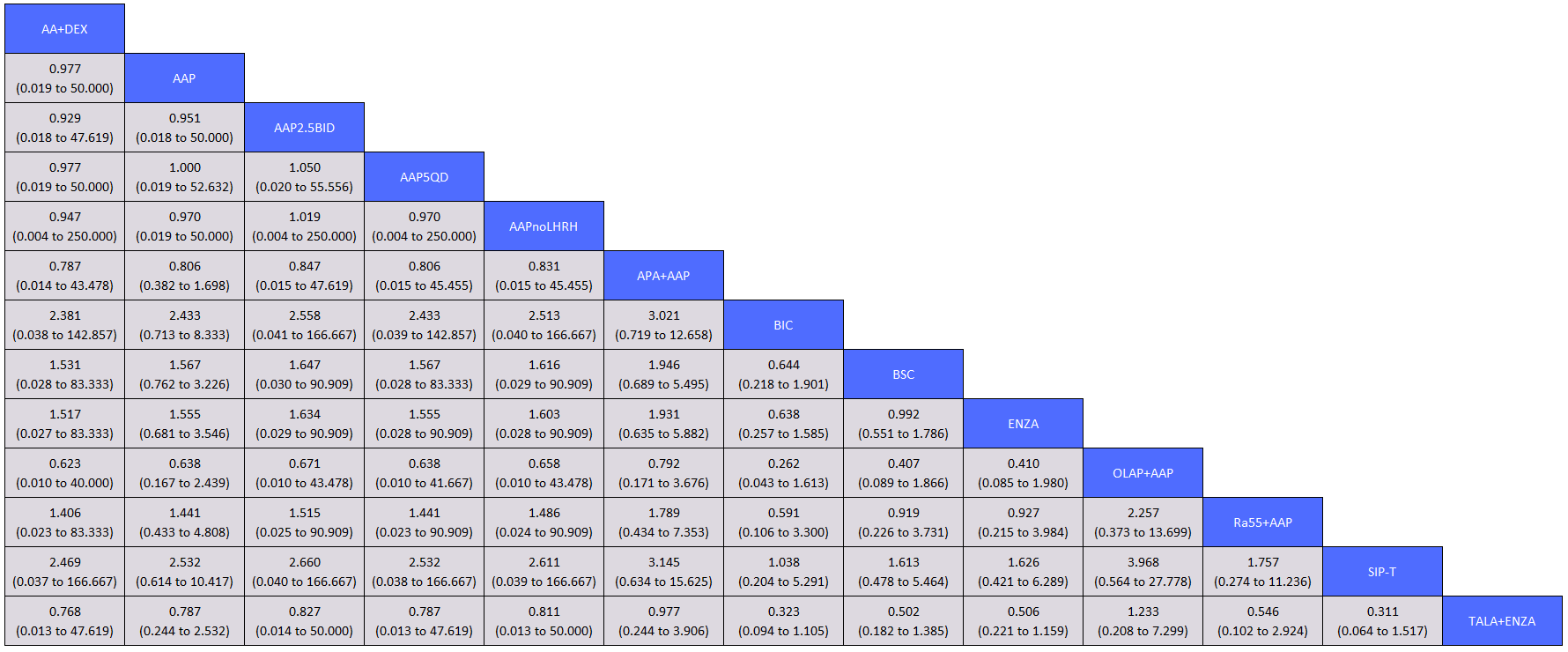


Note: Values are HRs (95% credible interval) for relative effectiveness for all possible pairs of treatments in the network

Note: HR < 1 implies that column is better than row

Note: Pink squares are statistically significant

Note: for full list of treatment regimens refer to **Appendix B**

Figure 45: Fixed-Effect League Table for Nausea (All Grade)


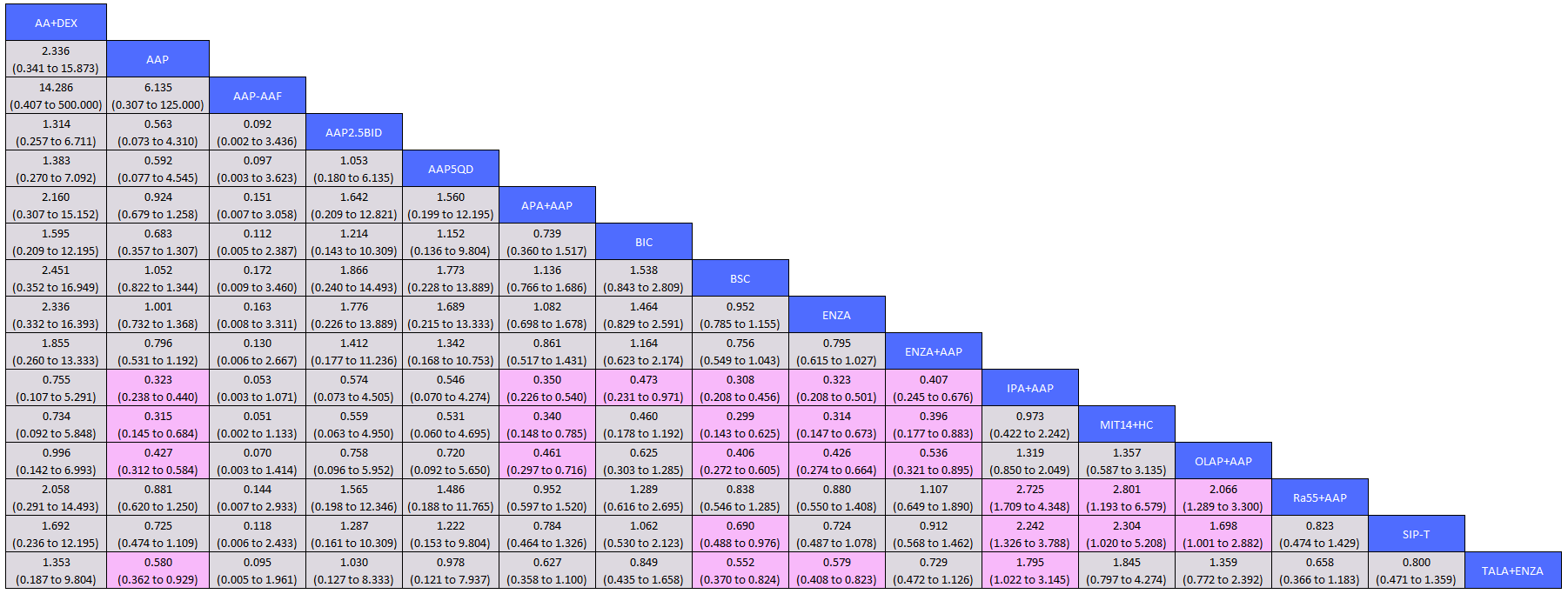


Note: Values are HRs (95% credible interval) for relative effectiveness for all possible pairs of treatments in the network

Note: HR < 1 implies that column is better than row

Note: Pink squares are statistically significant

Note: for full list of treatment regimens refer to **Appendix B**

Figure 46: Fixed-Effect League Table for Nausea (Grade ≥3)


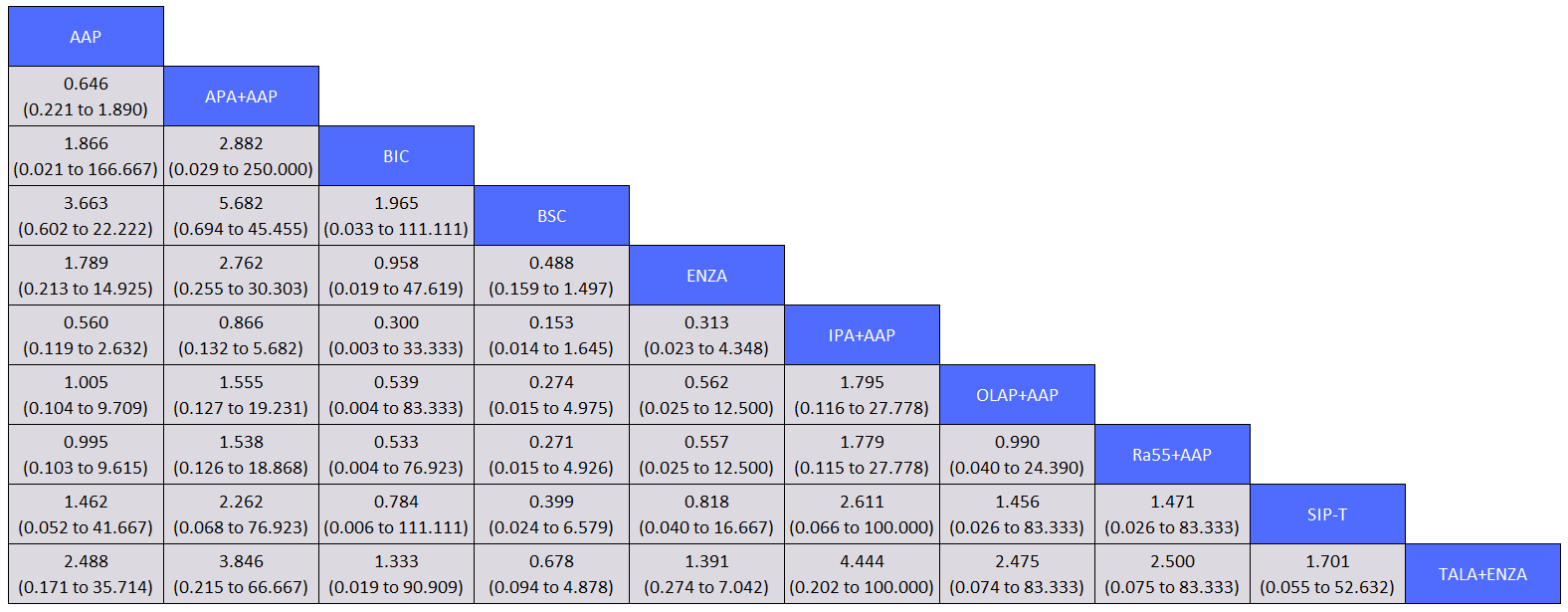


Note: Values are HRs (95% credible interval) for relative effectiveness for all possible pairs of treatments in the network

Note: HR < 1 implies that column is better than row

Note: Pink squares are statistically significant

Note: for full list of treatment regimens refer to **Appendix B**

Figure 47: Fixed-Effect League Table for Decreased Appetite (All Grade)


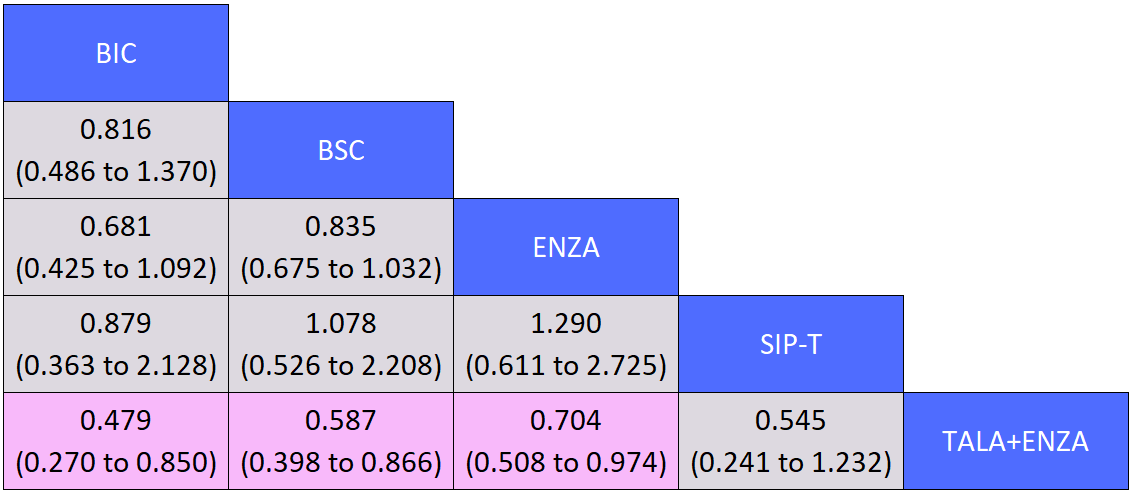


Note: Values are HRs (95% credible interval) for relative effectiveness for all possible pairs of treatments in the network

Note: HR < 1 implies that column is better than row

Note: Pink squares are statistically significant

Note: for full list of treatment regimens refer to **Appendix B**

Figure 48: Fixed-Effect League Table for Decreased Appetite (Grade ≥3)


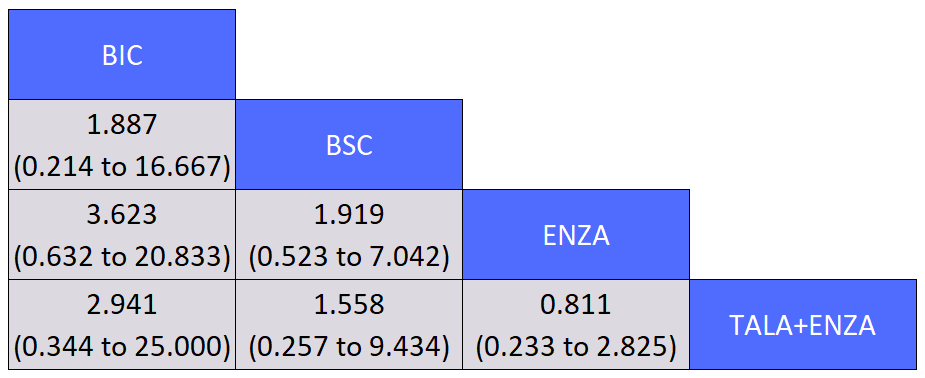


Note: Values are HRs (95% credible interval) for relative effectiveness for all possible pairs of treatments in the network

Note: HR < 1 implies that column is better than row

Note: Pink squares are statistically significant

Note: for full list of treatment regimens refer to **Appendix B**

1. Model Diagnostics

**Table 15: Model Diagnostics for rPFS**

| Model | | DIC | Total residual deviance | SD (95% Crl) |
| --- | --- | --- | --- | --- |
| Consistency | Fixed-effect | -13.51 | 15.69 | - |
|  | Random-effects | -14.01 | 15.22 | 0.12 (0.02, 0.45) |
| Inconsistency | Random-effects | -14.04 | 15.14 | 0.14 (0.02, 0.97) |

Note: a difference in the DIC between the models of more than ±3 was considered meaningful.

Abbreviations: CrI = credible interval; DIC = deviance information criterion; SD = standard deviation.

Table 16: Model Diagnostics for OS

| Model | | DIC | Total residual deviance | SD (95% Crl) |
| --- | --- | --- | --- | --- |
| Consistency | Fixed-effect | -25.04 | 24.96 | - |
|  | Random-effects | -26.93 | 23.23 | 0.11 (0.02, 0.34) |
| Inconsistency | Random-effects | -26.44 | 23.42 | 0.13 (0.02, 0.55) |

Note: a difference in the DIC between the models of more than ±3 was considered meaningful.

Abbreviations: CrI = credible interval; DIC = deviance information criterion; SD = standard deviation.

Table 17: Model Diagnostics for Time to PSA Progression

| Model | | DIC | Total residual deviance | SD (95% Crl) |
| --- | --- | --- | --- | --- |
| Consistency | Fixed-effect | 2.63 | 31.78 | - |
|  | Random-effects | -15.48 | 13.69 | 0.26 (0.11, 0.60) |
| Inconsistency | Random-effects | -15.55 | 13.67 | 0.26 (0.11, 0.58) |

Note: a difference in the DIC between the models of more than ±3 was considered meaningful.

Abbreviations: CrI = credible interval; DIC = deviance information criterion; SD = standard deviation.

Table 18: Model Diagnostics for Time to Cytotoxic Chemotherapy Initiation

| Model | | DIC | Total residual deviance | SD (95% Crl) |
| --- | --- | --- | --- | --- |
| Consistency | Fixed-effect | -5.92 | 5.43 | - |
|  | Random-effects | -5.77 | 5.58 | 0.12 (0.02, 0.50) |
| Inconsistency | Random-effects | -5.75 | 5.66 | 0.12 (0.02, 0.57) |

Note: a difference in the DIC between the models of more than ±3 was considered meaningful.

Abbreviations: CrI = credible interval; DIC = deviance information criterion; SD = standard deviation.

Table 19: Model Diagnostics for PSA Response

| Model | | DIC | Total residual deviance | SD (95% Crl) |
| --- | --- | --- | --- | --- |
| Consistency | Fixed-effect | 27.52 | 51.48 | - |
|  | Random-effects | 10.63 | 35.03 | 0.32 (0.07, 0.67) |
| Inconsistency | Random-effects | 11.03 | 34.94 | 0.33 (0.09, 0.66) |

Note: a difference in the DIC between the models of more than ±3 was considered meaningful.

Abbreviations: CrI = credible interval; DIC = deviance information criterion; SD = standard deviation.

Table 20: Model Diagnostics for ORR

| Model | | DIC | Total residual deviance | SD (95% Crl) |
| --- | --- | --- | --- | --- |
| Consistency | Fixed-effect | 21.45 | 21.05 | - |
|  | Random-effects | 21.44 | 20.81 | 0.12 (0.02, 0.47) |
| Inconsistency | Random-effects | 21.13 | 20.61 | 0.12 (0.02, 0.46) |

Note: a difference in the DIC between the models of more than ±3 was considered meaningful.

Abbreviations: CrI = credible interval; DIC = deviance information criterion; SD = standard deviation.

1. Sensitivity Analysis Removing PROSTY

**Rationale**

The PROSTY trial was a noninferiority trial by design and investigated the efficacy and safety of two different doses of docetaxel.[^44^](#_ENREF_44) The trial found the non-standard dose of docetaxel (50 mg/m^2^ every two weeks) demonstrated statistically superior OS compared to the standard dose (75 mg/m^2^ every three weeks).[^44^](#_ENREF_44) Importantly, relevant clinical guidelines do not mention or recommend docetaxel 50 mg/m^2^ over the standard docetaxel regimen or other standard of care treatments.[^45^](#_ENREF_45)^,^[^46^](#_ENREF_46) Moreover, the PROSTY trial determined that the non-standard dose may be appropriate for patients with comorbidities who are unlikely to tolerate large single doses of docetaxel.[^44^](#_ENREF_44) As such, a sensitivity analysis was conducted whereby the PROSTY trial was removed from the evidence base to explore the effect on results after removing this non-standard dose from the network.

Table 21: Random-Effects P-best and SUCRA Scores for OS (Sensitivity Analysis Removing PROSTY)

| **Treatment** | **Probability Best (%)** | **SUCRA (%)** |
| --- | --- | --- |
| TALA+ENZA | 32 | 81 |
| CABA25+PS | 16 | 75 |
| ENZA+AAP | 10 | 71 |
| CABA20 | 10 | 70 |
| OLAP+AAP | 10 | 65 |
| DOC75+PS | 7 | 74 |
| AAPorENZA | 7 | 28 |
| IPA+AAP | 3 | 52 |
| APA+AAP | 2 | 45 |
| ENZA | 1 | 62 |
| DOC30+PS | 1 | 58 |
| SIP-T | 1 | 52 |
| AAP | 0 | 36 |
| MIT12+PS | 0 | 32 |
| Ra55+AAP | 0 | 19 |
| MIT14+HC | 0 | 16 |
| BSC | 0 | 13 |

Abbreviations: OS = overall survival; SUCRA = surface under the cumulative ranking curve.

Note: for full list of treatment regimens refer to  **Appendix B**.

Figure 49: Random-Effects Forest Plot (TALA+ENZA vs. Active Treatments) for OS (Sensitivity Analysis Removing PROSTY)


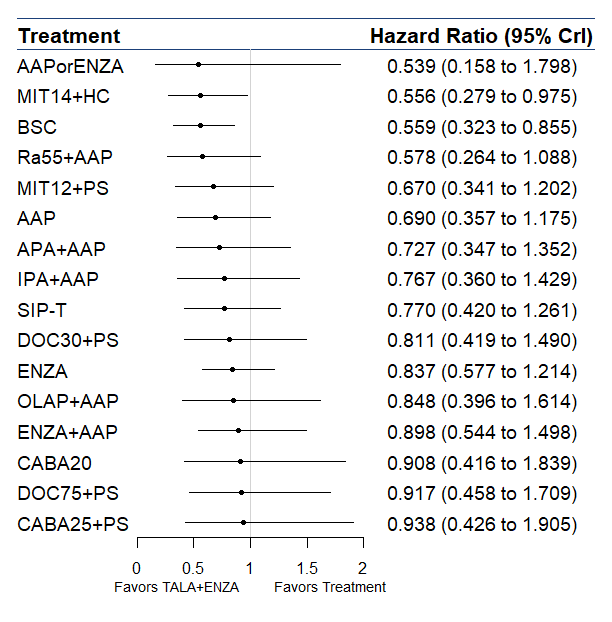


Abbreviations: Crl = credible interval; OS = overall survival.

Note: for full list of treatment regimens refer to  **Appendix B**.

Figure 50: Random-Effects League Table for OS (Sensitivity Analysis Removing PROSTY)


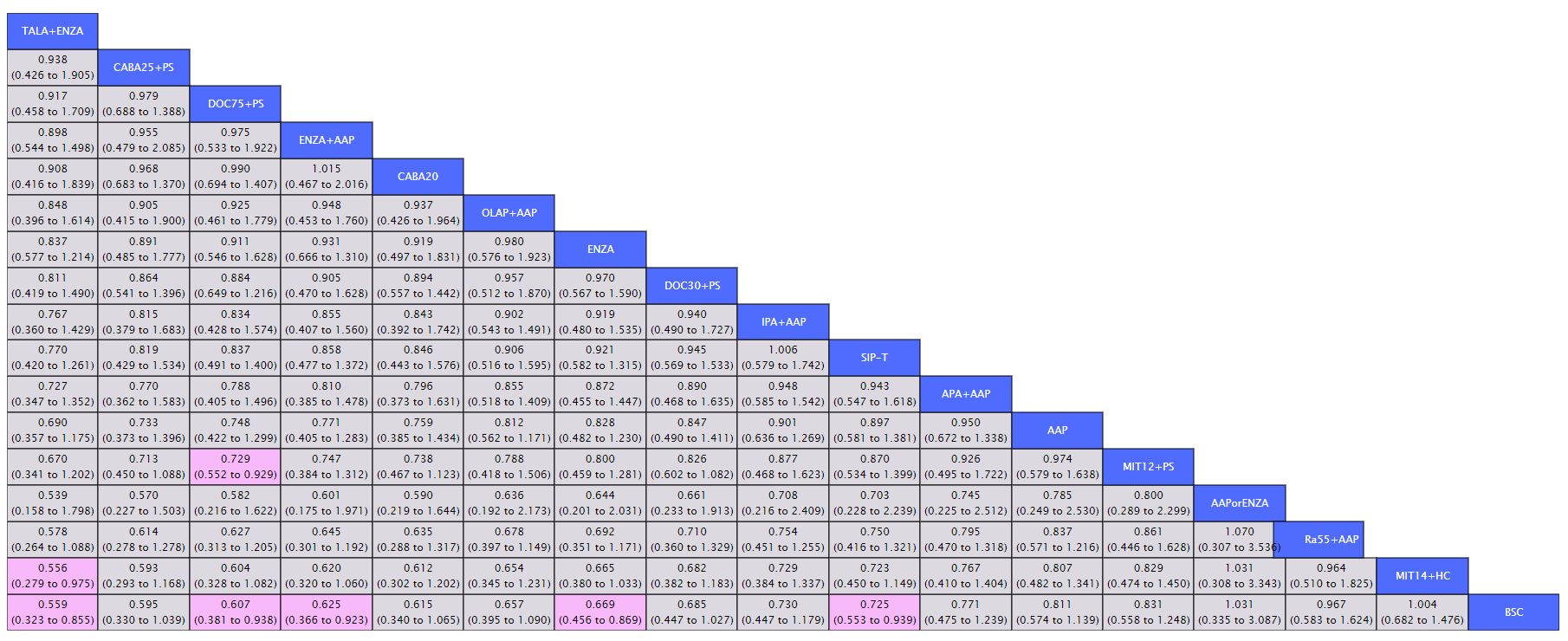


Note: Values are HRs (95% credible interval) for relative effectiveness for all possible pairs of treatments in the network

Note: HR < 1 implies that column is better than row

Note: Pink squares are statistically significant

Abbreviations: HR = hazard ratio; OS = overall survival.

Note: for full list of treatment regimens refer to **Appendix B**.

**Reference List**

1. Higgins JPT TJ, Chandler J, Cumpston M, Li T, Page MJ, Welch VA (editors). Cochrane handbook for systematic reviews of interventions version 6.2. John Wiley & Sons. Updated February 2021. [www.training.cochrane.org/handbook](file:///\\universal.co\BUON\public\Projects\Pfizer\Talazoparib%20-%20mCRPC%20First-line\European%20Urology%20Oncology%20Manuscript%202023\Files%20for%20submission\NMA\www.training.cochrane.org\handbook)

2. Montori VM, Wilczynski NL, Morgan D, Haynes RB, Hedges T. Optimal search strategies for retrieving systematic reviews from Medline: analytical survey. *BMJ*. Jan 8 2005;330(7482):68. doi:10.1136/bmj.38336.804167.47

3. Ovid. Ovid Tools and Resources Portal - Expert Searches. Accessed Spetember 9, 2021, <https://tools.ovid.com/ovidtools/expertsearches.html>

4. McGowan J, Sampson M, Salzwedel DM, Cogo E, Foerster V, Lefebvre C. PRESS peer review of electronic search strategies: 2015 guideline statement. *Journal of clinical epidemiology*. 2016;75:40-46.

5. Clarke NW, Armstrong AJ, Thiery-Vuillemin A, et al. Final overall survival (OS) in PROpel: Abiraterone (abi) and olaparib (ola) versus abiraterone and placebo (pbo) as first-line (1L) therapy for metastatic castration-resistant prostate cancer (mCRPC). American Society of Clinical Oncology; 2023.

6. Efstathiou E, Smith MR, Sandhu S, et al. Niraparib (NIRA) with abiraterone acetate and prednisone (AAP) in patients (pts) with metastatic castration-resistant prostate cancer (mCRPC) and homologous recombination repair (HRR) gene alterations: Second interim analysis (IA2) of MAGNITUDE. American Society of Clinical Oncology; 2023.

7. Chi K, Sandhu S, Smith M, et al. Niraparib plus abiraterone acetate with prednisone in patients with metastatic castration-resistant prostate cancer and homologous recombination repair gene alterations: second interim analysis of the randomized phase III MAGNITUDE trial. *Annals of Oncology*. 2023;

8. Chi KN, Rathkopf DE, Smith MR, et al. Phase 3 MAGNITUDE study: First results of niraparib (NIRA) with abiraterone acetate and prednisone (AAP) as first-line therapy in patients (pts) with metastatic castration-resistant prostate cancer (mCRPC) with and without homologous recombination repair (HRR) gene alterations. American Society of Clinical Oncology; 2022.

9. Agarwal N, Azad AA, Carles J, et al. Talazoparib plus enzalutamide in men with first-line metastatic castration-resistant prostate cancer (TALAPRO-2): a randomised, placebo-controlled, phase 3 trial. *The Lancet*. 2023;

10. NICE. *Single technology appraisal and highly specialised technologies evaluation: User guide for company evidence submission template*. 2015. <https://www.nice.org.uk/process/pmg24/chapter/clinical-effectiveness#quality-assessment-of-the-relevant-clinical-effectiveness-evidence>

11. McCool R, Fleetwood K, Glanville J, Arber M, Goodall H, Naidoo S. Systematic review and network meta-analysis of treatments for chemotherapy-naive patients with asymptomatic/mildly symptomatic metastatic castration-resistant prostate cancer. *Value in Health*. 2018;21(10):1259-1268.

12. Page MJ, McKenzie JE, Bossuyt PM, et al. The PRISMA 2020 statement: an updated guideline for reporting systematic reviews. *BMJ*. Mar 29 2021;372:n71. doi:10.1136/bmj.n71

13. Ryan CJ, Small EJ, Smith MR, et al. Abiraterone in metastatic prostate cancer without previous chemotherapy. *New England Journal of Medicine*. 2013;368(2):138-148. doi:<http://dx.doi.org/10.1056/NEJMoa1209096>

14. Ye D, Huang Y, Zhou F, et al. A phase 3, double-blind, randomized placebo-controlled efficacy and safety study of abiraterone acetate in chemotherapy-naive patients with mCRPC in China, Malaysia, Thailand and Russia. *Asian Journal of Urology*. 2017;4(2):75-85. doi:<http://dx.doi.org/10.1016/j.ajur.2017.01.002>

15. Attard G, Merseburger AS, Arlt W, et al. Assessment of the Safety of Glucocorticoid Regimens in Combination with Abiraterone Acetate for Metastatic Castration-Resistant Prostate Cancer: A Randomized, Open-label Phase 2 Study. *JAMA Oncology*. 2019;5(8):1159-1167. doi:<http://dx.doi.org/10.1001/jamaoncol.2019.1011>

16. Stein CA, Levin R, Given R, et al. Randomized phase 2 therapeutic equivalence study of abiraterone acetate fine particle formulation vs. originator abiraterone acetate in patients with metastatic castration-resistant prostate cancer: The STAAR study. *Urologic Oncology: Seminars and Original Investigations*. 2018;36(2):81. doi:<http://dx.doi.org/10.1016/j.urolonc.2017.10.018>

17. Beer TM, Armstrong AJ, Rathkopf DE, et al. Enzalutamide in metastatic prostate cancer before chemotherapy. *New England Journal of Medicine*. 2014;371(5):424-433. doi:<http://dx.doi.org/10.1056/NEJMoa1405095>

18. Shore ND, Chowdhury S, Villers A, et al. Efficacy and safety of enzalutamide versus bicalutamide for patients with metastatic prostate cancer (TERRAIN): A randomised, double-blind, phase 2 study. *The Lancet Oncology*. 2016;17(2):153-163. doi:<http://dx.doi.org/10.1016/S1470-2045%2815%2900518-5>

19. Penson DF, Concepcion R, Armstrong AJ, et al. Enzalutamide versus bicalutamide in castration-resistant prostate cancer: The STRIVE trial. *Journal of Clinical Oncology*. 2016;34(18):2098-2106. doi:<http://dx.doi.org/10.1200/JCO.2015.64.9285>

20. Khalaf DJ, Kollmannsberger CK, Eigl BJ, et al. Optimal sequencing of enzalutamide and abiraterone acetate plus prednisone in metastatic castration-resistant prostate cancer: a multicentre, randomised, open-label, phase 2, crossover trial. *The Lancet Oncology*. 2019;20(12):1730-1739. doi:<http://dx.doi.org/10.1016/S1470-2045%2819%2930688-6>

21. Saad F, Efstathiou E, Attard G, et al. Apalutamide plus abiraterone acetate and prednisone versus placebo plus abiraterone and prednisone in metastatic, castration-resistant prostate cancer (ACIS): a randomised, placebo-controlled, double-blind, multinational, phase 3 study. *The Lancet Oncology*. 2021;22(11):1541-1559.

22. Kantoff P, Halabi SCMPJKJHVTDWEPVNJ. Hydrocortisone with or without mitoxantrone in men with hormone-refractory prostate cancer: results of the cancer and leukemia group B 9182 study. *Journal of clinical oncology*. 1999;17(8):2506.

23. Berry W, Dakhil S, Modiano M, Gregurich M, Asmar L. Phase III study of mitoxantrone plus low dose prednisone versus low dose prednisone alone in patients with asymptomatic horlone refractory prostate cancer. *Journal of Urology*. 2002;168(6):2439-2443. doi:<http://dx.doi.org/10.1016/s0022-5347%2805%2964163-8>

24. De Wit R, Berry WR, Horti J, et al. Docetaxel plus prednisone or mitoxantrone plus prednisone for advanced prostate cancer. *New England Journal of Medicine*. 2004;351(15):1502-1512. doi:<http://dx.doi.org/10.1056/NEJMoa040720>

25. Zhou T, Zeng S-X, Sun Y-H, et al. A multicenter, randomized clinical trial comparing the three-weekly docetaxel regimen plus prednisone versus mitoxantone plus prednisone for Chinese Patients with metastatic castration refractory prostate cancer. *PLoS ONE*. 2015;10(1):e0117002. doi:<http://dx.doi.org/10.1371/journal.pone.0117002>

26. Caffo O, Veccia A, Maines F, et al. Intermittent docetaxel chemotherapy as first-line treatment for metastatic castration-resistant prostate cancer patients. *Future Oncology*. 2015;11(6):965-973. doi:<http://dx.doi.org/10.2217/fon.14.284>

27. Kellokumpu-Lehtinen P-L, Hervonen P, Lehtinen I, et al. 2-weekly versus 3-weekly docetaxel to treat castration-resistant advanced prostate cancer: A randomised, Phase 3 trial. *The Lancet Oncology*. 2013;14(2):117-124. doi:<http://dx.doi.org/10.1016/S1470-2045%2812%2970537-5>

28. Cash H, Steiner U, Fuller F, et al. Intermittent vs continuous docetaxel therapy in patients with metastatic castration-resistant prostate cancer - a phase III study (PRINCE). *BJU International*. 2018;122(5):774-782. doi:<http://dx.doi.org/10.1111/bju.14239>

29. Fosså SD, Jacobsen A-B, Ginman C, et al. Weekly docetaxel and prednisolone versus prednisolone alone in androgen-independent prostate cancer: a randomized phase II study. *European urology*. 2007;52(6):1691-1699.

30. Oudard S, Chadjaa M, Fizazi K, et al. Cabazitaxel versus docetaxel as first-line therapy for patients with metastatic castration-resistant prostate cancer: A randomized phase III trial - FIRSTANA. *Journal of Clinical Oncology*. 2017;35(28):3189-3197. doi:<http://dx.doi.org/10.1200/JCO.2016.72.1068>

31. Annala M, Fu S, Bacon J, et al. Cabazitaxel versus abiraterone or enzalutamide in poor prognosis metastatic castration-resistant prostate cancer: a multicentre, randomised, open-label, phase II trial. *Annals of Oncology*. 2021;32(7):896-905.

32. Small EJ, Schellhammer PF, Higano CS, et al. Placebo-controlled phase III trial of immunologic therapy with Sipuleucel-T (APC8015) in patients with metastatic, asymptomatic hormone refractory prostate cancer. *Journal of Clinical Oncology*. 2006;24(19):3089-3094. doi:<http://dx.doi.org/10.1200/JCO.2005.04.5252>

33. Higano CS, Schellhammer PF, Small EJ, et al. Integrated data from 2 randomized, double‐blind, placebo‐controlled, phase 3 trials of active cellular immunotherapy with sipuleucel‐T in advanced prostate cancer. *Cancer: Interdisciplinary International Journal of the American Cancer Society*. 2009;115(16):3670-3679.

34. Kantoff PW, Higano CS, Shore ND, et al. Sipuleucel-T immunotherapy for castration-resistant prostate cancer. *New England Journal of Medicine*. 2010;363(5):411-422. doi:<http://dx.doi.org/10.1056/NEJMoa1001294>

35. Small EJ, Fong L, Lance RS, et al. A randomized phase II trial of sipuleucel-T with concurrent versus sequential abiraterone acetate plus prednisone in metastatic castration-resistant prostate cancer. *Clinical Cancer Research*. 2015;21(17):3862-3869. doi:<http://dx.doi.org/10.1158/1078-0432.CCR-15-0079>

36. Parker CC, Pascoe S, Chodacki A, et al. A randomized, double-blind, dose-finding, multicenter, phase 2 study of radium chloride (Ra 223) in patients with bone metastases and castration-resistant prostate cancer. *European Urology*. 2013;63(2):189-197. doi:<http://dx.doi.org/10.1016/j.eururo.2012.09.008>

37. Smith M, Parker C, Saad F, et al. Addition of radium-223 to abiraterone acetate and prednisone or prednisolone in patients with castration-resistant prostate cancer and bone metastases (ERA 223): a randomised, double-blind, placebo-controlled, phase 3 trial. *The Lancet Oncology*. 2019;20(3):408-419. doi:<http://dx.doi.org/10.1016/S1470-2045%2818%2930860-X>

38. Sweeney C, Bracarda S, Sternberg CN, et al. Ipatasertib plus abiraterone and prednisolone in metastatic castration-resistant prostate cancer (IPATential150): a multicentre, randomised, double-blind, phase 3 trial. *The Lancet*. 2021;398(10295):131-142. doi:<http://dx.doi.org/10.1016/S0140-6736%2821%2900580-8>

39. Pu YS, Ahn H, Han W, et al. Enzalutamide in Chemotherapy-Na&iuml;ve Metastatic Castration-Resistant Prostate Cancer: An Asian Multiregional, Randomized Study. *Advances in therapy*. 2022;39(6):2641. doi:<https://doi.org/10.1007/s12325-022-02140-2>

40. Ohlmann CH, Jaschke M, Jaehnig P, et al. LHRH sparing therapy in patients with chemotherapy-na&iuml;ve, mCRPC treated with abiraterone acetate plus prednisone: results of the randomized phase II SPARE trial. *Prostate cancer and prostatic diseases*. 2022;doi:<https://doi.org/10.1038/s41391-022-00533-6>

41. Clarke Noel W, Armstrong Andrew J, Thiery-Vuillemin A, et al. Abiraterone and Olaparib for Metastatic Castration-Resistant Prostate Cancer. *NEJM Evidence*. 2022/08/23 2022;1(9):EVIDoa2200043. doi:10.1056/EVIDoa2200043

42. Hu Z, Ye Z, Zeng H, et al. Abiraterone acetate in men with metastatic castration-resistant prostate cancer and no prior chemotherapy: A double-arm, multiple-centre, phase III clinical study. *Annals of Oncology*. 2020;31(Supplement 4):S530. ESMO Virtual Congress 2020. Virtual, Online. doi:<http://dx.doi.org/10.1016/j.annonc.2020.08.908>

43. Slovin SF, Knudsen KE, Halabi S, et al. Abiraterone acetate (AA) with or without cabazitaxel (CBZ) in treatment of chemotherapy naive metastatic castration-resistant prostate cancer (mCRPC). American Society of Clinical Oncology; 2020.

44. Kellokumpu-Lehtinen P-L, Harmenberg U, Joensuu T, et al. 2-Weekly versus 3-weekly docetaxel to treat castration-resistant advanced prostate cancer: a randomised, phase 3 trial. *The lancet oncology*. 2013;14(2):117-124.

45. Lowrance W, Dreicer R, Jarrard DF, et al. Updates to Advanced Prostate Cancer: AUA/SUO Guideline (2023). *The Journal of Urology*. 2023;209(6):1082-1090.

46. Virgo KS, Rumble RB, de Wit R, et al. Initial management of noncastrate advanced, recurrent, or metastatic prostate cancer: ASCO guideline update. *Journal of Clinical Oncology*. 2021;39(11):1274-1305.
